# Supplementary material for: Vegetation trends associated with urban development: The role of golf courses
Source: PLoS One. 2020 Feb 5;15(2):e0228090. doi: 10.1371/journal.pone.0228090 (PMC7001943; doi:10.1371/journal.pone.0228090)

# PLOS ONE


## Trends in vegetation dynamics associated with urban development: The role of golf courses

--Manuscript Draft--

| | |
|---|---|
| **Manuscript Number:** | PONE-D-19-19510 |
| **Article Type:** | Research Article |
| **Full Title:** | Trends in vegetation dynamics associated with urban development: The role of golf courses |
| **Short Title:** | Urban development and the role of golf courses |
| **Corresponding Author:** | Thu T Nguyen<br>Vietnam National University of Forestry<br>Ha Noi, VIET NAM |
| **Keywords:** | Urban ecosystems, vegetation change, biodiversity connectivity, biodiversity hotpot, golf courses, remote sensing |
| **Abstract:** | Globally, cities have grown rapidly in size and density and this has caused profound impacts on urban forest ecosystems. Urbanization requiring clearing of vegetation reduces ecosystem services that benefit both city dwellers and biodiversity. Understanding spatial and temporal patterns of vegetation changes associated with urbanization is vital for future sustainable urban development. We used Landsat time series data for the period 1988 to 2018 to characterize changes in vegetation cover and habitat connectivity in the Perth Metropolitan Area, a rapidly urbanising biodiversity hotspot, as a case study to understand the impacts of urbanization on urban forests. Moreover, as golf courses are rapidly increasing in many urban areas, we assessed the role of golf courses in maintaining vegetation cover and creating habitat connectivity. We employed (1) land use classification, post-classification change detection, and (2) Morphological Spatial Pattern Analysis (MSPA). Over 17,000 hectares of vegetation were cleared and the area of vegetation contributing to biodiversity connectivity was reduced significantly over the three decades. The spatial patterns of vegetation loss and gain were different in each of the three decades reflecting the implementation of urban planning. Furthermore, MSPA analysis showed that the reduction in vegetation cover led to habitat fragmentation with a significant decrease in the core and bridge classes and an increase in isolated patches in the urban landscape. Golf courses played a useful role in maintaining vegetation cover and contributing to connectivity in a regional biodiversity hotspot. Our findings suggest that urban planning needs to more carefully consider land clearing and its impacts on connectivity in the landscape. Moreover, there is a need to take into consideration opportunities for off-reserve conservation in smaller habitat fragments such as in golf courses in sustainable urban management. |
| **Order of Authors:** | Thu Thi Nguyen |
| | Paul Barber |
| | Richard Harper |
| | Tran Vu Khanh Linh |
| | Bernard Dell |
| **Additional Information:** | |
| **Question** | **Response** |
| **Financial Disclosure**<br><br>Enter a financial disclosure statement that describes the sources of funding for the work included in this submission. Review the submission guidelines for detailed | TTN received PhD scholarship co-funded by Vietnam and Murdoch University. |

| | |
|---|---|
| requirements. View published research articles from *PLOS ONE* for specific examples.<br><br>This statement is required for submission and **will appear in the published article** if the submission is accepted. Please make sure it is accurate.<br><br>**Unfunded studies**<br>Enter: *The author(s) received no specific funding for this work.*<br><br>**Funded studies**<br>Enter a statement with the following details:<br>• Initials of the authors who received each award<br>• Grant numbers awarded to each author<br>• The full name of each funder<br>• URL of each funder website<br>• Did the sponsors or funders play any role in the study design, data collection and analysis, decision to publish, or preparation of the manuscript?<br>• **NO** - Include this sentence at the end of your statement: *The funders had no role in study design, data collection and analysis, decision to publish, or preparation of the manuscript.*<br>• **YES** - Specify the role(s) played.<br><br>* typeset | |
| **Competing Interests**<br><br>Use the instructions below to enter a competing interest statement for this submission. On behalf of all authors, disclose any competing interests that could be perceived to bias this work—acknowledging all financial support and any other relevant financial or non-financial competing interests.<br><br>This statement **will appear in the published article** if the submission is accepted. Please make sure it is accurate. View published research articles from *PLOS ONE* for specific examples. | The authors have declared that no competing interests exist. |

| | |
|---|---|
| **NO authors have competing interests**<br><br>Enter: *The authors have declared that no competing interests exist.*<br><br>**Authors with competing interests**<br><br>Enter competing interest details beginning with this statement:<br><br>*I have read the journal's policy and the authors of this manuscript have the following competing interests: [insert competing interests here]*<br><br>* typeset | |
| **Ethics Statement**<br><br>Enter an ethics statement for this submission. This statement is required if the study involved:<br><br>• Human participants<br>• Human specimens or tissue<br>• Vertebrate animals or cephalopods<br>• Vertebrate embryos or tissues<br>• Field research<br><br>Write "N/A" if the submission does not require an ethics statement.<br><br>General guidance is provided below. Consult the submission guidelines for detailed instructions. **Make sure that all information entered here is included in the Methods section of the manuscript.** | No ethics approval required for the project. |

*Powered by Editorial Manager® and ProduXion Manager® from Aries Systems Corporation*

| | |
|---|---|
| **Format for specific study types**<br><br>**Human Subject Research (involving human participants and/or tissue)**<br>• Give the name of the institutional review board or ethics committee that approved the study<br>• Include the approval number and/or a statement indicating approval of this research<br>• Indicate the form of consent obtained (written/oral) or the reason that consent was not obtained (e.g. the data were analyzed anonymously)<br><br>**Animal Research (involving vertebrate animals, embryos or tissues)**<br>• Provide the name of the Institutional Animal Care and Use Committee (IACUC) or other relevant ethics board that reviewed the study protocol, and indicate whether they approved this research or granted a formal waiver of ethical approval<br>• Include an approval number if one was obtained<br>• If the study involved *non-human primates*, add *additional details* about animal welfare and steps taken to ameliorate suffering<br>• If anesthesia, euthanasia, or any kind of animal sacrifice is part of the study, include briefly which substances and/or methods were applied<br><br>**Field Research**<br><br>Include the following details if this study involves the collection of plant, animal, or other materials from a natural setting:<br>• Field permit number<br>• Name of the institution or relevant body that granted permission | |
| **Data Availability**<br><br>Authors are required to make all data underlying the findings described fully available, without restriction, and from the time of publication. PLOS allows rare exceptions to address legal and ethical concerns. See the PLOS Data Policy and FAQ for detailed information. | Yes - all data are fully available without restriction |

*Powered by Editorial Manager® and ProduXion Manager® from Aries Systems Corporation*

| | |
|---|---|
| A Data Availability Statement describing where the data can be found is required at submission. Your answers to this question constitute the Data Availability Statement and **will be published in the article**, if accepted.<br><br>**Important:** Stating 'data available on request from the author' is not sufficient. If your data are only available upon request, select 'No' for the first question and explain your exceptional situation in the text box.<br><br>Do the authors confirm that all data underlying the findings described in their manuscript are fully available without restriction? | |
| **Describe where the data may be found in full sentences. If you are copying our sample text, replace any instances of XXX with the appropriate details.**<br><br>• If the data are **held or will be held in a public repository**, include URLs, accession numbers or DOIs. If this information will only be available after acceptance, indicate this by ticking the box below. For example: *All XXX files are available from the XXX database (accession number(s) XXX, XXX.).*<br>• If the data are all contained **within the manuscript and/or Supporting Information files**, enter the following: *All relevant data are within the manuscript and its Supporting Information files.*<br>• If neither of these applies but you are able to provide **details of access elsewhere**, with or without limitations, please do so. For example:<br><br>*Data cannot be shared publicly because of [XXX]. Data are available from the XXX Institutional Data Access / Ethics Committee (contact via XXX) for researchers who meet the criteria for access to confidential data.*<br><br>*The data underlying the results presented in the study are available from (include the name of the third party* | All relevant data are within the manuscript |

Powered by Editorial Manager® and ProduXion Manager® from Aries Systems Corporation

| | |
|---|---|
| *and contact information or URL).*<br>• This text is appropriate if the data are owned by a third party and authors do not have permission to share the data.<br><br>* typeset | |
| Additional data availability information: | |

Manuscript

Click here to access/download;Manuscript;Thu Nguyen_manuscript.docx

# Trends in vegetation dynamics associated with urban development: The role of golf courses

**Thu Thi Nguyen[1,3*], Paul Barber[2,3], Richard Harper[3], Tran Vu Khanh Linh[4], Bernard Dell[3]**

**1** Vietnam National University of Forestry (VNUF), Xuan Mai, Chuong My, Ha Noi, Vietnam,
**2** ArborCarbon Pty Ltd, 1 City Farm Place, East Perth, WA 6004, Australia,
**3** Agricultural and Forestry Sciences, Murdoch University, Murdoch, WA 6150, Australia,
**4** Faculty of Forestry, Nong Lam University - Ho Chi Minh City, Thu Duc District, Ho Chi Minh City, Vietnam.
*Corresponding author
Emails: Thu.Nguyen@murdoch.edu.au; thu.nguyen.2k14@gmail.com

## Abstract

Globally, cities have grown rapidly in size and density and this has caused profound impacts on urban forest ecosystems. Urbanization requiring clearing of vegetation reduces ecosystem services that benefit both city dwellers and biodiversity. Understanding spatial and temporal patterns of vegetation changes associated with urbanization is vital for future sustainable urban development. We used Landsat time series data for the period 1988 to 2018 to characterize changes in vegetation cover and habitat connectivity in the Perth Metropolitan Area, a rapidly urbanising biodiversity hotspot, as a case study to understand the impacts of urbanization on urban forests. Moreover, as golf courses are rapidly increasing in many urban areas, we assessed the role of golf courses in maintaining vegetation cover and creating habitat connectivity. We employed (1) land use classification, post-classification change detection, and (2) Morphological Spatial Pattern Analysis (MSPA). Over 17,000 hectares of vegetation were cleared and the area of vegetation contributing to biodiversity connectivity was reduced significantly over the three decades. The spatial patterns of vegetation loss and gain were different in each of the three decades reflecting the implementation of urban planning. Furthermore, MSPA analysis showed that the reduction in vegetation cover led to habitat fragmentation with a significant decrease in the core and bridge classes and an increase in isolated patches in the urban landscape. Golf courses played a useful role in maintaining vegetation cover and contributing to connectivity in a regional biodiversity hotspot. Our findings suggest that urban planning needs to more carefully consider land clearing and its impacts on connectivity in the landscape. Moreover, there is a need to take into consideration opportunities for off-reserve conservation in smaller habitat fragments such as in golf courses in sustainable urban management.

# Introduction

Globally, cities have grown rapidly in number and size over recent decades [1, 2]. This trend is predicted to continue as urban areas are expected to absorb most of the global population growth [3] . While the process of urbanization presents key implications for changes in physical landscapes and demographic characteristics, it can cause profound impacts on environmental components, especially on urban forest ecosystems [4, 5].

Vegetation in urban landscapes is critically important because it can provide goods and services, and full ecosystem functions that benefit city dwellers and the environment. On the one hand, a remarkable range of human well-being benefits are delivered from urban green spaces including mitigating the urban heat island (UHI) effect which is a threat to human health [6, 7]; reducing stress [8], risk of poor mental health [9] and mortality from cardiovascular disease [10]; and improving healing times [11], self-esteem and empowerment [12], and cognitive ability [13]. On the other hand, urban green spaces provide various ecosystem services such as strengthening resistance to natural disasters [14], promoting biological processes such as pollination [15], and reducing surface erosion from stormwater runoff [16]. The amount of vegetation in cities strongly influences biodiversity, especially where vegetation is set aside during the process of urbanization [17]. However, if species dispersal and exchange among these patches is insufficient to allow gene flow and diversity, loss of regional biodiversity is inevitable [18]. Therefore, urban development that requires clearing of vegetation causes reduction in vegetation cover, habitat loss and fragmentation, and hence may be considered a threat to biodiversity [17, 19].

Urban conservation strategies must therefore consider not only the size and quality of habitat reserves, but the connectivity in the intervening urban matrix [19]. While the need to protect large habitat reserves is obvious, opportunities for off-reserve conservation of smaller habitat fragments should not be overlooked [20]. Understanding spatial and temporal patterns of vegetation change associated with urbanization, as well as opportunities for the preservation of green spaces outside natural reserves, is vital for future sustainable urban development especially in areas of global ecological importance.

As the number of golf courses is rapidly increasing in many urban areas worldwide [21], there have been many environmental arguments about this green space category in urban landscapes.

Golf courses are considered to be major polluters of the environment through pesticide and fertilizer use [22]. However, golf courses in urban settings create large green-area habitats, which even surpass many nature reserves in size [23]. Potentially, urban golf courses could become more purposeful areas for biodiversity conservation and the provision of ecosystem services in cities [24]. Previous studies have identified some of the biological values of golf courses, such as providing refuge habitats for urban-avoiding wildlife [23, 25, 26]. Nevertheless, little research has been undertaken to comprehensively assess the role of golf courses in maintaining vegetation patches as interconnected nodes in urban landscapes during a long period of urban development where vegetation clearing was significant.

Vegetation clearing for urban expansion can occur gradually over multiple years. Satellite-based remote sensing holds certain advantages in the characterization of these changes in urban landscapes because of the large spatial coverage, high time resolution, and wide availability of data [27]. Landsat images of medium-resolution allow for mapping urban areas at a large spatial scale [28]. Many methods have been used to detect, monitor and quantify vegetation changes, but differences in vegetation index and land use classification are the most widely used methods for vegetation change over a long period of time [28]. A great number of vegetation indices have been proposed, ranging from very simple to very complex band combinations [29]. The most widely-used vegetation index is the Normalized Difference Vegetation Index (NDVI) [29] which separates green vegetation from other surfaces based on the ability of chlorophyll to absorb red light for photosynthesis and reflect the near-infrared (NIR) wavelengths . Furthermore, information on vegetation cover dynamics can be combined with Morphological Spatial Pattern Analysis (MSPA) to describe the spatial configuration of the ecosystem at the pixel level, making it possible to detect temporal changes in the structural connectivity of habitats in urban settings [30].

Therefore, to enhance the understanding of vegetation dynamics associated with urbanization and the role of golf courses in maintaining urban forests, we used Landsat imagery to map vegetation cover and to assess its spatial and temporal distribution over three decades from 1988 to 2018. Then, maps of vegetation cover were used for MSPA analysis to detect changes in habitat connectivity. We chose the Perth Metropolitan area for the study as it lies within a rapidly urbanizing biodiversity hotspot in Australia. The three primary research objectives

were to: (1) determine the spatial and temporal patterns of vegetation clearing in urbanization; (2) evaluate the spatial and temporal patterns of green landscape connectivity; and (3) assess the impact of golf courses on preserving green spaces in an urban landscape. This analysis will provide a useful perspective on the land-use pressure facing vegetation remnants in the region and their connectivity in the Southwest Australia Ecoregion (SWAE) which is recognised as one of 35 international biodiversity hotspots, and is home to over 1500 plant species with a high degree of endemism [31].

# Methods

## Study area

The study belongs to the Perth Metropolitan area covering four sub-regions (North West, Middle Central, Inner Central and South West) (Fig 1). Perth is located in a globally recognised biodiversity hotspot [32] in a region of Mediterranean-type climate and has experienced extensive urban development at an unsustainable rate [33]. Together with urbanization, the golf industry has expanded contributing to a growing proportion of Perth's urban green space. There are 34 golf courses in the study area (Fig 1)

**Figure 1. Study area**

## Approaches

We conducted two types of analyses in this study: (1) assessment of vegetation cover change in golf courses since 1988 relative to surrounding areas to provide a broad overall context of the urban vegetation changes that have taken place throughout the region and the role of golf courses; and (2) assessment of MSPA. In these analyses, we used four data sets (1988, 1998, 2008, 2018) covering thirty years of urban development. The steps taken in this study are summarized in a flow chart (Fig 2).

### Landsat data and pre-processing

Three Landsat 5 Thematic Mapper images (WRS path 113 row 82) were acquired from 1988 to 2008 and one Landsat OLI 8 (WRS path 113 row 82) was acquired in 2018 at four time steps (1988, 1998, 2008, 2018). The Landsat imagery were obtained from the US Geological Survey (USGS) Earth Resources Observation. Image dates were

selected from cloud-free scenes acquired during December (summer, dry season). Georeferencing was performed at the USGS prior to downloading the data (L1T level of systematic geometric accuracy) and no further refinement was undertaken. Atmospheric and topographic corrections were performed on the Landsat data sets. The atmospheric correction was carried out to adjust the multitemporal dataset to a common radiometric scale [34].

The first process of atmospheric correction was conversion of the digital number (DN) remote sensing data values to at-sensor radiance based on the image header file. After that we employed the image-based models - dark object subtraction (DOS) to correct atmospheric scattering scene-by-scene [35, 36]. Topographic correction was conducted to remove the topographic effects. We used sun-canopy-sensor (SCS) correction based on the 30 m digital elevation model (DEM) because topographic shading is not only due to slope but also to shadowing of one tree crown over another and this is one of the most widely and effective used methods of topographic correction [37].

**Figure 2. Flow chart summarizing the major steps taken during the investigation.**

(NDVI: Normalized Difference Vegetation Index; MSPA: Morphological Spatial Pattern Analysis)

## Classification

In a preliminary step, we used a decor relation stretch to enhance the image for more effective visualization. Prior to image classification, NDVI images were generated. A classification technique was then applied to the NDVI images of 1988, 1998, 2008 and 2018 using Arc-GIS 10.3 software. NDVI images were obtained by calculating the ratio between the visible (VIS) and near-infrared (NIR) bands of the satellite image by equation 1:

$$\text{NDVI} = \frac{(\text{NIR}-\text{VIS})}{(\text{NIR}+\text{VIS})} \qquad (1)$$

Landsat TM data of different dates were independently classified based on the NDVI values. The water bodies have negative NDVI values, whereas, bare soil and built-up areas have an NDVI value of around zero. Green vegetation, on the other hand, has

stronger near-infrared reflectance thereby providing moderate and high NDVI values close to +1 [38]. Based on this information, the four NDVI images were classified into three classes by using the NDVI threshold ranges technique in Arc-GIS 10.3 software.

The classification based on NDVI threshold was evaluated using accuracy assessment. An error matrix compared information from a classified image or land cover map to known reference (truth) sites for a number of sample points assessed in 2018. We obtained photographs of representative land use categories with GPS locations to assist us in our image interpretations. Also, we used Google Earth images, true and false colour combination images and knowledge-based information including expert knowledge, land use maps and reports. For historical images, Google Earth was used to substitute the traditional reference data collection on each of the sites [39]. Based on data of accuracy assessment, we reclassified the preliminary land use classification maps to improve the accuracy of classification.

## Vegetation change detection

In order to detect the vegetation cover change, we created binary maps of vegetation and non-vegetation from the classified maps in the previous analysis, one for each adjacent pair of time steps, which depict where degradation occurred within a decade of urbanization. This post-classification analysis uses two images from different dates and classifies them independently. We then calculated changes in vegetation cover type using equation 2:

$$\text{Change area} = D_2 - D_1 \qquad (2)$$

where $D_1$ and $D_2$ are the area of the target vegetation cover at the beginning and the end of the study period, respectively. This analysis allows the calculation of vegetation loss and gain in each period.

## Golf courses Analysis 1

We compared the vegetation cover, and the change detection (vegetation loss and gain) taking place within all golf courses in the study area, and in their surrounding regions. After creating the GIS boundaries of these golf courses, we extracted the vegetation cover and the vegetation change within these boundaries at four time steps in 1988,

1998, 2008 and 2018 to compare vegetation dynamic within the golf courses and the whole study area over time.

## MSPA analysis for the structural connectivity of habitats

MSPA was employed to describe the structural connectivity of habitats in Perth for four time steps in 1988, 1998, 2008 and 2018. This method describes the spatial and temporal configuration of the ecosystem at the pixel level [40], which was based on the concept of "habitat availability" and "graphic theory" [41, 42] in which the landscape is considered as a collection of nodes, and links with a node is a place where connectivity exists and will depend on the width of itself. The output of the MSPA analysis includes the seven structural categories into which habitats are divided, including core, edge, perforation, bridge, loop, branch and islet (Table 1).

**Table 1: Definition of MSPA classes (see [43] and [44]).**

| Class | Description | Ecological Meaning |
|---|---|---|
| **Core** | Foreground surrounded by foreground and greater than the user-specified edge width from background | Large-scale natural patches with high connectivity |
| **Edge** | Foreground that separates core from background | The transition zone between green space and non-green space. |
| **Perforation** | Foreground that separates core from interior areas of background | Unnatural patch inside the core area. |
| **Bridge** | Linearly oriented foreground that connects two disjunct core areas | Striped ecological land between core areas with high connectivity, which is equivalent to the connecting corridor of the green space network. |
| **Loop** | Linearly oriented foreground that extends from core and connects back to the same core area (e.g. a handle) | Connecting corridor inside a large natural patch. |
| **Branch** | Linearly oriented foreground that extends from core and terminates in background | Striped ecological land with low connectivity. |
| **Islet** | Area of foreground that is too small to contain core | Small natural patches that are not connected to each other. |

In order to undertake the MSPA analysis, we defined the input data (foreground class). For this study, we used the classified maps for 1988, 1998, 2008, 2018 in analysis 1 to create the binary maps which contained vegetation and non-vegetation classes. Hence, the high and full covered vegetation pixels were defined as the foreground pixels (green landscape) in the MSPA approach. The results of MSPA analysis for the four time steps allowed us to assess the changes in habitat connectivity in the major urban area of Perth over 30 years of urbanization.

### Golf course analysis 2

To assess the role of golf courses in maintaining biodiversity connectivity over 30 years, we compared the habitat connectivity taking place within all golf courses in the study area and in their surrounding regions. Using the GIS boundaries of the golf courses, we extracted the habitat connectivity within the golf course boundaries for four time steps (1988, 1998, 2008 and 2018).

# Results

## Land use classification in Perth

Data sets representing four time periods (1988, 1998, 2008 and 2018) are shown in Figure 3 and Table 2, which provides an overview of the land use changes (vegetation, built up and bare land, water bodies) over recent decades. From the 1988 and 1998 data sets, it is evident that over half of the region was vegetated. However, the urban footprint of built up and bare land area had increased 10% by 2018. As a consequence, there was a significant decrease in vegetation cover, which comprised 56% of the land surface in 1988 and declined by 10.1% over the next 30 years (Table 2). We obtained an overall accuracy (OA) of classification of 87% and a kappa coefficient of 91% for the three classes.

**Table 2. Land use classification within the Perth Metropolitan Region.**

| Land use category | | Whole area | | | Golf course | | |
|---|---|---|---|---|---|---|---|
| | | Vegetation | Built up and bare soil | Water bodies | Vegetation | Built up and bare soil | Water bodies |
| **1988** | Area (ha) | 98,446 | 74,376 | 2,667 | 929 | 210 | 0.1 |
| | Proportion (%) | 56.1 | 42.4 | 1.5 | 81.5 | 18.5 | 0.0 |
| **1998** | Area (ha) | 91,754 | 81,464 | 2,271 | 1,042 | 97 | 0.7 |
| | Proportion (%) | 52.3 | 46.4 | 1.3 | 91.4 | 8.5 | 0.1 |
| **2008** | Area (ha) | 88,341 | 84,971 | 2,176 | 1,084 | 55 | 0.2 |
| | Proportion (%) | 50.3 | 48.4 | 1.2 | 95.1 | 4.9 | 0.0 |
| **2018** | Area (ha) | 80,755 | 92,243 | 2,491 | 1,093 | 46 | 0.7 |
| | Proportion (%) | 46.0 | 52.6 | 1.4 | 95.9 | 4 | 0.1 |

**Figure 3. Map of land use classification in four time steps.**

Using the GIS layer of land use categories, we extracted the data for three classes within the golf courses in comparison with the whole area (Table 2). Our analysis shows that while there was a significant decrease in vegetation cover throughout the region, the total area of golf courses remained unchanged at 1140 ha over the last 30 years.

## Spatial patterns of vegetation change

To characterize spatial patterns of vegetation dynamics, we detected the vegetation loss and gain for each of the three decades (Fig 4). In the period 1988 to 1998, vegetation clearing occurred intensively in the central and north regions. From 1998 to 2008, vegetation loss expanded to the north and south of the city. However, in the last decade from 2008 to 2018, vegetation loss accelerated in the distal regions with urbanization. However, there was also some vegetation gain over the three decades (Fig 4, Table 3) predominantly in the north part of the city.

**Figure 4. Change in vegetation over three decades.**

**Table 3. Vegetation loss and gain (ha) in three time periods over the period 1988 to 2018.**

| Period | | 1988-1998 | 1998-2008 | 2008-2018 |
|---|---|---|---|---|
| **Whole Area** | Vegetation loss | 22,231 | 19,250 | 20,178 |
| | Vegetation gain | 15,538 | 15,838 | 12,591 |
| | Net loss | 6,692 | 3,412 | 7,587 |
| **Golf courses** | Vegetation loss | 68 | 27 | 40 |
| | Vegetation gain | 219 | 92 | 43 |
| | Net gain | 152 | 64 | 4 |

Calculation of changes within the golf courses and in the whole area (Table 3) showed that the major urban area of Perth experienced a net loss in vegetation cover. Though vegetation compensation occurred together with vegetation clearing over 30 years of urbanization, the vegetation loss was always much larger than vegetation gain with the largest net vegetation loss occurring in the last decade. However, the golf courses showed a different trend where the net gain of vegetation cover happened over three decades and the largest gain was between 1988 and 1998.

## Analysis of connectivity components of green space networks

Results of the MSPA analysis indicate that the reduction in vegetation cover over the last thirty years has led to a decline in connectivity. Among the two types of landscape that are important for connectivity (core and bridge), the total area of core class decreased by about 10% over three decades while the bridge class was maintained at around 37,000 ha, but the proportion of this class per total vegetation cover area (VCA) increased due to the reduction of vegetation cover over time (Table 4).This analysis also shows the fluctuation in the areas of the rest of the MSPA classes including islets, loops, edges, perforations, and branches which do not contribute to connectivity in the landscape. The proportion of these classes increased through time from 24% in 1988 to 30% in 2008 and 2018.

Figure 5 shows that the core area was distributed mostly in the north part of the city and their areas decreased significantly in later years. In 1988, the bridge class covered a large area of the city central region but this decreased over time. In recent years, most

of the vegetation cover in the central region of the city belongs to the islet, loop, edge, perforation and branch classes, illustrating that isolation became more serious in the central region of the city over the three decades.

**Figure 5. Results of MSPA analysis.**

The vegetation cover within golf courses also contributes to connectivity. The proportion of core area within golf courses fluctuated between 12% and 27%. Moreover, the largest proportion of vegetation in golf courses was classified as bridge but it experienced a downward trend from 52% to 41% in three decades. Of the remaining classes which do not contribute to connectivity, the edge and loop classes accounted for a higher proportion with each of them contributing 7% to 16% of total vegetation cover.

**Table 4. Results of MSPA analysis of connectivity of Perth's vegetation from 1988 to 2018.**

| Landscape type | Year | Perth Metropolitan Region | | Within golf courses | |
|---|---|---|---|---|---|
| | | Area (ha) | Proportion of total VCA (%) | Area (ha) | Proportion of total VCA (%) |
| **Core** | 1988 | 32,012 | 32.6 | 325 | 23.2 |
| | 1998 | 20,607 | 22.5 | 171 | 12.7 |
| | 2008 | 22,251 | 25.3 | 230 | 16.7 |
| | 2018 | 18,101 | 22.5 | 383 | 27.3 |
| **Bridge** | 1988 | 37,127 | 37.9 | 738 | 52.6 |
| | 1998 | 39,412 | 43.0 | 814 | 60.2 |
| | 2008 | 37,902 | 43.1 | 732 | 53.1 |
| | 2018 | 37,503 | 46.7 | 588 | 42.0 |
| **Islet** | 1988 | 10,396 | 10.6 | 31 | 2.2 |
| | 1998 | 16,436 | 17.9 | 78 | 5.8 |
| | 2008 | 12,922 | 14.7 | 15 | 1.1 |
| | 2018 | 12,349 | 15.4 | 37 | 2.7 |
| **Perforation** | 1988 | 1,099 | 1.1 | 3 | 0.2 |
| | 1998 | 486 | 0.5 | - | - |
| | 2008 | 834 | 1.0 | - | - |
| | 2018 | 406 | 0.5 | 6 | 0.4 |
| **Edge** | 1988 | 7,430 | 7.6 | 162 | 11.5 |
| | 1998 | 4,814 | 5.3 | 95 | 7.0 |
| | 2008 | 4,986 | 5.7 | 147 | 10.7 |
| | 2018 | 5,218 | 6.5 | 230 | 16.4 |
| **Loop** | 1988 | 5,670 | 5.8 | 112 | 8.0 |
| | 1998 | 6,295 | 6.9 | 146 | 10.8 |
| | 2008 | 5,607 | 6.4 | 209 | 15.2 |
| | 2018 | 3,669 | 4.6 | 129 | 9.2 |
| **Branch** | 1988 | 4,361 | 4.5 | 32 | 2.3 |
| | 1998 | 3,619 | 4.0 | 47 | 3.5 |
| | 2008 | 3,491 | 4.0 | 43 | 3.2 |
| | 2018 | 3,117 | 3.9 | 29 | 2.1 |

# Discussion

We used Landsat imagery to characterize the patterns of vegetation change, habitat connectivity and the role of golf courses in maintaining green spaces and connectivity in an urban landscape. We found that vegetation clearing led to reduction in habitat connectivity in the Perth Metropolitan Region. However, golf courses can play an important role in maintaining vegetation and supporting biodiversity connectivity in urban landscapes. Prior work has documented the increase in the urban footprint of Perth using multi-temporal urban expansion statistics derived from Satellite imagery [45]. However, this study did not address the issues of vegetation dynamics, nor the biodiversity and structure of the vegetation and habitat connectivity.

## Vegetation clearing and urbanization

The vegetation dynamics in the major urban areas of Perth reflect the pattern of urbanization over time. The reduction in green space found in this study can be explained as a close relation to the process of development in this city. Over the last three decades, urban development in Perth has taken place at an unsustainable rate [33]. In the early 1990s to 2006, Perth's population grew by around 1.8%, but the figure has nearly doubled since then [46]. Also, this study indicated that, from 1988 to 2018, Perth's urban footprint increased from 74 to 92 ha (Table 2) and is consistent with previous research in urban growth in this region [45]. In addition to expansion, the city has become denser due to the construction of new residential dwellings in urbanised areas. In the last 20 years, on average, 740 ha/yr of urban and urban deferred zoned land was consumed by subdivision, and 830 ha/yr was consumed by construction in the Perth metropolitan area and nearby Peel region [47].

The difference in spatial patterns of vegetation loss are also related to the urban plans of this city. Our results show that between 1988 and 1998, there was significant vegetation loss in the central region of the city which can be linked to The Corridor Plan [48]. Historically, Perth's development pattern from 1970s to 1990s was based on linear corridors stretching out from the city's core, with large non-urban wedges between each of these corridors [48]. However, from 1998 to 2008 and from 2008 to 2018, vegetation

clearing was more significant in outer subregions of the north-west and south-west of the city due to the adoption of METROPLAN [49]. Perth recently has been divided into subregional areas, rather than corridors for planning purposes. The two coastal subregions (the North-West and South-West) included in our study have consistently achieved higher rates of population growth under Metroplan [50].

Originally the region was covered by woodlands dominated by eucalypts and banksias and coastal heath interspersed with chains of wetlands. Perth is home to a rich biodiversity, with more than 1,700 species of flowering plants and iconic species of threatened fauna in the region [51]. Therefore, such vegetation clearing for urbanization has resulted in the devastating loss of significant natural habitats in this biodiversity hotspot city, leading to the designation of an endangered ecological community by the Australian Government [32].

## Connectivity of green space networks in urban landscapes

The MSPA analysis indicates that the loss of area of vegetation as a consequence of urban expansion has led to a marked reduction in connectivity of green space networks in the Perth urban landscape. In the MSPA analysis, only cores and bridges can contribute to the connectivity between the habitat areas in the landscape. The reduction of these areas in our study indicates the high impact of urbanization on habitat connectivity through the loss of core areas, which act as stepping stones between forest habitat patches, and the loss of bridges that act as structural corridors to link two or more core areas [52]. This analysis also shows the increase in proportion of islets, which are totally isolated patches, and other classes (perforations, loops, branches, edges) that cannot reach a new core habitat area for originating the potential movement [52]. Clearly, expansion of Perth city has fragmented the remaining blocks of natural habitat and increased isolation of natural habitats. This may reduce population and gene flow among patches and may disrupt the connection between subpopulations and a large regional population [53].

The fragmentation process was obvious in the central region of the city and in recent decades it has become more serious in the outer parts of the city. This is critical as Perth is within a globally recognised biodiversity hotspot, which is home to rich biodiversity

found nowhere else in the world. The connectivity in the major urban area of Perth is not only critical for the linkage of habitats within the Swan Coastal Plain but also for the connection of these coastal habitats to a large regional biosphere in south-western Australia.

The results also illustrated that a ‘Core’ area and a network of ‘Bridge’ types exist in the central and outer subregions of Perth. This is the consequence of early conservation efforts of the government which created protected areas such as Kings Park, Bold Park and other significant areas. Very few cities in the world have such large areas of natural bushland in the centre of a big city [54]. However, future urban growth will continue to put pressure on the biodiversity. If current policies (Perth and Peel@3.5million) are fully implemented, existing stocks of urban and urban deferred land would be consumed by about 2075 [55]. The challenge for urban planning to preserve urban forests and biodiversity is thus increasing.

Our MSPA output with spatial distribution of seven classes provides fundamental information for future urban planning. There is a need to maintain important green spaces which are classified as cores and bridges in the city, especially in the central region where most of the natural vegetation exists as islands. Also, the MSPA branch classes can be used to identify candidate ecological restoration areas. The branch class can be thought of [56] as a foundation of a potential corridor that could, if revegetated, connect two spatially disjunct core areas  to improve connectivity in the larger region.

Although other analyses, such as functional connectivity, should be taken into account in landscape connectivity assessment [40], the structural connectivity analysis in this study will be useful for determining the priority protection level and critical areas of the connecting corridor, informing conservation strategies at a variety of scales, especially when the biodiversity values of this region are suffering from various threats including land clearing, feral animals, weed incursions, more frequent fires through arson and tree disease [51].

## The role of golf courses in maintaining urban forest

Our study indicates that golf courses account for a significant proportion of the urban area of Perth. This category of land use has been vital in maintaining green space in

urban areas over the past thirty years. In contrast to the overall decline in urban green space, golf courses have preserved green spaces within urban settings and even created a net gain of vegetation cover over time. The highest net gain was seen in the period between 1988 to 1998 when some golf courses were established resulting in the planting of trees.

In the green 'matrix' of Perth, golf courses with their significant area of vegetation cover have contributed considerably to the connectivity in the urban landscape. A significant proportion of their green space was classified as core or bridge categories. The proportion of vegetation within golf courses classified as bridges was higher than in the whole study area. Golf courses with large areas of native vegetation provide "links" to other large natural patches of urban vegetation.

Although there are concerns with the environmentally negative impacts of golf courses as a source of pollution through pesticide and fertiliser usage [22], habitat modification [25] and high water usage [57], previous studies provide evidence about the biological values of golf courses, such as providing refugial habitat for urban-avoiding wildlife [24, 58-66]. Our study indicates that golf courses in urban settings have been maintaining large green-area habitats and played an important role in biodiversity connectivity in the city.

## Monitoring urban forest dynamics

In this study, we utilized medium-resolution satellite remote sensing data to identify land use classes, characterise vegetation dynamics and connectivity. The data maps the spatial and temporal patterns of land use types characterizing a consistent, detailed vegetation dynamic of the city [45, 67]. Clearly, the biophysical elements of urban landscapes are well-reflected through physical features (NDVI) derived from remote sensing data with an accuracy of up to 89%.

Despite these kinds of data, it is hard to describe the detailed information of ecosystem such as species composition and forest structure. However, they show their advantage in mapping land cover dynamics across large areas of big cities over time when high resolution imagery is not available. For monitoring urban forests in big cities, the large scale and long temporal datasets are more advantageous compared with datasets that

focus only on discerning a specific land use type in a relatively small area [27, 68, 69]. This is because it allows spatially detailed identification of changes associated with development over time. Therefore, this method provides baseline information for sustainable urban planning and development. In addition, the MSPA analysis can further evaluate the dynamic of vegetation cover by the describing the spatial configuration of ecosystems at the pixel level, detecting changes of habitat connectivity over time [40].

# Conclusions

With rapid urban expansion, the most meaningful question to address is how to balance urban development and urban forest preservation. Urbanization requiring vegetation clearing is inevitable in many cities worldwide. Our study found a significant loss of vegetation cover in a biodiversity hotspot over three decades of urbanization, which led to a reduction in habitat connectivity in the urban landscape. A lesson learned from the experience of urbanization in Perth is that any future urban growth following the patterns observed over the past three decades will continue to put pressure on maintaining urban forest ecosystem and biodiversity conservation. As cities continue to grow in response to socio-economic development, considering all opportunities for urban biodiversity conservation is important. Urban conservation strategies must therefore consider not only the protected areas, but also the off-reserve sites.

Our study indicates that golf courses in urban settings have been maintaining green-area habitats and have played an important role in biodiversity connectivity in the city. Potentially, urban golf courses could become more purposefully managed for biodiversity conservation and the improvement of critical ecosystem services in urban areas. In the rapidly urbanizing biodiversity hotspots like Perth, where fragmentation is one the biggest threats to biodiversity, the way that golf courses contribute to increase the connectivity in the intervening urban matrix should not be underestimated. Therefore, it is important for government authorities and golf courses owners to pay more attention in maintaining ecosystem health in urban golf courses.

# Acknowledgments

The authors thank Dr. Harry Eslick for his valuable advice in helping to design the research and Mr. Pham Dang Manh Hong Luan for his advice in data analysis. The research was supported by the Vietnamese Ministry of Education and Training and Murdoch University through a VIED-MU joint PhD scholarship to the first author.


# References


1. Tabea TaE, Knop. A landscape ecology approach identifies important drivers of urban biodiversity. Global Change Biol. 2015;21 (4):1652-67.
2. Bagan H, Yamagata Y. Land-cover change analysis in 50 global cities by using a combination of Landsat data and analysis of grid cells. Environmental Research Letters. 2014;9(6):064015. doi: 10.1088/1748-9326/9/6/064015.
3. UNDESA UNDoEaSA. World urbanization prospects: The 2011 revision. United Nations Department of Economic and Social Affairs/Population Division, New York: 2012.
4. McGrane SJ. Impacts of urbanisation on hydrological and water quality dynamics, and urban water management: a review. Hydrological Sciences Journal. 2016;61(13):2295-311. doi: 10.1080/02626667.2015.1128084.
5. Gaston KJ, Ávila-Jiménez ML, Edmondson JL. REVIEW: Managing urban ecosystems for goods and services. Journal of Applied Ecology. 2013;50(4):830-40. doi: 10.1111/1365-2664.12087.
6. Declet-Barreto J, Brazel AJ, Martin CA, Chow WTL, Harlan SL. Creating the park cool island in an inner-city neighborhood: heat mitigation strategy for Phoenix, AZ. Urban Ecosystems. 2013;16(3):617-35. doi: 10.1007/s11252-012-0278-8.
7. Scott AA, Misiani H, Okoth J, Jordan A, Gohlke J, Ouma G, et al. Temperature and heat in informal settlements in Nairobi. PLOS ONE. 2017;12(11):e0187300. doi: 10.1371/journal.pone.0187300.
8. Van Den Berg AE, Custers MHG. Garndening promotes neuroendocrine and affective restoration from stress. Journal of Health Psychology. 2010;16(1):1359-053.
9. Mitchell R. Is physical activity in natural environments better for mental health than physical activity in other environments? Social Science & Medicine. 2013;91(Supplement C):130-4. doi: https://doi.org/10.1016/j.socscimed.2012.04.012.
10. Donovan GH, Butry DT, Michael YL, Prestemon JP, Liebhold AM, Gatziolis D, et al. The relationship between trees and human health: Evidence from the spread of the emerald ash borer. American Journal of Preventive Medicine. 2013;44(2):139-45. doi: https://doi.org/10.1016/j.amepre.2012.09.066.
11. Ulrich RS. View through a window may influence recovery from surgery. Science. 1984;224(4647):420.
12. Cecily Jane M. Promoting children's mental, emotional and social health through contact with nature: a model. Health Education. 2009;109(6):522-43. doi: 10.1108/09654280911001185.
13. Lee KE, Williams KJH, Sargent LD, Williams NSG, Johnson KA. 40-second green roof views sustain attention: The role of micro-breaks in attention restoration. Journal of Environmental Psychology. 2015;42(Supplement C):182-9. doi: https://doi.org/10.1016/j.jenvp.2015.04.003.
14. Kim G. Assessing urban forest structure, ecosystem services, and economic benefits on vacant land. Sustainability. 2016;8(7):679. doi: doi:10.3390/su8070679.
15. Zitkovic M. Managing green spaces for urban biodiversity. Boulevard Louis Schmidt 64 1040 Brussels, Belgium: Countdown 2010 Secretariat, IUCN Regional Office for Europe, 2008.

16. Seitz J, Escobedo F. Urban forests in Florida: Trees control stormwater runoff and improve water quality. University of Florida, Institute of Food and Agricultural Sciences. IFAS Extension Publication FOR184.2014.
17. Bailey D, Schmidt-Entling MH, Eberhart P, Herrmann JD, Hofer G, Kormann U, et al. Effects of habitat amount and isolation on biodiversity in fragmented traditional orchards. Journal of Applied Ecology. 2010;47(5):1003-13. doi: 10.1111/j.1365-2664.2010.01858.x.
18. Yu D, Liu Y, Xun B, Shao H. Measuring landscape connectivity in a urban area for biological conservation. CLEAN – Soil, Air, Water. 2015;43(4):605-13. doi: 10.1002/clen.201200448.
19. Bierwagen B. Connectivity in urbanizing landscapes: The importance of habitat configuration, urban area size, and dispersal. 2007;10:29-42. doi: 10.1007/s11252-006-0011-6.
20. Lindenmayer D, Franklin J. Conserving forest biodiversity: a comprehensive multiscaled approach2002.
21. Napton DE, Laingen CR. Expansion of Golf Courses in the United States. Geographical Review. 2008;98(1):24-41.
22. Guzmán C, & Fernández, D.,Javier Mesa. Environmental impacts by golf courses and strategies to minimize them: state of the art. International Journal of Arts & Sciences. 2014;7(3):403-17.
23. Colding J, Folke C. The role of golf courses in biodiversity conservation and ecosystem management. Ecosystems. 2009;12(2):191-206. doi: 10.1007/s10021-008-9217-1.
24. Hodgkison SC, Hero JM, Warnken J. The conservation value of suburban golf courses in a rapidly urbanising region of Australia. Landscape and Urban Planning. 2007;79(3):323-37. doi: https://doi.org/10.1016/j.landurbplan.2006.03.009.
25. Terman MR. Natural links: naturalistic golf courses as wildlife habitat. Landscape and Urban Planning. 1997;38(3):183-97. doi: https://doi.org/10.1016/S0169-2046(97)00033-9.
26. Puglis HJ, Boone MD. Effects of Terrestrial Buffer Zones on Amphibians on Golf Courses. PLOS ONE. 2012;7(6):e39590. doi: 10.1371/journal.pone.0039590.
27. Theobald DM. Development and Applications of a Comprehensive Land Use Classification and Map for the US. PLOS ONE. 2014;9(4):e94628. doi: 10.1371/journal.pone.0094628.
28. Hu T, Yang J, Li X, Gong P. Mapping urban land use by using landsat images and open social data. Remote Sensing. 2016;8(2):151. PubMed PMID: doi:10.3390/rs8020151.
29. Li X, Liu X, Gong P. Integrating ensemble-urban cellular automata model with an uncertainty map to improve the performance of a single model. International Journal of Geographical Information Science. 2015;29(5):762-85. doi: 10.1080/13658816.2014.997237.
30. Vogt P, Riitters K, Estreguil C, Kozak J, G. Wade T, Wickham J. Mapping spatial patterns with morphological image processing.2007. 171-7 p.
31. Myers N, Mittermeier RA, Mittermeier CG, da Fonseca GAB, Kent J. Biodiversity hotspots for conservation priorities. Nature. 2000;403(6772):853-8. doi: 10.1038/35002501.
32. Environment Dot. Shrublands and Woodlands of the eastern Swan Coastal Plain in Community and Species Profile and Threats Database. In: Environment Dot, editor. Canberra.2016.
33. Dhakal SP. Glimpses of sustainability in Perth, Western Australia: Capturing and communicating the adaptive capacity of an activist group. Consilience: The Journal of Sustainable Development. 2014;11(1):167–82.
34. Song C, Woodcock CE, Seto KC, Lenney MP, Macomber SA. Classification and Change Detection Using Landsat TM Data: When and How to Correct Atmospheric Effects? Remote Sensing of Environment. 2001;75(2):230-44. doi: https://doi.org/10.1016/S0034-4257(00)00169-3.
35. Cui L, Li G, Ren H, He L, Liao H, Ouyang N, et al. Assessment of atmospheric correction methods for historical Landsat TM images in the coastal zone: A case study in Jiangsu, China. European Journal of Remote Sensing. 2014;47(1):701-16. doi: 10.5721/EuJRS20144740.
36. Lu D, Mausel P, Brondizio E, Moran E. Assessment of atmospheric correction methods for Landsat TM data applicable to Amazon basin LBA research. International Journal of Remote Sensing. 2002;23(13):2651-71. doi: 10.1080/01431160110109642.

37. Gu D, Gillespie A. Topographic normalization of landsat TM images of forest based on subpixel sun–canopy–sensor geometry. Remote Sensing of Environment. 1998;64(2):166-75. doi: https://doi.org/10.1016/S0034-4257(97)00177-6.
38. Herring JWaD. Measuring Vegetation (NDVI & EVI) 2000. Available from: https://earthobservatory.nasa.gov/features/MeasuringVegetation.
39. Cha S-Y, Park C-H. The utilization of google earth images as reference data for the multitemporal land cover classification with MODIS data of North Korea.2007.
40. Vogt P, Riitters K, Estreguil C, Kozak J, G. Wade T, Wickham J. Mapping Spatial Patterns with Morphological Image Processing2007. 171-7 p.
41. Pascual-Hortal L, Saura S. Comparison and development of new graph-based landscape connectivity indices: towards the priorization of habitat patches and corridors for conservation. Landscape Ecology. 2006;21(7):959-67. doi: 10.1007/s10980-006-0013-z.
42. Saornil J, Gutiérrez J, Hernando A, Abril A, Sánchez B, Aparicio M. Structural connectivity as an indicator of species richness and landscape diversity in Castilla y León (Spain)2019. 286-97 p.
43. Wickham JD, Riitters KH, Wade TG, Vogt P. A national assessment of green infrastructure and change for the conterminous United States using morphological image processing. 2010.
44. Shi X, Qin M. Research on the Optimization of Regional Green Infrastructure Network. Sustainability. 2018;10(12):4649. PubMed PMID: doi:10.3390/su10124649.
45. MacLachlan A, Biggs E, Roberts G, Boruff B. Urban growth dynamics in Perth, Western Australia: Using applied remote sensing for sustainable future planning. Land. 2017;6(1):9. PubMed PMID: doi:10.3390/land6010009.
46. WorldPopulationReview. Perth Population 2019 2019 [21 February 2019]. Available from: http://worldpopulationreview.com/world-cities/perth-population/.
47. Commission WAP. Urban Growth Monitor Perth Methopolitan, Peel and Greater Bunbury Regions. In: WAPC WAPC, editor. Gordon Stephenson House 140 William Street Perth WA 6000: the Western Australian Planning Commission; 2019.
48. Authority MRP. The Corridor Plan for Perth. In: Authority MRP, editor. Perth, WA1970.
49. Development DoPaU. Metroplan: A planning strategy for the Perth Metropolitan Region. In: Development DoPaU, editor. Perth, WA.1990.
50. Bureau of Infrastructure TaREB. Population growth, jobs growth and commuting flows in Perth, . In: Transport DoIa, editor. Canberra ACT 2010.
51. Department of Biodiversity CaA. A methodology for the evaluation of wetlands on the Swan Coastal Plain, draft prepared by the Wetlands Section of the Department of Biodiversity. Perth, WA2017.
52. Saura S, Vogt P, Velázquez J, Hernando A, Tejera R. Key structural forest connectors can be identified by combining landscape spatial pattern and network analyses. Forest Ecology and Management. 2011;262(2):150-60. doi: https://doi.org/10.1016/j.foreco.2011.03.017.
53. Morjan CL, Rieseberg LH. How species evolve collectively: implications of gene flow and selection for the spread of advantageous alleles. Mol Ecol. 2004;13(6):1341-56. doi: 10.1111/j.1365-294X.2004.02164.x. PubMed PMID: 15140081.
54. Green T. Perth's Urban Forest: A WA 2.0 Project. 2018.
55. Commission WAP. Perth and Peel @ 3.5 Million. In: Department of Planning GoWA, editor. Perth,WA, Australia2015.
56. Wickham J, Riitters K, Vogt P, Costanza J, Neale A. An inventory of continental U.S. terrestrial candidate ecological restoration areas based on landscape context2017.
57. Neylan J. Improving The Environmental Management of New South Wales Golf courses. In: NSW DoEaCC, editor. 59–61 Goulburn Street, Sydney, PO Box A290.
58. Deslauriers MR, Asgary A, Nazarnia N, Jaeger JAG. Implementing the connectivity of natural areas in cities as an indicator in the City Biodiversity Index (CBI). Ecological Indicators. 2017. doi: https://doi.org/10.1016/j.ecolind.2017.02.028.

59. Dobbs EK, Potter DA. Naturalized habitat on golf courses: source or sink for natural enemies and conservation biological control? Urban Ecosystems. 2016;19(2):899-914. doi: 10.1007/s11252-015-0521-1.
60. Saarikivi J, Tähtinen S, Malmberg S, Kotze DJ. Converting land into golf courses – effects on ground beetles (Coleoptera, Carabidae). Insect Conservation and Diversity. 2015;8(3):247-51. doi: 10.1111/icad.12103.
61. Chester ET, Robson BJ. Anthropogenic refuges for freshwater biodiversity: Their ecological characteristics and management. Biological Conservation. 2013;166(Supplement C):64-75. doi: https://doi.org/10.1016/j.biocon.2013.06.016.
62. Saarikivi J, Idström L, Venn S, Niemelä J, Kotze DJ. Carabid beetle assemblages associated with urban golf courses in the greater Helsinki area. European Journal of Entomology. 2010;107(4):553-61. PubMed PMID: 814377100.
63. Hudson M-AR, Bird DM. Recommendations for design and management of golf courses and green spaces based on surveys of breeding bird communities in Montreal. Landscape and Urban Planning. 2009;92(3):335-46. doi: https://doi.org/10.1016/j.landurbplan.2009.05.017.
64. Colding J, Lundberg J, Lundberg S, Andersson E. Golf courses and wetland fauna. Ecological Applications. 2009;19(6):1481-91. doi: 10.1890/07-2092.1.
65. Burgin S, Wotherspoon D. The potential for golf courses to support restoration of biodiversity for BioBanking offsets. Urban Ecosystems. 2009;12(2):145-55. doi: 10.1007/s11252-008-0076-5.
66. Tanner RA, Gange AC. Effects of golf courses on local biodiversity. Landscape and Urban Planning. 2005;71(2):137-46. doi: https://doi.org/10.1016/j.landurbplan.2004.02.004.
67. Angiuli E, Trianni G. Urban Mapping in Landsat Images Based on Normalized Difference Spectral Vector. IEEE Geoscience and Remote Sensing Letters. 2014;11(3):661-5. doi: 10.1109/LGRS.2013.2274327.
68. Wu S-S, Qiu X, Usery EL, Wang L. Using Geometrical, Textural, and Contextual Information of Land Parcels for Classification of Detailed Urban Land Use. Annals of the Association of American Geographers. 2009;99(1):76-98. doi: 10.1080/00045600802459028.
69. Toole JL, Ulm M, González MC, Bauer D, editors. Inferring land use from mobile phone activity. Proceedings of the ACM SIGKDD international workshop on urban computing; 2012: ACM.

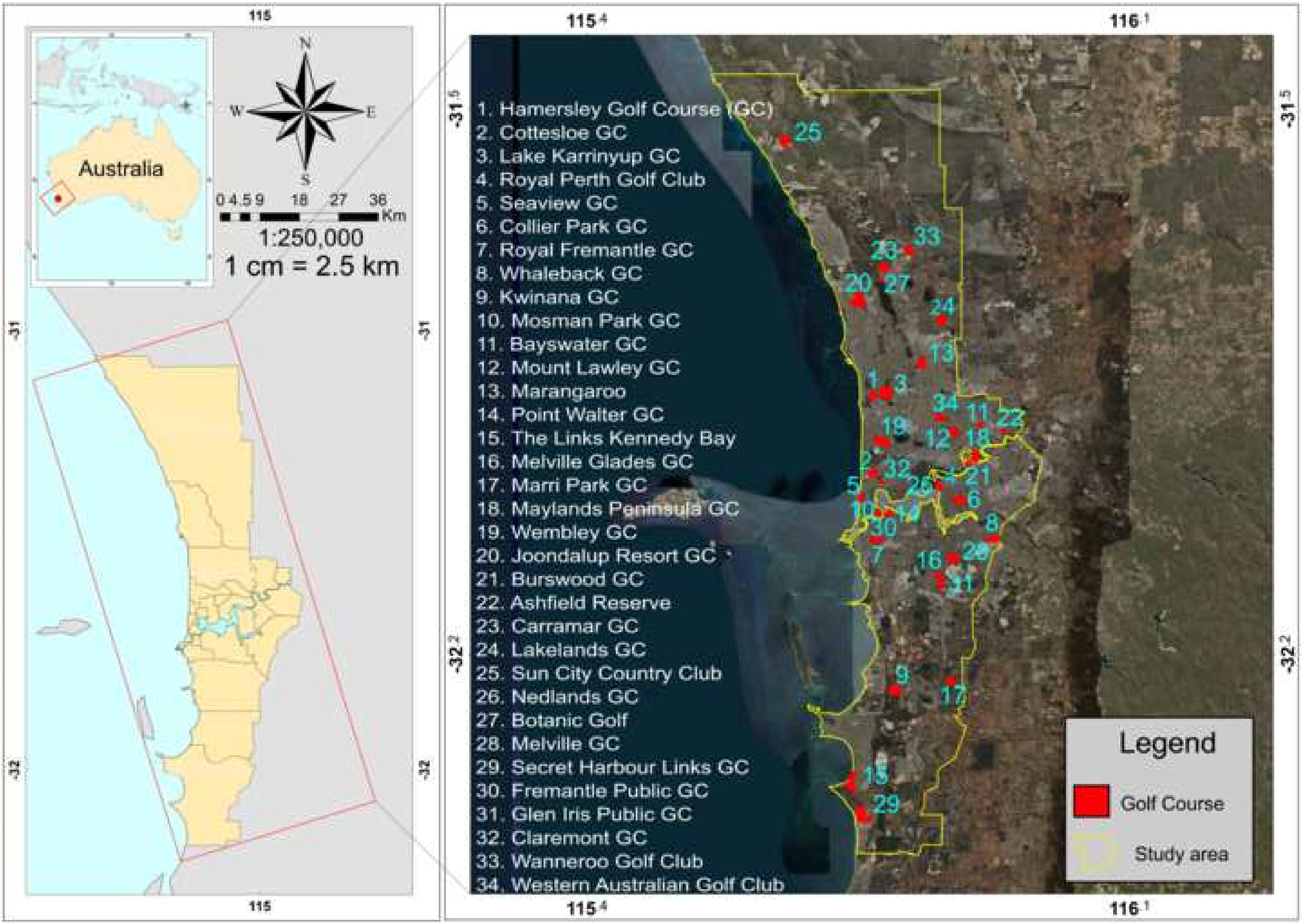
115
Australia
N
W
E
S
0 4.5 9 18 27 36 Km
1:250,000
1 cm = 2.5 km
-31
-32
115.4
116.1
-31.5
-32.2
1. Hamersley Golf Course (GC)
2. Cottesloe GC
3. Lake Karrinyup GC
4. Royal Perth Golf Club
5. Seaview GC
6. Collier Park GC
7. Royal Fremantle GC
8. Whaleback GC
9. Kwinana GC
10. Mosman Park GC
11. Bayswater GC
12. Mount Lawley GC
13. Marangaroo
14. Point Walter GC
15. The Links Kennedy Bay
16. Melville Glades GC
17. Marri Park GC
18. Maylands Peninsula GC
19. Wembley GC
20. Joondalup Resort GC
21. Burswood GC
22. Ashfield Reserve
23. Carramar GC
24. Lakelands GC
25. Sun City Country Club
26. Nedlands GC
27. Botanic Golf
28. Melville GC
29. Secret Harbour Links GC
30. Fremantle Public GC
31. Glen Iris Public GC
32. Claremont GC
33. Wanneroo Golf Club
34. Western Australian Golf Club
Legend
Golf Course
Study area

Figure 2 Click here to access/download;Figure;Fig.2.tiff

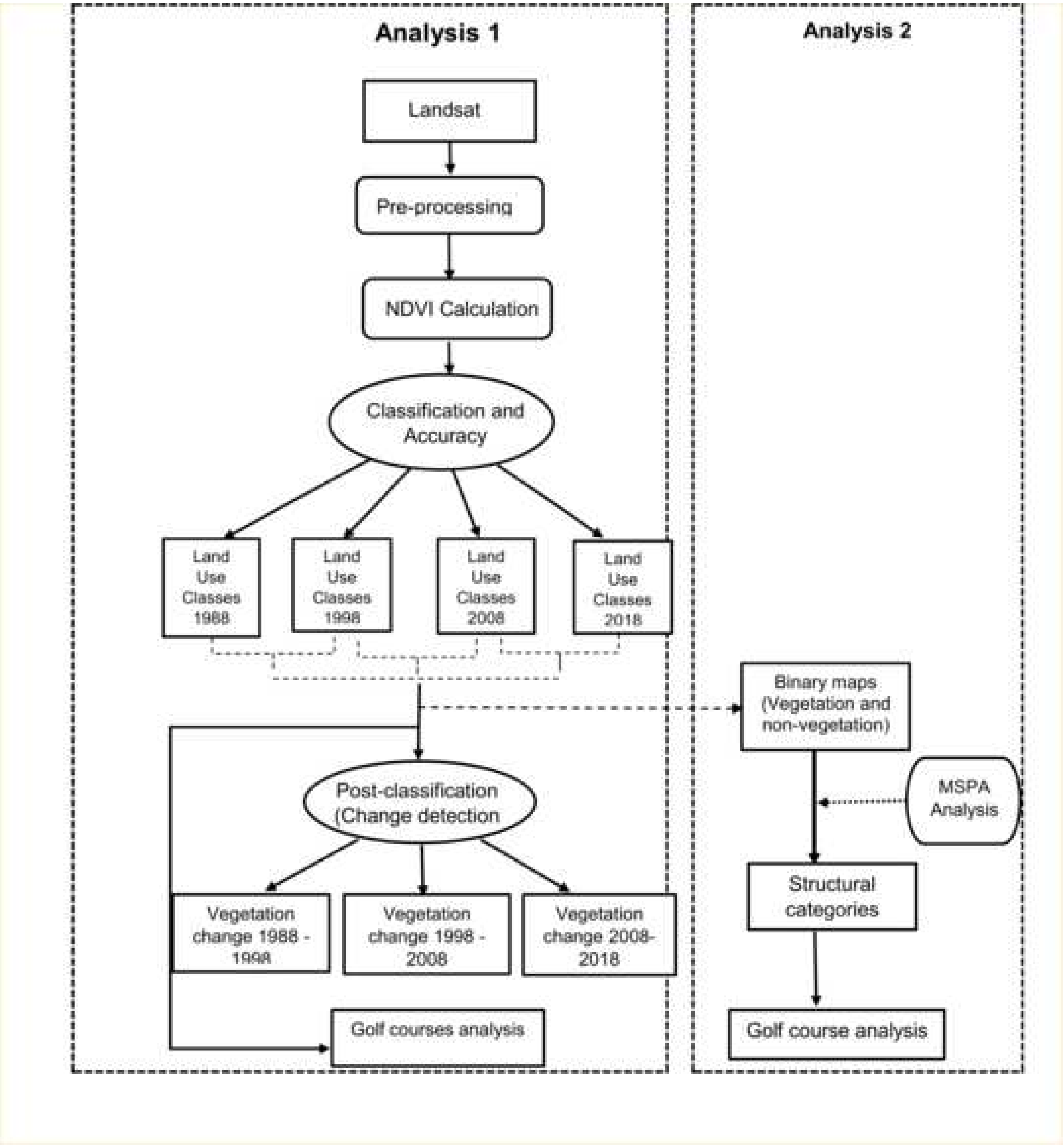

Figure 3

Click here to access/download;Figure;Fig.3.tiff

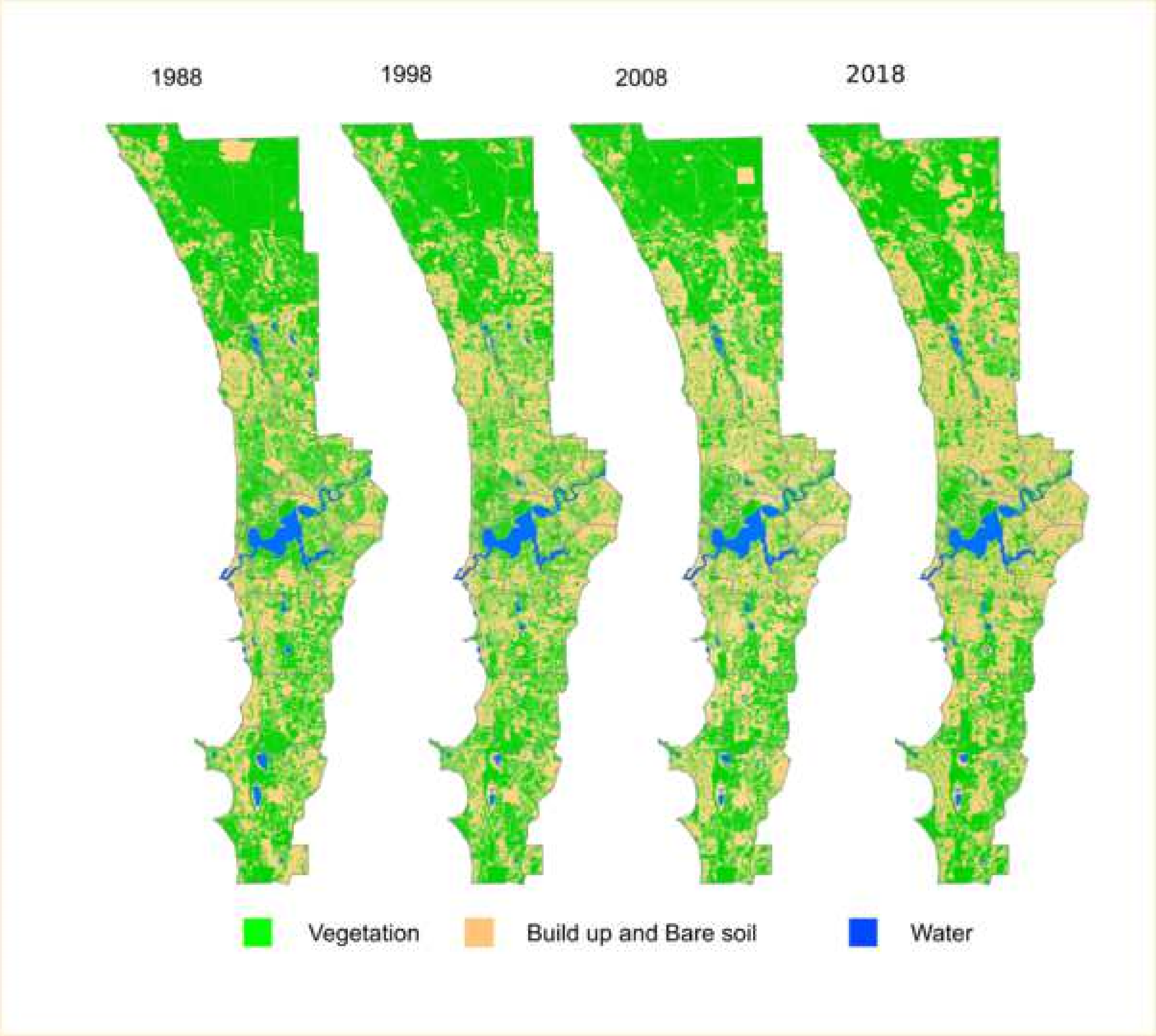

Figure 4

Click here to access/download;Figure;Fig.4.tiff

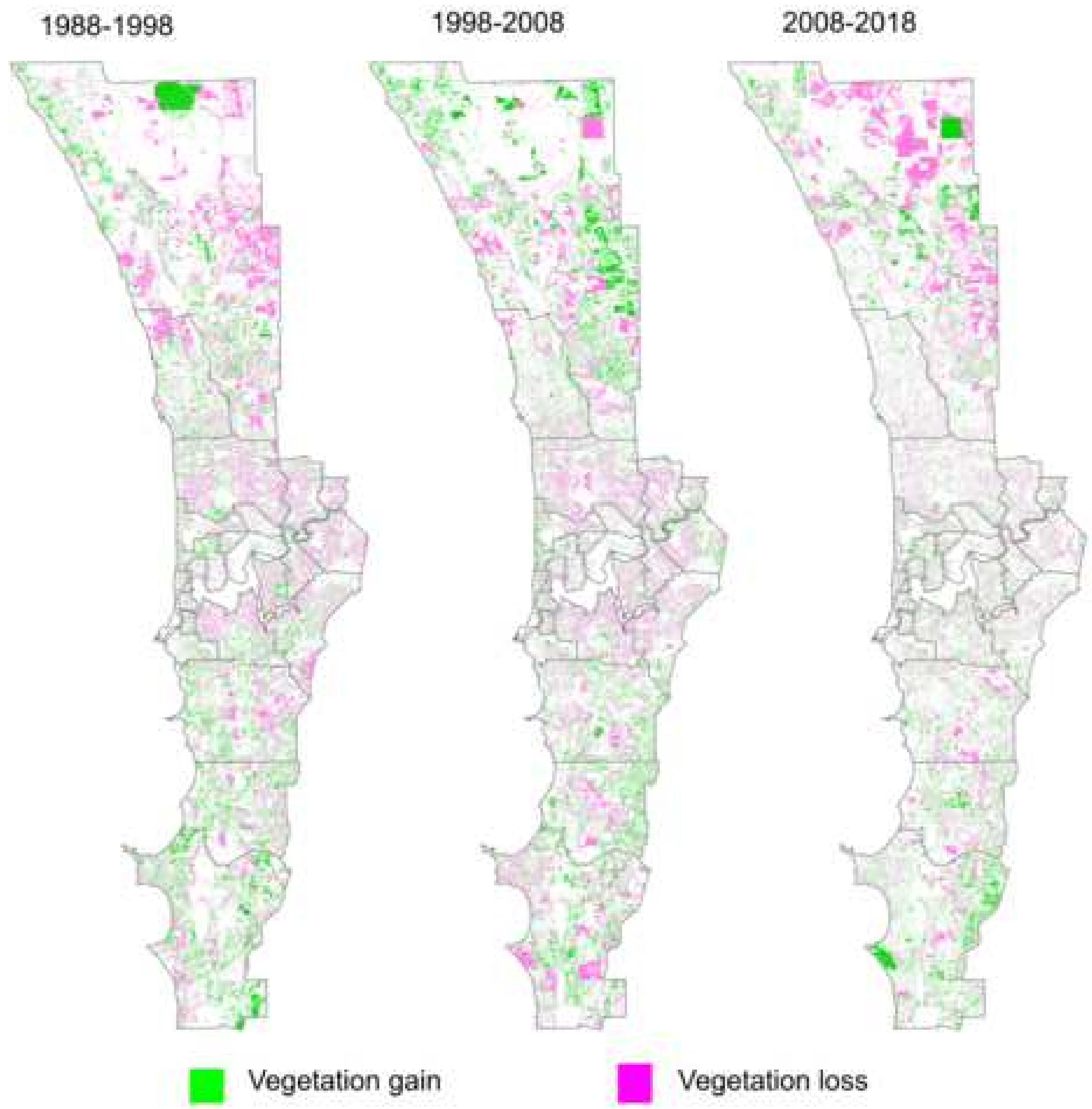

Figure 5

Click here to access/download;Figure;Fig.5.tiff

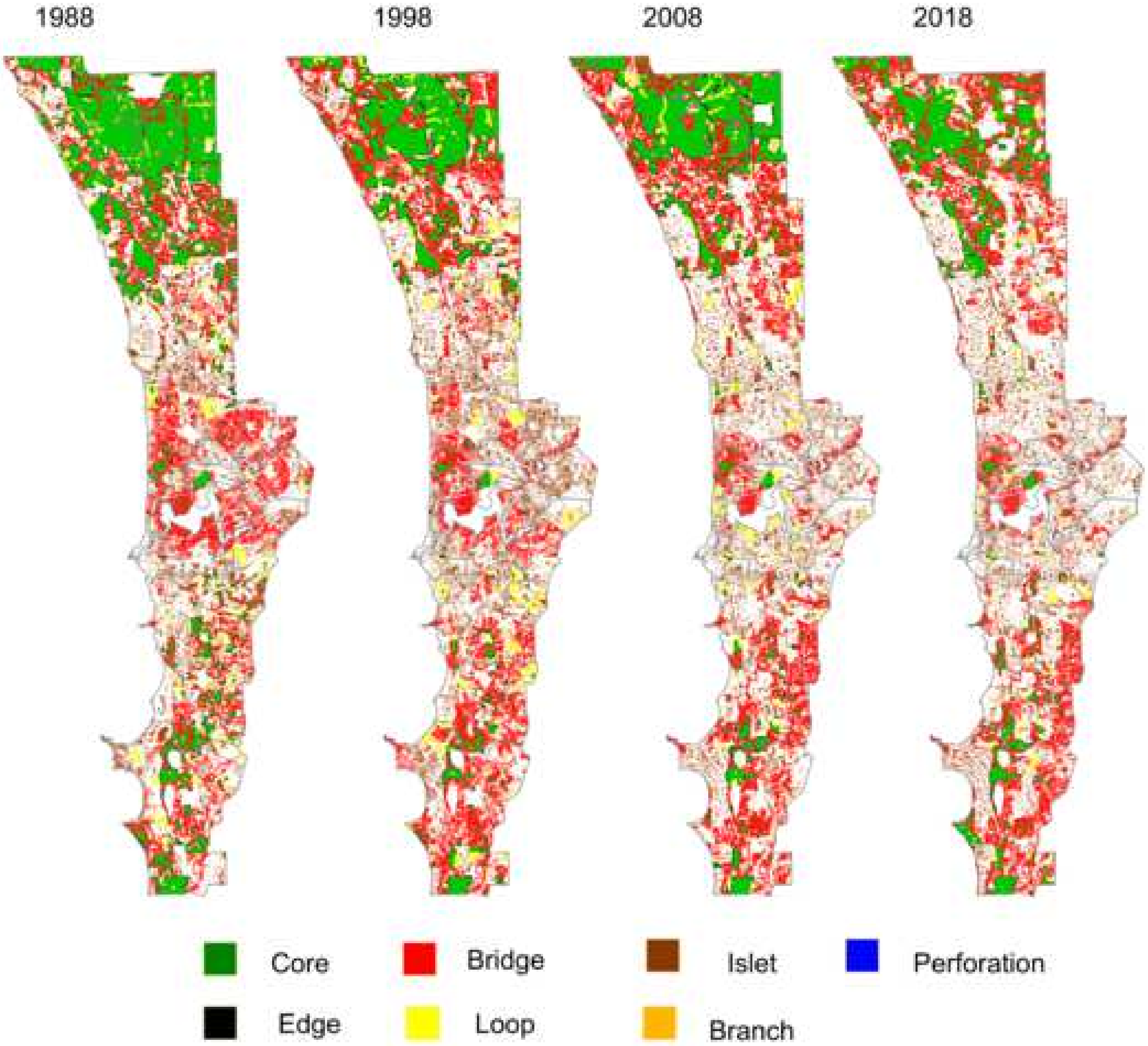

Supplement: Attachment — Submitted filename: PONE-D-19-19510_reviewer_Review_August2619.pdf [file pone.0228090.s001.pdf]
