# Supplementary material for: Vegetation trends associated with urban development: The role of golf courses
Source: PLoS One. 2020 Feb 5;15(2):e0228090. doi: 10.1371/journal.pone.0228090 (PMC7001943; doi:10.1371/journal.pone.0228090)
Supplement: Attachment — Submitted filename: PONE-D-19-19510_R1_reviewer_Reviewer.pdf [file pone.0228090.s004.pdf]

PLOS ONE

Vegetation trends associated with urban development: the role of golf courses

--Manuscript Draft--

**Manuscript Number:**	PONE-D-19-19510R1
**Article Type:**	Research Article
**Full Title:**	Vegetation trends associated with urban development: the role of golf courses
**Short Title:**	Urban development and the role of golf courses
**Corresponding Author:**	Thu T Nguyen<br>Vietnam National University of Forestry<br>Ha Noi, VIET NAM
**Keywords:**	Urban ecosystems, vegetation change, biodiversity connectivity, biodiversity hotpot, golf courses, remote sensing
**Abstract:**	Globally, cities are growing rapidly in size and density and this has caused profound impacts on urban forest ecosystems. Urbanization requiring deforestation reduces ecosystem services that benefit both city dwellers and biodiversity. Understanding spatial and temporal patterns of vegetation changes associated with urbanization is thus a vital component of future sustainable urban development. We used Landsat time series data for three decades from 1988 to 2018 to characterize changes in vegetation cover and habitat connectivity in the Perth Metropolitan Area, in a rapidly urbanising Australian biodiversity hotspot, as a case study to understand the impacts of urbanization on urban forests. Moreover, as golf courses are a major component in urban areas, we assessed the role of golf courses in maintaining vegetation cover and creating habitat connectivity. To do this we employed (1) land use classification with post-classification change detection, and (2) Morphological Spatial Pattern Analysis (MSPA). Over 17,000 ha of vegetation were cleared and the area of vegetation contributing to biodiversity connectivity was reduced significantly over the three decades. The spatial patterns of vegetation loss and gain were different in each of the three decades (1988-2018) reflecting the implementation of urban planning. Furthermore, MSPA analysis showed that the reduction in vegetation cover led to habitat fragmentation with a significant decrease in the core and bridge classes and an increase in isolated patches in the urban landscape. Golf courses played a useful role in maintaining vegetation cover and contributing to connectivity in a regional biodiversity hotspot. Our findings suggest that for future urban expansion, urban planning needs to more carefully consider the impacts of deforestation on connectivity in the landscape. Moreover, there is a need to take into consideration opportunities for off-reserve conservation in smaller habitat fragments such as in golf courses in sustainable urban management.
**Order of Authors:**	Thu Thi Nguyen
	Paul Barber
	Richard Harper
	Tran Vu Khanh Linh
	Bernard Dell
**Response to Reviewers:**	Reviewer 1: We have incorporated all of your comments into to our revision. They were very useful.<br>Reviewer 2: We have incorporated all of your comments into to our revision. Thank you for your helpful suggestions.<br>Reviewer 3: We have incorporated all of your comments into to our revision. Thank you for your help.<br>Reviewer 4: We have incorporated all of your comments into to our revision. They were very helpful.
**Additional Information:**	
**Question**	**Response**

Powered by Editorial Manager® and ProduXion Manager® from Aries Systems Corporation

**Financial Disclosure**<br><br>Enter a financial disclosure statement that describes the sources of funding for the work included in this submission. Review the submission guidelines for detailed requirements. View published research articles from *PLOS ONE* for specific examples.<br><br>This statement is required for submission and **will appear in the published article** if the submission is accepted. Please make sure it is accurate.<br><br>**Unfunded studies**<br>Enter: *The author(s) received no specific funding for this work.*<br><br>**Funded studies**<br>Enter a statement with the following details:<br>• Initials of the authors who received each award<br>• Grant numbers awarded to each author<br>• The full name of each funder<br>• URL of each funder website<br>• Did the sponsors or funders play any role in the study design, data collection and analysis, decision to publish, or preparation of the manuscript?<br>• **NO** - Include this sentence at the end of your statement: *The funders had no role in study design, data collection and analysis, decision to publish, or preparation of the manuscript.*<br>• **YES** - Specify the role(s) played.<br><br>* typeset	TTN received PhD scholarship co-funded by Vietnam and Murdoch University.
**Competing Interests**<br><br>Use the instructions below to enter a competing interest statement for this submission. On behalf of all authors, disclose any competing interests that could be perceived to bias this work—acknowledging all financial support and any other relevant financial or non-financial competing interests.<br><br>This statement **will appear in the published article** if the submission is accepted. Please make sure it is	A/Prof. Paul Barber is employed by the commercial entity ArborCarbon who provides in-kind<br>support only through the employment of the co-author A/Prof. Paul Barber and access to facilities,<br>equipment and software. This commercial affiliation does not alter our adherence to all PLOS ONE<br>policies in sharing data and materials. Prof. Dell, Prof. Harper and other authors have no<br>competing interests.

accurate. View published research articles from *PLOS ONE* for specific examples.<br><br>**NO authors have competing interests**<br><br>Enter: *The authors have declared that no competing interests exist.*<br><br>**Authors with competing interests**<br><br>Enter competing interest details beginning with this statement:<br><br>*I have read the journal's policy and the authors of this manuscript have the following competing interests: [insert competing interests here]*<br><br>* typeset	
**Ethics Statement**<br><br>Enter an ethics statement for this submission. This statement is required if the study involved:<br><br>• Human participants<br>• Human specimens or tissue<br>• Vertebrate animals or cephalopods<br>• Vertebrate embryos or tissues<br>• Field research<br><br>Write "N/A" if the submission does not require an ethics statement.<br><br>General guidance is provided below. Consult the submission guidelines for detailed instructions. **Make sure that all information entered here is included in the Methods section of the manuscript.**	No ethics approval required for the project.

**Format for specific study types**<br><br>**Human Subject Research (involving human participants and/or tissue)**<br>• Give the name of the institutional review board or ethics committee that approved the study<br>• Include the approval number and/or a statement indicating approval of this research<br>• Indicate the form of consent obtained (written/oral) or the reason that consent was not obtained (e.g. the data were analyzed anonymously)<br><br>**Animal Research (involving vertebrate animals, embryos or tissues)**<br>• Provide the name of the Institutional Animal Care and Use Committee (IACUC) or other relevant ethics board that reviewed the study protocol, and indicate whether they approved this research or granted a formal waiver of ethical approval<br>• Include an approval number if one was obtained<br>• If the study involved *non-human primates*, add *additional details* about animal welfare and steps taken to ameliorate suffering<br>• If anesthesia, euthanasia, or any kind of animal sacrifice is part of the study, include briefly which substances and/or methods were applied<br><br>**Field Research**<br><br>Include the following details if this study involves the collection of plant, animal, or other materials from a natural setting:<br>• Field permit number<br>• Name of the institution or relevant body that granted permission	
**Data Availability**<br><br>Authors are required to make all data underlying the findings described fully available, without restriction, and from the time of publication. PLOS allows rare exceptions to address legal and ethical concerns. See the PLOS Data Policy and FAQ for detailed information.	Yes - all data are fully available without restriction

A Data Availability Statement describing where the data can be found is required at submission. Your answers to this question constitute the Data Availability Statement and **will be published in the article**, if accepted.<br><br>**Important:** Stating 'data available on request from the author' is not sufficient. If your data are only available upon request, select 'No' for the first question and explain your exceptional situation in the text box.<br><br>Do the authors confirm that all data underlying the findings described in their manuscript are fully available without restriction?	
**Describe where the data may be found in full sentences. If you are copying our sample text, replace any instances of XXX with the appropriate details.**<br><br>• If the data are **held or will be held in a public repository**, include URLs, accession numbers or DOIs. If this information will only be available after acceptance, indicate this by ticking the box below. For example: *All XXX files are available from the XXX database (accession number(s) XXX, XXX.*).<br>• If the data are all contained **within the manuscript and/or Supporting Information files**, enter the following: *All relevant data are within the manuscript and its Supporting Information files.*<br>• If neither of these applies but you are able to provide **details of access elsewhere**, with or without limitations, please do so. For example:<br><br>*Data cannot be shared publicly because of [XXX]. Data are available from the XXX Institutional Data Access / Ethics Committee (contact via XXX) for researchers who meet the criteria for access to confidential data.*<br><br>*The data underlying the results presented in the study are available from (include the name of the third party*	All relevant data are within the manuscript

and contact information or URL).<br>• This text is appropriate if the data are owned by a third party and authors do not have permission to share the data.<br><br>* typeset	
Additional data availability information:	

Manuscript Click here to access/download;Manuscript;Manuscript.docx

Vegetation trends associated with urban development: the role of golf courses

Thu Thi Nguyen[1,3*], Paul Barber[2,3], Richard Harper[3], Tran Vu Khanh Linh[4], Bernard Dell[3]

[1] Vietnam National University of Forestry (VNUF), Xuan Mai, Chuong My, Ha Noi, Vietnam,
[2] ArborCarbon Pty Ltd, 1 City Farm Place, East Perth, WA 6004, Australia
[3] Agricultural and Forestry Sciences, Murdoch University, Murdoch, WA 6150, Australia
[4] Faculty of Forestry, Nong Lam University - Ho Chi Minh City, Thu Duc District, Ho Chi Minh City, Vietnam

*Corresponding author

Emails: Thu.Nguyen@murdoch.edu.au; thu.nguyen.2k14@gmail.com

Abstract

Globally, cities are growing rapidly in size and density and this has caused profound impacts on urban forest ecosystems. Urbanization requiring deforestation reduces ecosystem services that benefit both city dwellers and biodiversity. Understanding spatial and temporal patterns of vegetation changes associated with urbanization is thus a vital component of future sustainable urban development. We used Landsat time series data for three decades from 1988 to 2018 to characterize changes in vegetation cover and habitat connectivity in the Perth Metropolitan Area, in a rapidly urbanising Australian biodiversity hotspot, as a case study to understand the impacts of urbanization on urban forests. Moreover, as golf courses are a major component in urban areas, we assessed the role of golf courses in maintaining vegetation cover and creating habitat connectivity. To do this we employed (1) land use classification with post-classification change detection, and (2) Morphological Spatial Pattern Analysis (MSPA). Over 17,000 ha of vegetation were cleared and the area of vegetation contributing to biodiversity connectivity was reduced significantly over the three decades. The spatial patterns of vegetation loss and gain were different in each of the three decades (1988-2018) reflecting the implementation of urban planning. Furthermore, MSPA analysis showed that the reduction in vegetation cover led to habitat fragmentation with a significant decrease in the core and bridge classes and an increase in isolated patches in the urban landscape. Golf courses played a useful role in maintaining vegetation cover and contributing to connectivity in a regional biodiversity hotspot. Our findings suggest that for future urban expansion, urban planning needs to more carefully consider the impacts of

deforestation on connectivity in the landscape. Moreover, there is a need to take into consideration opportunities for off-reserve conservation in smaller habitat fragments such as in golf courses in sustainable urban management.

1 Introduction

Globally, cities have grown rapidly in number and size over recent decades [1, 2]. This trend is predicted to continue as urban areas are expected to absorb most global population growth [3]. While the process of urbanization presents key implications for changes in physical landscapes and demographic characteristics, it can cause profound impacts on environmental components, especially on urban forest ecosystems [4, 5].

Vegetation in urban landscapes is critically important because provides goods and services, and full ecosystem functions that benefit city dwellers and the environment. On the one hand, a remarkable range of human well-being benefits are derived from urban green spaces including mitigating the urban heat island (UHI) effect which is a threat to human health [6, 7]; reducing stress [8], improving healing times [11], increasing self-esteem and empowerment [12], and improving cognitive ability [13]. On the other hand, urban green spaces provide various ecosystem services such as strengthening resistance to some kinds of natural disasters for example, floods [14], promoting biological processes such as pollination [15], and reducing surface erosion from stormwater runoff [16]. The amount of vegetation in cities strongly influences biodiversity, especially where vegetation is set aside during the process of urbanization [17]. However, if species dispersal and exchange among these patches is insufficient to allow gene flow and diversity, loss of regional biodiversity is inevitable [18]. Therefore, urban development that requires deforestation, with habitat loss and fragmentation is a threat to biodiversity [17, 19].

Urban conservation strategies must therefore consider not only the size and quality of habitat reserves, but the connectivity in the intervening urban vegetation matrix [19]. While the need to protect large habitat reserves is obvious, opportunities for off-reserve conservation of smaller habitat fragments should not be overlooked [20]. Understanding spatial and temporal patterns of vegetation change

associated with urbanization, as well as opportunities for the preservation of green spaces outside natural reserves, is vital for future sustainable urban development especially in areas of global ecological importance.

As the number of golf courses is rapidly increasing in many urban areas worldwide [21], there have been many environmental arguments about this green space category in urban landscapes. Golf courses are sometime considered to be major polluters of the environment through pesticide and fertilizer use [22]. In fact, golf courses have been established for recreational purposes, which are a mix of bushland, fairways and infrastructure. Though they are not fully ecologically functional as with a natural parks, previous studies investigated the condition of vegetation inside the golf courses and indicated that the bushland in non-playing areas of golf courses are significantly important to biodiversity conservation and the provision of ecosystem services in cities [24], such as providing refuge habitats for urban-avoiding wildlife [23, 25, 26]. Therefore, potentially, together with the natural reserves, golf courses can play some roles as off-reserve sites for purposeful biodiversity conservation in urban landscapes. Nevertheless, little research has been undertaken to comprehensively assess the role of golf courses in maintaining vegetation patches as interconnected nodes in urban landscapes during a long period of urban development where deforestation was significant.

Deforestation for urban expansion can occur gradually over multiple years. Satellite-based remote sensing holds certain advantages in the characterization of these changes in urban landscapes because of the large spatial coverage, high time resolution, and wide availability of data [27]. For example, medium resolution Landsat images allow mapping of large urban areas [28]. Many methods have been used to detect, monitor and quantify vegetation changes, but differences in vegetation index and land use classification are the most widely used methods for vegetation change over a long period of time [28].

A great number of vegetation indices have been proposed, ranging from very simple to very complex band combinations [29]. The most widely-used vegetation index is the Normalized Difference Vegetation Index (NDVI), which is the most efficient and simple metric to identify vegetated areas and their condition [30]. This separates green vegetation from other surfaces based on the ability of

the differential absorption of red by chlorophyll and reflection of NIR by green vegetation.

chlorophyll to absorb red light for photosynthesis and reflect the near-infrared (NIR) wavelengths [31]. Furthermore, information on vegetation cover dynamics can be combined with Morphological Spatial Pattern Analysis (MSPA) to describe the spatial configuration of the ecosystem at the pixel level, making it possible to detect temporal changes in the structural connectivity of habitats in urban settings [32].

Therefore, to enhance the understanding of vegetation dynamics associated with urbanization and the role of golf courses in maintaining urban forests, we used Landsat imagery to map vegetation cover and to assess its spatial and temporal distribution over three decades from 1988 to 2018. Maps of vegetation cover were used for MSPA analysis to detect changes in habitat connectivity. We chose the Perth Metropolitan area for the study as it lies within a rapidly urbanizing biodiversity hotspot in Australia. The three primary research objectives were to: (1) determine the spatial and temporal patterns of deforestation in urbanization; (2) evaluate the spatial and temporal patterns of green landscape connectivity; and (3) assess the role of golf courses in preserving green spaces and biodiversity in an urban landscape. This analysis will provide a useful perspective on the land-use pressure facing vegetation remnants in this region which is recognised as one of 35 international biodiversity hotspots with over 1500 plant species with a high degree of endemism [33], and provide a framework for planning urban expansions both in this region and globally.

2 Methods

2.1 Study area

The study area belongs to the Perth Metropolitan region covering four sub-regions (North West, Middle Central, Inner Central and South West). Perth has a Mediterranean-type temperate climate with a hot and dry summer, and a cold and rainy season occurring between May and October [34]. Under future climate-change scenarios, this area is projected to experience a lower annual rainfall [35].

Perth belongs to the Australia's southwest corner, which is recognized as a global biodiversity "hotspot" with outstanding natural environments. Our study area occurs on the Swan Coastal Plain which is part of the southwest of Australia which in turn has the highest concentration of rare and endangered species on the entire continent (at least 1,500 endemic species). More than 6,000 species of native plants and

100 native mammals, birds, frogs and reptiles occur in this region, making it a biodiversity "hotspot" [33, 36].

Perth has retained a significant area of natural vegetation which has conservation significance thanks to the introduction of legislation and policies aimed primarily at protecting biophysical environmental values. This includes the Western Australian *Environmental Protection Act* 1986 [37]; the Federal Government's *Environment Protection and Biodiversity Conservation Act* 1999 [38]; and more recently, Bush Forever [39, 40], a policy which took a whole-of-government approach to identify and protect biologically significant bushland and wetlands within the Perth metropolitan area. However, Perth has experienced extensive urban development since the 1980s [42]. The expansive growth of the Perth metropolitan footprint has contributed to the loss of biodiversity, together with the ecosystem services provided by natural areas. Together with urbanization, the golf industry has expanded contributing to a growing proportion of Perth's urban green space with 34 golf courses in the study area (Figure 1).

Excellent additions

Figure 1. Location of the study area

(The Landsat 8 on 30 September 2018 from USGS EROS (Earth Resources Observatory and Science (EROS) Center) in the public domain: http://eros.usgs.gov)

2.2 *Approaches*

We conducted two types of analyses in this study: (1) assessment of vegetation cover change in golf courses since 1988 relative to surrounding areas to provide a broad overall context of the urban vegetation changes that have taken place throughout the region and the role of golf courses; and (2) assessment of MSPA. In these analyses, we used four data sets (1988, 1998, 2008, 2018) covering thirty years of urban development. The steps taken in this study are summarized in a flow chart (Figure 2).

2.2.1 Landsat data and pre-processing

Three Landsat 5 Thematic Mapper images (WRS path 113 row 82) were acquired from 1988 to 2008 and one Landsat OLI 8 (WRS path 113 row 82) was acquired in 2018 at four time steps, including year 1988 (11th December), year 1998 (7th December), year 2008 (18th December), year 2018 (14th December). The Landsat imagery were obtained from the US Geological Survey (USGS) of the Earth

Resources Observation and Science Center (EROS). Image dates were selected acquired during December (summer, dry season) to reduce the seasonal difference effects. All images selected are cloud-free scenes or little cloud cover (0.05% and 0.4%) scenes with the whole study area is cloud-free; Therefore, is no requirement for removing cloud. Georeferencing was performed at the USGS prior to downloading the data (L1T level of systematic geometric accuracy) and no further refinement was undertaken. Atmospheric and topographic corrections were performed on the Landsat data sets. The atmospheric correction was carried out to adjust the multitemporal dataset to a common radiometric scale [43].

The first process of atmospheric correction was conversion of the digital number (DN) remote sensing data values to at-sensor radiance based on the image header file. After that we employed the image-based models - dark object subtraction (DOS) to correct atmospheric scattering scene-by-scene. This method is a widely used and effective method in atmospheric correction [44-50]. Topographic correction was conducted to remove topographic effects. We used a sun-canopy-sensor (SCS) correction based on the 30 m digital elevation model (DEM) because topographic shading is not only due to slope but also to shadowing of one tree crown over another and this is one of the most widely and effective used methods of topographic correction [51].

Figure 2. Flow chart summarizing the major steps taken during the investigation.

2.2.2 Classification

In a preliminary step, we used a decor relation stretch to enhance the image for more effective visualization. Prior to image classification, NDVI images were generated. A classification technique was then applied to the NDVI images of 1988, 1998, 2008 and 2018 using Arc-GIS 10.3 software. NDVI images were obtained by calculating the ratio between the red (R) and near infrared (NIR) values of the satellite image using Equation 1:

$$\mathrm{NDVI} = \frac{(\mathrm{NIR-R})}{(\mathrm{NIR+R})} \qquad (1)$$

In Landsat 4-7, NDVI = (Band 4 – Band 3) / (Band 4 + Band 3).

In Landsat 8, NDVI = (Band 5 – Band 4) / (Band 5 + Band 4).

Landsat TM data from different dates were independently classified based on the NDVI values. Water bodies have negative NDVI values, whereas, bare soil and built-up areas have an NDVI value of around zero. Chlorophyll in green vegetation, on the other hand, has consumed RED to drive photosynthesis thereby providing moderate and high NDVI values close to +1 [52]. Based on this understanding, the four NDVI images were classified into three classes (Vegetation, Built up + bare soil, Water bodies) using the NDVI threshold ranges technique in Arc-GIS 10.3 software.

The classification based on NDVI threshold was evaluated using accuracy assessment. An error matrix compared information from a classified image or land cover map to known reference (truth) sites for a number of sample points assessed in 2018. We obtained photographs of representative land use categories with GPS locations to assist in our image interpretations. Also, we used Google Earth images, true and false colour combination images and knowledge-based information including expert knowledge, land use maps and reports. For historical images, Google Earth was used to substitute the traditional reference data collection on each of the sites [53]. Based on data of accuracy assessment, we reclassified the preliminary land use classification maps to improve the accuracy of classification.

2.2.3 Vegetation change detection

In order to detect the vegetation cover change, we created binary maps of vegetation and non-vegetation from the classified maps in the previous analysis, one for each adjacent pair of time steps, which depict where degradation occurred within a decade of urbanization. This post-classification analysis uses two images from different dates and classifies them independently. We then calculated changes in vegetation cover type using Equation 2:

$$\text{Change area} = D_2 - D_1 \qquad (2)$$

where D1 and D2 are the area of the target vegetation cover at the beginning and the end of the study period, respectively. This analysis allows the calculation of vegetation loss and gain in each period.

2.2.4 Golf courses Analysis 1

We compared the vegetation cover, and the change (vegetation loss and gain) taking place within all golf courses in the study area, and in their surrounding regions. After creating the GIS boundaries of the golf courses, we extracted the vegetation cover and the vegetation change within these boundaries at four time steps in 1988, 1998, 2008 and 2018 to compare vegetation dynamics within the golf courses and the whole study area over time.

2.2.5 Morphological Spatial Pattern Analysis (MSPA) for the structural connectivity of habitats

MSPA was employed to describe the structural connectivity of habitats in Perth for four time steps in 1988, 1998, 2008 and 2018. This method describes the spatial and temporal configuration of the ecosystem at the pixel level [32], which was based on the concept of "habitat availability" and "graphic theory" [54, 55] in which the landscape is considered as a collection of nodes, and links with a node is a place where connectivity exists and will depend on the width of itself. The output of the MSPA analysis includes the seven structural categories into which habitats are divided, including core, edge, perforation, bridge, loop, branch and islet [56] and [57] and is summarized in Table 1.

Table 1. Definition of Morphological Spatial Pattern Analysis (MSPA) classes

Class	Description	Ecological Meaning
Core	A collection of foreground pixels which are interconnected and greater than the user-specified edge width from background	Large-scale natural patches with high connectivity
Edge	Transition pixels between the foreground and background that form the outer edge	The transition zone between vegetation and non-vegetation areas.
Perforation	The transition pixels between foreground and background inside core areas that form the inner edge	Unnatural patch inside the core area.
Bridge	A set of linear foreground pixels between two cores that connect two or more core areas.	The striped ecological land that connects two cores, which is equivalent to the connecting corridor of the green space network.
Loop	Linearly oriented foreground pixels extended from core that connects core area to itself.	Connecting corridor inside a large natural patch.
Branch	Linearly oriented foreground pixels extended from core that do not connect to any other core area	Striped ecological land with low connectivity.
Islet	A collection of foreground pixels which is smaller than the core zone and do not connect to any other foreground cells.	Small natural patches that are isolated and do not connect to each other.

In order to undertake the MSPA analysis, we defined the input data (foreground class). For this study, we used the classified maps for 1988, 1998, 2008, 2018 in Analysis 1 to create the binary maps which contained vegetation and non-vegetation classes. Hence, the high and full covered vegetation pixels were defined as the foreground pixels (green landscape) in the MSPA approach. The results of MSPA

analysis for the four time steps allowed us to assess the changes in habitat connectivity over 30 years of urbanization.

2.2.6 Golf course analysis 2

To assess the role of golf courses in maintaining biodiversity connectivity over 30 years, we compared the habitat connectivity within all golf courses in the study area and in their surrounding green spaces. Using the GIS boundaries of the golf courses, we extracted the habitat connectivity within the golf course boundaries for four time steps (1988, 1998, 2008 and 2018).

3 Results

3.1 Land use classification in Perth

Data sets representing four time periods (1988, 1998, 2008 and 2018) are shown in Figure 3 and Table 3, which provide an overview of the land cover changes (vegetation, built up and bare land, water bodies) over recent decades. From the 1988 and 1998 data sets, it is evident that over half of the region was vegetated. However, the urban footprint of built up and bare land area had increased 10% from 1998 to 2018. As a consequence, there was a significant decrease in vegetation cover, which comprised 56% of the land surface in 1988 and declined by 10.1% over the next 30 years (Table 2).

Table 2. Land cover classification within the Perth Metropolitan Region.

		Land cover category					
		Whole area			Golf course		
		Vegetation	Built up and bare soil	Water bodies	Vegetation	Built up and bare soil	Water bodies
1988	Area (ha)	98,446	74,376	2,667	929	210	0.1
	Proportion (%)	56.1	42.4	1.5	81.5	18.5	0.0
1998	Area (ha)	91,754	81,464	2,271	1,042	97	0.7
	Proportion (%)	52.3	46.4	1.3	91.4	8.5	0.1
2008	Area (ha)	88,341	84,971	2,176	1,084	55	0.2
	Proportion (%)	50.3	48.4	1.2	95.1	4.9	0.0
2018	Area (ha)	80,755	92,243	2,491	1,093	46	0.7
	Proportion (%)	46.0	52.6	1.4	95.9	4	0.1

Figure 3. Map of land cover classification for Perth in four time steps between 1988 and 2018

The analysis had the overall accuracy (OA) of classification from 87% and the kappa coefficient from 91% for the three classes (Table 3).

Table 3. Accuracy assessment of land cover maps generated

Year 1988

LULC Class	Vegetation	Build Up and Bare Soil	Water Bodies	Total
Vegetation	14	1	0	15
Build Up and Bare Soil	1	12	2	15
Water Bodies	0	0	15	15
Total	15	13	17	45
		Overal Accuracy	87%	
		Overal Kappa	0.91	

Year 1998

LULC Class	Vegetation	Build Up and Bare Soil	Water Bodies	Total
Vegetation	15	0	0	15
Build Up and Bare Soil	0	13	2	15
Water Bodies	1	0	14	15
Total	16	13	16	45
		Overal Accuracy	90%	
		Overal Kappa	0.93	

Year 2008

LULC Class	Vegetation	Build Up and Bare Soil	Water Bodies	Total
Vegetation	14	1	0	15
Build Up and Bare Soil	1	14	0	15
Water Bodies	0	0	15	15
Total	15	15	15	45
		Overal Accuracy	93%	
		Overal Kappa	0.96	

Year 2018

LULC Class	Vegetation	Build Up and Bare Soil	Water Bodies	Total
Vegetation	15	0	0	15
Build Up and Bare Soil	0	13	2	15
Water Bodies	1	1	13	15
Total	16	14	15	45
		Overal Accuracy	87%	
		Overal Kappa	0.91	

I highly suggest the tables be restructured to show change in each class for all years on the same tables/Columns. This will make is easier to read and understand.

Using the GIS layer of land use categories, we also extracted the data for three classes within the golf courses in comparison with the whole area (Table 2). Our analysis shows that while there was a significant decrease in vegetation cover throughout the region, the total area of golf courses remained unchanged at around 1,093 ha over the last 30 years.

Figure 4. Land cover classification showing a detailed view of the Collier Park Golf Course for a) Landsat data; b) NDVI values; c) Land cover classification

3.2 Spatial patterns of vegetation change

To characterize spatial patterns of vegetation dynamics, we detected the vegetation loss and gain for each of the three decades (Figure 5). In the period 1988 to 1998, deforestation occurred intensively in the central and north regions. From 1998 to 2008, vegetation loss expanded to the north and south of the city. However, in the last decade from 2008 to 2018, vegetation loss accelerated in the distal regions with urbanization. However, there was also some vegetation gain over the three decades (Figure 5, Table 4) predominantly in the northern part of the city.

Figure 5. Change in vegetation cover over the period 1988 to 2018 for the Perth region

Table 4. Vegetation loss and gain (ha) between 1988 and 2018.

		Period		
		1988-1998	1998-2008	2008-2018
Whole Area	Vegetation loss	22,231	19,250	20,178
	Vegetation gain	15,538	15,838	12,591
	Net loss	6,692	3,412	7,587
Golf courses	Vegetation loss	68	27	40
	Vegetation gain	219	92	43
	Net gain	152	64	4

Calculation of changes within the golf courses and in the whole area (Table 4) showed that the major urban area of Perth experienced a net loss in vegetation cover. Though vegetation compensation occurred together with deforestation over 30 years of urbanization, the vegetation loss was always much larger than vegetation gain with the largest net vegetation loss occurring in the last decade. However, the golf courses showed a different trend where the net gain of vegetation cover happened over three decades and the largest gain was between 1988 and 1998.

3.3 Analysis of connectivity components of green space networks

Results of the MSPA analysis indicate that the reduction in vegetation cover over the last thirty years has led to a decline in connectivity (Table 5) . Among the two MSPA classes that are important for connectivity (core and bridge), the total area of core class decreased by about 10% over three decades while the bridge class was maintained at around 37,000 ha, but the proportion of this class per total vegetation cover area (VCA) increased due to the reduction of vegetation cover over time (Table 5).This analysis also shows the fluctuation in the areas of the rest of the MSPA classes including islets, loops, edges, perforations, and branches which do not contribute to connectivity in the landscape. The proportion of these classes increased through time from 24% in 1988 to 30% in 2008 and 2018.

Figure 6 shows that the core area was distributed mostly in the northern part of the city and their areas decreased significantly in later years. In 1988, the bridge class covered a large area of the city's central region but this decreased over time. In recent years, most of the vegetation cover in the central region of the city belongs to the islet, loop, edge, perforation and branch classes, illustrating that isolation became more serious in the central region of the city over the three decades.

Figure 6. Results of the Morphological Spatial Pattern Analysis (MSPA) for the Perth region from 1988 to 2018

The vegetation cover within golf courses also contributes to connectivity. The proportion of core area within golf courses fluctuated between 12% and 27%. Moreover, the largest proportion of vegetation in golf courses was classified as bridge but it experienced a downward trend from 52% to 41% in three decades. Of the remaining classes which do not contribute to connectivity, the edge and loop classes accounted for a higher proportion with each of them contributing 7% to 16% of total vegetation cover.

Table 5. Results of Morphological Spatial Pattern Analysis (MSPA) analysis of connectivity of Perth's vegetation from 1988 to 2018.

Landscape type	Year	Perth Metropolitan Region		Within golf courses	
		Area (ha)	Proportion of total VCA (%)	Area (ha)	Proportion of total VCA (%)
Core	1988	32,012	32.6	325	23.2
	1998	20,607	22.5	171	12.7
	2008	22,251	25.3	230	16.7
	2018	18,101	22.5	383	27.3
Bridge	1988	37,127	37.9	738	52.6
	1998	39,412	43.0	814	60.2
	2008	37,902	43.1	732	53.1
	2018	37,503	46.7	588	42.0
Islet	1988	10,396	10.6	31	2.2
	1998	16,436	17.9	78	5.8
	2008	12,922	14.7	15	1.1
	2018	12,349	15.4	37	2.7
Perforation	1988	1,099	1.1	3	0.2
	1998	486	0.5	-	-
	2008	834	1.0	-	-
	2018	406	0.5	6	0.4
Edge	1988	7,430	7.6	162	11.5
	1998	4,814	5.3	95	7.0
	2008	4,986	5.7	147	10.7
	2018	5,218	6.5	230	16.4
Loop	1988	5,670	5.8	112	8.0
	1998	6,295	6.9	146	10.8
	2008	5,607	6.4	209	15.2
	2018	3,669	4.6	129	9.2
Branch	1988	4,361	4.5	32	2.3
	1998	3,619	4.0	47	3.5
	2008	3,491	4.0	43	3.2
	2018	3,117	3.9	29	2.1

4 Discussion

We used Landsat imagery to characterize the patterns of vegetation change, habitat connectivity and the role of golf courses in maintaining green spaces and connectivity in an urban landscape. We found that deforestation led to a reduction in habitat connectivity in the Perth Metropolitan Region. However, golf courses can play an important role in maintaining vegetation and supporting biodiversity connectivity in urban landscapes. Previous studies have documented the increase in the urban footprint of Perth using multi-temporal urban expansion statistics derived from Satellite imagery [58]; However, their work did not address the issues of vegetation dynamics, nor the biodiversity and structure of the vegetation and habitat connectivity in the Perth Metropolitan Region. Therefore, our study addresses this gap.

4.1 Deforestation and urbanization

The vegetation dynamics in the major urban areas of Perth reflect the pattern of urbanization over time. The reduction in green space found in this study can be explained as a close relation to the process of development in this city. Over the last three decades, urban development in Perth has taken place at an unsustainable rate [42]. In the early 1990s to 2006, Perth's population grew by around 1.8%, but the figure has nearly doubled since then [59]. Also, this study indicated that, from 1988 to 2018, Perth's urban footprint increased from 74,376 to 92,243 ha (Table 2) and is consistent with previous research in urban growth in this region [58]. In the last 20 years, on average, 740 ha/yr of urban and urban deferred zoned land was consumed by subdivision, and 830 ha/yr was consumed by construction in the Perth metropolitan area and nearby Peel region [60]. In addition to this expansion, the city has become denser with the construction of new residential dwellings in urbanised areas. Although vegetation gain occurred in some places as a result of natural increase in canopy cover as urban vegetation grows through the conservation efforts, vegetation offset from development projects (e.g. mining), the plantation programs of the government taking place in bare soils in some suburbs, and efforts to increase green spaces from private land owners, the vegetation loss associated with urbanization has been more significant.

The difference in spatial patterns of vegetation loss are also related to the urban plans of this city. Our results show that between 1988 and 1998, there was significant vegetation loss in the central region of the city which can be linked to The Corridor Plan [61]. Historically, Perth's development pattern from

1970s to 1990s was based on linear corridors stretching out from the city's core, with large non-urban wedges between each of these corridors [61]. However, from 1998 to 2008 and from 2008 to 2018, deforestation was more significant in the outer subregions north-west and south-west of the city due to the adoption of Metroplan [62]. Perth recently has been divided into subregional areas, rather than corridors for planning purposes. The two coastal subregions (the North-West and South-West) included in our study have consistently achieved higher rates of population growth under Metroplan [63].

Originally the region was covered by woodlands dominated by eucalypts and banksias and coastal heath interspersed with chains of wetlands. Perth is home to a rich biodiversity, with more than 1,700 species of flowering plants and iconic species of threatened fauna in the region [64]. Therefore, such deforestation for urbanization has resulted in the devastating loss of significant natural habitats in this biodiversity hotspot city, leading to the designation as an endangered ecological community by the Australian Government [41].

4.2 Connectivity of green space networks in urban landscapes

The MSPA analysis indicates that the loss of vegetation as a consequence of urban expansion has led to a marked reduction in connectivity of green space networks in the Perth urban landscape. As only cores (the stepping stones between forest habitat patches) and bridges (the structural corridors to link core areas) can contribute to the connectivity between the habitat areas in the landscape [65], the reduction of these areas in Perth associated with urbanization throughout the time indicates the high impact of urban development on habitat connectivity. This analysis also shows the increase in proportion of islets, which are totally isolated patches, and other classes (perforations, loops, branches, edges) that cannot reach a new core habitat area for originating the potential movement [65]. Clearly, expansion of Perth city has fragmented the remaining blocks of natural habitat and increased isolation of natural habitats. This may reduce population and gene flow among patches and may disrupt the connection between subpopulations and a large regional population [66] and thus threaten the long-term viability of relict populations.

Fragmentation was obvious in the central region of the city and in recent decades it has become more serious in the outer parts of the city. This is critical as Perth is within a globally recognised biodiversity

hotspot, which is home to rich biodiversity found nowhere else in the world. The connectivity in the major urban area of Perth is not only critical for the linkage of habitats within the Swan Coastal Plain but also for the connection of these coastal habitats to a large regional biosphere in south-western Australia.

The results also illustrated that a 'Core' area and a network of 'Bridge' types exist in the central and outer subregions of Perth. This is the consequence of early conservation efforts of the government which created protected areas such as Kings Park, Bold Park and other significant areas. Very few cities in the world have such large areas of natural bushland in the centre of a big city [67]. However, future urban growth will continue to put pressure on the biodiversity. If current policies (Perth and Peel@3.5million) are fully implemented, existing stocks of urban and urban deferred land would be consumed by about 2075 [68]. The challenge for urban planning to preserve urban forests and biodiversity is thus increasing, and it is clear that planning for future expansion should also include large protected areas.

Our MSPA output with spatial distribution of seven classes provides fundamental information for future urban planning. There is a need to maintain important green spaces which are classified as cores and bridges in the city, especially in the central region where most of the natural vegetation exists as islands. Also, the MSPA branch classes can be used to identify candidate ecological restoration areas. The branch class can be thought of [69] as a foundation of a potential corridor that could, if revegetated, connect two spatially disjunct core areas to improve connectivity in the larger region.

Although other analyses, such as functional connectivity, should be taken into account in landscape connectivity assessment [32], the structural connectivity analysis in this study will be useful for determining the priority protection level and critical areas of the connecting corridor, informing conservation strategies at a variety of scales, especially when the biodiversity values of this region are suffering from various threats including deforestation, feral animals, weed incursions, more frequent fires through arson and tree disease [64].

4.3 *The role of golf courses in maintaining urban forest*

Our study indicates that golf courses account for a significant proportion of the urban area of Perth. This category of land use has been vital in maintaining green space in urban areas over the past thirty years. In contrast to the overall decline in urban green space, golf courses have preserved green spaces within urban settings and even created a net gain of vegetation cover over time. The highest net gain was seen in the period between 1988 to 1998 when some golf courses were established resulting in the planting of trees.

In the green 'matrix' of Perth, golf courses with their significant area of vegetation cover have contributed considerably to the connectivity in the urban landscape. A significant proportion of their green space was classified as core or bridge categories. The proportion of vegetation within golf courses classified as bridges was higher than in the whole study area. Golf courses with large areas of native vegetation provide "links" to other large natural patches of urban vegetation.

Although there are concerns with the environmentally negative impacts of golf courses as a source of pollution through pesticide and fertiliser usage [22], habitat modification [25] and high water usage [70], previous studies provide evidence about the biological values of golf courses, such as providing refugial habitat for urban-avoiding wildlife [24, 71-79]. Our study indicates that golf courses in urban settings have been maintaining large green-area habitats and played an important role in biodiversity connectivity in the city.

4.4 *Monitoring urban forest dynamics*

In this study, we utilized medium-resolution satellite remote sensing data to identify land use classes, characterise vegetation dynamics and connectivity. The data maps the spatial and temporal patterns of land use types characterizing a consistent, detailed vegetation dynamic of the city [58, 80]. Clearly, the biophysical elements of urban landscapes are well-reflected through physical features (NDVI) derived from remote sensing data with an accuracy of up to 89%.

Despite these kinds of data, it is hard to describe the detailed information of ecosystem such as species composition and forest structure. However, they show their advantage in mapping land cover dynamics

across large areas of big cities over time when high resolution imagery is not available. For monitoring urban forests in big cities, the large scale and long temporal datasets are more advantageous compared with datasets that focus only on discerning a specific land use type in a relatively small area [27, 81, 82]. This is because it allows spatially detailed identification of changes associated with development over time. Therefore, the approach described in this paper provides baseline information for sustainable urban planning and development. In addition, the MSPA analysis can further evaluate the dynamic of vegetation cover by the describing the spatial configuration of ecosystems at the pixel level, detecting changes of habitat connectivity over time [32].

5 Conclusions

With rapid urban expansion, the most meaningful question to address is how to balance urban development and urban forest preservation. Urbanization requiring deforestation is inevitable in many cities worldwide. Our study found a significant loss of vegetation cover in a biodiversity hotspot over three decades of urbanization, which led to a reduction in habitat connectivity in the urban landscape. A lesson learned from the experience of urbanization in Perth is that any future urban growth following the patterns observed over the past three decades will continue to put pressure on maintaining urban forest ecosystems and biodiversity conservation. As cities continue to grow in response to socio-economic development, considering all opportunities for urban biodiversity conservation is important. Urban conservation strategies must therefore consider not only the protected areas, but also the off-reserve sites.

Our study indicates that golf courses in urban settings have been maintaining green-area habitats and have played an important role in biodiversity connectivity in the city. Potentially, urban golf courses could become more purposefully managed for biodiversity conservation and the improvement of critical ecosystem services in urban areas. In rapidly urbanizing biodiversity hotspots like Perth, where fragmentation is one the biggest threats to biodiversity, the way that golf courses contribute to increase the connectivity in the intervening urban matrix should not be underestimated. Therefore, it is important for government authorities and golf courses owners to pay more attention in maintaining ecosystem health in urban golf courses.

6 Acknowledgments

The authors thank Dr. Harry Eslick for his valuable advice in helping to design the research and Mr. Pham Dang Manh Hong Luan for his advice in data analysis. The research was supported by the Vietnamese Ministry of Education and Training and Murdoch University through a VIED-MU joint PhD scholarship to the first author.

7 References

1. Tabea TaE, Knop. A landscape ecology approach identifies important drivers of urban biodiversity. Global Change Biol. 2015;21 (4):1652-67.
2. Bagan H, Yamagata Y. Land-cover change analysis in 50 global cities by using a combination of Landsat data and analysis of grid cells. Environmental Research Letters. 2014;9(6):064015. doi: 10.1088/1748-9326/9/6/064015.
3. UNDESA UNDoEaSA. World urbanization prospects: The 2011 revision. United Nations Department of Economic and Social Affairs/Population Division, New York: 2012.
4. McGrane SJ. Impacts of urbanisation on hydrological and water quality dynamics, and urban water management: a review. Hydrological Sciences Journal. 2016;61(13):2295-311. doi: 10.1080/02626667.2015.1128084.
5. Gaston KJ, Ávila-Jiménez ML, Edmondson JL. REVIEW: Managing urban ecosystems for goods and services. Journal of Applied Ecology. 2013;50(4):830-40. doi: 10.1111/1365-2664.12087.
6. Declet-Barreto J, Brazel AJ, Martin CA, Chow WTL, Harlan SL. Creating the park cool island in an inner-city neighborhood: heat mitigation strategy for Phoenix, AZ. Urban Ecosystems. 2013;16(3):617-35. doi: 10.1007/s11252-012-0278-8.
7. Scott AA, Misiani H, Okoth J, Jordan A, Gohlke J, Ouma G, et al. Temperature and heat in informal settlements in Nairobi. PLOS ONE. 2017;12(11):e0187300. doi: 10.1371/journal.pone.0187300.
8. Van Den Berg AE, Custers MHG. Garndening promotes neuroendocrine and affective restoration from stress. Journal of Health Psychology. 2010;16(1):1359-053.
9. Mitchell R. Is physical activity in natural environments better for mental health than physical activity in other environments? Social Science & Medicine. 2013;91(Supplement C):130-4. doi: https://doi.org/10.1016/j.socscimed.2012.04.012.
10. Donovan GH, Butry DT, Michael YL, Prestemon JP, Liebhold AM, Gatziolis D, et al. The relationship between trees and human health: Evidence from the spread of the emerald ash borer. American Journal of Preventive Medicine. 2013;44(2):139-45. doi: https://doi.org/10.1016/j.amepre.2012.09.066.
11. Ulrich RS. View through a window may influence recovery from surgery. Science. 1984;224(4647):420.
12. Cecily Jane M. Promoting children's mental, emotional and social health through contact with nature: a model. Health Education. 2009;109(6):522-43. doi: 10.1108/09654280911001185.
13. Lee KE, Williams KJH, Sargent LD, Williams NSG, Johnson KA. 40-second green roof views sustain attention: The role of micro-breaks in attention restoration. Journal of Environmental Psychology. 2015;42(Supplement C):182-9. doi: https://doi.org/10.1016/j.jenvp.2015.04.003.
14. Kim G. Assessing Urban Forest Structure, Ecosystem Services, and Economic Benefits on Vacant Land. Sustainability. 2016;8(7):679. PubMed PMID: doi:10.3390/su8070679.
15. Zitkovic M. Managing green spaces for urban biodiversity. Boulevard Louis Schmidt 64 1040 Brussels, Belgium: Countdown 2010 Secretariat, IUCN Regional Office for Europe, 2008.

16. Seitz J, Escobedo F. Urban forests in Florida: Trees control stormwater runoff and improve water quality. University of Florida, Institute of Food and Agricultural Sciences. IFAS Extension Publication FOR184.2014.
17. Bailey D, Schmidt-Entling MH, Eberhart P, Herrmann JD, Hofer G, Kormann U, et al. Effects of habitat amount and isolation on biodiversity in fragmented traditional orchards. Journal of Applied Ecology. 2010;47(5):1003-13. doi: 10.1111/j.1365-2664.2010.01858.x.
18. Yu D, Liu Y, Xun B, Shao H. Measuring Landscape Connectivity in a Urban Area for Biological Conservation. CLEAN – Soil, Air, Water. 2015;43(4):605-13. doi: 10.1002/clen.201200448.
19. Bierwagen B. Connectivity in urbanizing landscapes: The importance of habitat configuration, urban area size, and dispersal2007. 29-42 p.
20. Lindenmayer D, Franklin J. Conserving forest biodiversity: a comprehensive multiscaled approach2002.
21. Napton DE, Laingen CR. Expansion of Golf Courses in the United States. Geographical Review. 2008;98(1):24-41.
22. Guzmán C, & Fernández, D.,Javier Mesa. Environmental impaccts by golf courses and strategies to minimize them: state of the art. International Journal of Arts & Sciences. 2014;7(3):403-17.
23. Colding J, Folke C. The Role of Golf Courses in Biodiversity Conservation and Ecosystem Management. Ecosystems. 2009;12(2):191-206. doi: 10.1007/s10021-008-9217-1.
24. Hodgkison SC, Hero JM, Warnken J. The conservation value of suburban golf courses in a rapidly urbanising region of Australia. Landscape and Urban Planning. 2007;79(3):323-37. doi: https://doi.org/10.1016/j.landurbplan.2006.03.009.
25. Terman MR. Natural links: naturalistic golf courses as wildlife habitat. Landscape and Urban Planning. 1997;38(3):183-97. doi: https://doi.org/10.1016/S0169-2046(97)00033-9.
26. Puglis HJ, Boone MD. Effects of Terrestrial Buffer Zones on Amphibians on Golf Courses. PLOS ONE. 2012;7(6):e39590. doi: 10.1371/journal.pone.0039590.
27. Theobald DM. Development and Applications of a Comprehensive Land Use Classification and Map for the US. PLOS ONE. 2014;9(4):e94628. doi: 10.1371/journal.pone.0094628.
28. Hu T, Yang J, Li X, Gong P. Mapping Urban Land Use by Using Landsat Images and Open Social Data. Remote Sensing. 2016;8(2):151. PubMed PMID: doi:10.3390/rs8020151.
29. Li X, Liu X, Gong P. Integrating ensemble-urban cellular automata model with an uncertainty map to improve the performance of a single model. International Journal of Geographical Information Science. 2015;29(5):762-85. doi: 10.1080/13658816.2014.997237.
30. Tucker CJ. Red and photographic infrared linear combinations for monitoring vegetation. Remote Sensing of Environment. 1979;8(2):127-50. doi: https://doi.org/10.1016/0034-4257(79)90013-0.
31. Myneni RB, Hall FG, Sellers PJ, Marshak AL. The interpretation of spectral vegetation indexes. IEEE Transactions on Geoscience and Remote Sensing. 1995;33(2):481-6. doi: 10.1109/TGRS.1995.8746029.
32. Vogt P, Riitters K, Estreguil C, Kozak J, G. Wade T, Wickham J. Mapping Spatial Patterns with Morphological Image Processing2007. 171-7 p.
33. Myers N, Mittermeier RA, Mittermeier CG, da Fonseca GAB, Kent J. Biodiversity hotspots for conservation priorities. Nature. 2000;403(6772):853-8. doi: 10.1038/35002501.
34. Kottek MG, Jürgen; Beck, Christoph; Rudolf, Bruno; Rubel, Franz. World Map of the K \ o ppen-Geiger climate classification updated. Meteorologische Zeitschrift. 2006;15(3):259-63. doi: 10.1127/0941-2948/2006/0130.
35. Hope PK, Drosdowsky W, Nicholls N. Shifts in the synoptic systems influencing southwest Western Australia. Climate Dynamics. 2006;26(7):751-64. doi: 10.1007/s00382-006-0115-y.
36. Mittermeier R, Gil P, Hoffmann M, Pilgrim J, Brooks T, Mittermeier C, et al. Hotspots Revisited. Earth's Biologically Richest and Most Endangered Terrestrial Ecoregions2004.
37. Environmental Protection Act 1986, (1986).

38. Environment Protection and Biodiversity Conservation Act 1999 (EPBC Act), (1999).
39. Australia GoW. Bush Forever Volume 1: Policies, principles and processes. In: Protection MfPaDoE, editor. Perth, WA.2000a.
40. Australia GoW. Bush Forever Volume 2: Directory of Bush Forever sites. In: Protection MfPaDoE, editor. Perth, WA.2000b.
41. Environment Dot. Shrublands and Woodlands of the eastern Swan Coastal Plain in Community and Species Profile and Threats Database. In: Environment Dot, editor. Canberra.2016.
42. Dhakal SP. Glimpses of sustainability in Perth, Western Australia: Capturing and communicating the adaptive capacity of an activist group. Consilience: The Journal of Sustainable Development. 2014;11(1):167–82.
43. Song C, Woodcock CE, Seto KC, Lenney MP, Macomber SA. Classification and Change Detection Using Landsat TM Data: When and How to Correct Atmospheric Effects? Remote Sensing of Environment. 2001;75(2):230-44. doi: https://doi.org/10.1016/S0034-4257(00)00169-3.
44. Cui L, Li G, Ren H, He L, Liao H, Ouyang N, et al. Assessment of atmospheric correction methods for historical Landsat TM images in the coastal zone: A case study in Jiangsu, China. European Journal of Remote Sensing. 2014;47(1):701-16. doi: 10.5721/EuJRS20144740.
45. Lu D, Mausel P, Brondizio E, Moran E. Assessment of atmospheric correction methods for Landsat TM data applicable to Amazon basin LBA research. International Journal of Remote Sensing. 2002;23(13):2651-71. doi: 10.1080/01431160110109642.
46. Gilmore S, Saleem A, Dewan A. Effectiveness of DOS (Dark-Object Subtraction) method and water index techniques to map wetlands in a rapidly urbanising megacity with Landsat 8 data. CEUR Workshop Proceedings. 2015;1323:100-8.
47. Nazeer M, Nichol J. Selection of atmospheric correction method and estimation of Chlorophyll-A (Chl-a) in coastal waters of Hong Kong. 3rd International Workshop on Earth Observation and Remote Sensing Applications, EORSA 2014 - Proceedings. 2014:374-8. doi: 10.1109/EORSA.2014.6927916.
48. Yusuf FR, Santoso KB, Ningam MUL, Kamal M, Wicaksono P. Evaluation of atmospheric correction models and Landsat surface reflectance product in Daerah Istimewa Yogyakarta, Indonesia. IOP Conference Series: Earth and Environmental Science. 2018;169:012004. doi: 10.1088/1755-1315/169/1/012004.
49. Dewi E, Trisakti B. COMPARING ATMOSPHERIC CORRECTION METHODS FOR LANDSAT OLI DATA. International Journal of Remote Sensing and Earth Sciences (IJReSES). 2017;13:105. doi: 10.30536/j.ijreses.2016.v13.a2472.
50. Mohajane M, Essahlaoui A, Oudija F, Hafyani ME, Hmaidi AE, Ouali AE, et al. Land Use/Land Cover (LULC) Using Landsat Data Series (MSS, TM, ETM+ and OLI) in Azrou Forest, in the Central Middle Atlas of Morocco. Environments. 2018;5(12):131. PubMed PMID: doi:10.3390/environments5120131.
51. Gu D, Gillespie A. Topographic Normalization of Landsat TM Images of Forest Based on Subpixel Sun–Canopy–Sensor Geometry. Remote Sensing of Environment. 1998;64(2):166-75. doi: https://doi.org/10.1016/S0034-4257(97)00177-6.
52. Herring JWaD. Measuring Vegetation (NDVI & EVI) 2000. Available from: https://earthobservatory.nasa.gov/features/MeasuringVegetation.
53. Cha S-Y, Park C-H. The utilization of google earth images as reference data for the multitemporal land cover classification with MODIS data of North Korea.2007.
54. Pascual-Hortal L, Saura S. Comparison and development of new graph-based landscape connectivity indices: towards the priorization of habitat patches and corridors for conservation. Landscape Ecology. 2006;21(7):959-67. doi: 10.1007/s10980-006-0013-z.
55. Saornil J, Gutiérrez J, Hernando A, Abril A, Sánchez B, Aparicio M. Structural connectivity as an indicator of species richness and landscape diversity in Castilla y León (Spain)2019. 286-97 p.
56. Wickham JD, Riitters KH, Wade TG, Vogt P. A national assessment of green infrastructure and change for the conterminous United States using morphological image processing. 2010.

57. Shi X, Qin M. Research on the Optimization of Regional Green Infrastructure Network. Sustainability. 2018;10(12):4649. PubMed PMID: doi:10.3390/su10124649.

58. MacLachlan A, Biggs E, Roberts G, Boruff B. Urban Growth Dynamics in Perth, Western Australia: Using Applied Remote Sensing for Sustainable Future Planning. Land. 2017;6(1):9. PubMed PMID: doi:10.3390/land6010009.

59. WorldPopulationReview. Perth Population 2019 2019 [21 February 2019]. Available from: http://worldpopulationreview.com/world-cities/perth-population/.

60. Commission WAP. Urban Growth Monitor Perth Methopolitan, Peel and Greater Bunbury Regions. In: WAPC WAPC, editor. Gordon Stephenson House 140 William Street Perth WA 6000: the Western Australian Planning Commission; 2019.

61. Authority MRP. The Corridor Plan for Perth. In: Authority MRP, editor. Perth, WA1970.

62. Development DoPaU. Metroplan: A planning strategy for the Perth Metropolitan Region. In: Development DoPaU, editor. Perth, WA.1990.

63. Bureau of Infrastructure TaREB. Population growth, jobs growth and commuting flows in Perth, . In: Transport DoIa, editor. Canberra ACT 2010.

64. Department of Biodiversity CaA. A methodology for the evaluation of wetlands on the Swan Coastal Plain, draft prepared by the Wetlands Section of the Department of Biodiversity. In: Regulation CaAatUWBotDoWaE, editor. Perth, WA2017.

65. Saura S, Vogt P, Velázquez J, Hernando A, Tejera R. Key structural forest connectors can be identified by combining landscape spatial pattern and network analyses. Forest Ecology and Management. 2011;262(2):150-60. doi: https://doi.org/10.1016/j.foreco.2011.03.017.

66. Morjan CL, Rieseberg LH. How species evolve collectively: implications of gene flow and selection for the spread of advantageous alleles. Molecular ecology. 2004;13(6):1341-56. doi: 10.1111/j.1365-294X.2004.02164.x. PubMed PMID: 15140081.

67. Green T. Perth's Urban Forest: A WA2.0 Project 2018.

68. Commission WAP. Perth and Peel @ 3.5 Million. In: Department of Planning GoWA, editor. Perth,WA, Australia2015.

69. Wickham J, Riitters K, Vogt P, Costanza J, Neale A. An inventory of continental U.S. terrestrial candidate ecological restoration areas based on landscape context2017.

70. Neylan J. Improving The Environmental Management of New South Wales Golf courses. In: NSW DoEaCC, editor. 59–61 Goulburn Street, Sydney, PO Box A290.

71. Deslauriers MR, Asgary A, Nazarnia N, Jaeger JAG. Implementing the connectivity of natural areas in cities as an indicator in the City Biodiversity Index (CBI). Ecological Indicators. 2017. doi: https://doi.org/10.1016/j.ecolind.2017.02.028.

72. Dobbs EK, Potter DA. Naturalized habitat on golf courses: source or sink for natural enemies and conservation biological control? Urban Ecosystems. 2016;19(2):899-914. doi: 10.1007/s11252-015-0521-1.

73. Saarikivi J, Tähtinen S, Malmberg S, Kotze DJ. Converting land into golf courses – effects on ground beetles (Coleoptera, Carabidae). Insect Conservation and Diversity. 2015;8(3):247-51. doi: 10.1111/icad.12103.

74. Chester ET, Robson BJ. Anthropogenic refuges for freshwater biodiversity: Their ecological characteristics and management. Biological Conservation. 2013;166(Supplement C):64-75. doi: https://doi.org/10.1016/j.biocon.2013.06.016.

75. Saarikivi J, Idström L, Venn S, Niemelä J, Kotze DJ. Carabid beetle assemblages associated with urban golf courses in the greater Helsinki area. European Journal of Entomology. 2010;107(4):553-61. PubMed PMID: 814377100.

76. Hudson M-AR, Bird DM. Recommendations for design and management of golf courses and green spaces based on surveys of breeding bird communities in Montreal. Landscape and Urban Planning. 2009;92(3):335-46. doi: https://doi.org/10.1016/j.landurbplan.2009.05.017.

77. Colding J, Lundberg J, Lundberg S, Andersson E. Golf courses and wetland fauna. Ecological Applications. 2009;19(6):1481-91. doi: 10.1890/07-2092.1.

78. Burgin S, Wotherspoon D. The potential for golf courses to support restoration of biodiversity for BioBanking offsets. Urban Ecosystems. 2009;12(2):145-55. doi: 10.1007/s11252-008-0076-5.
79. Tanner RA, Gange AC. Effects of golf courses on local biodiversity. Landscape and Urban Planning. 2005;71(2):137-46. doi: https://doi.org/10.1016/j.landurbplan.2004.02.004.
80. Angiuli E, Trianni G. Urban Mapping in Landsat Images Based on Normalized Difference Spectral Vector. IEEE Geoscience and Remote Sensing Letters. 2014;11(3):661-5. doi: 10.1109/LGRS.2013.2274327.
81. Wu S-S, Qiu X, Usery EL, Wang L. Using Geometrical, Textural, and Contextual Information of Land Parcels for Classification of Detailed Urban Land Use. Annals of the Association of American Geographers. 2009;99(1):76-98. doi: 10.1080/00045600802459028.
82. Toole JL, Ulm M, González MC, Bauer D, editors. Inferring land use from mobile phone activity. Proceedings of the ACM SIGKDD international workshop on urban computing; 2012: ACM.

Figure 1

Click here to access/download;Figure;Fig1.tif

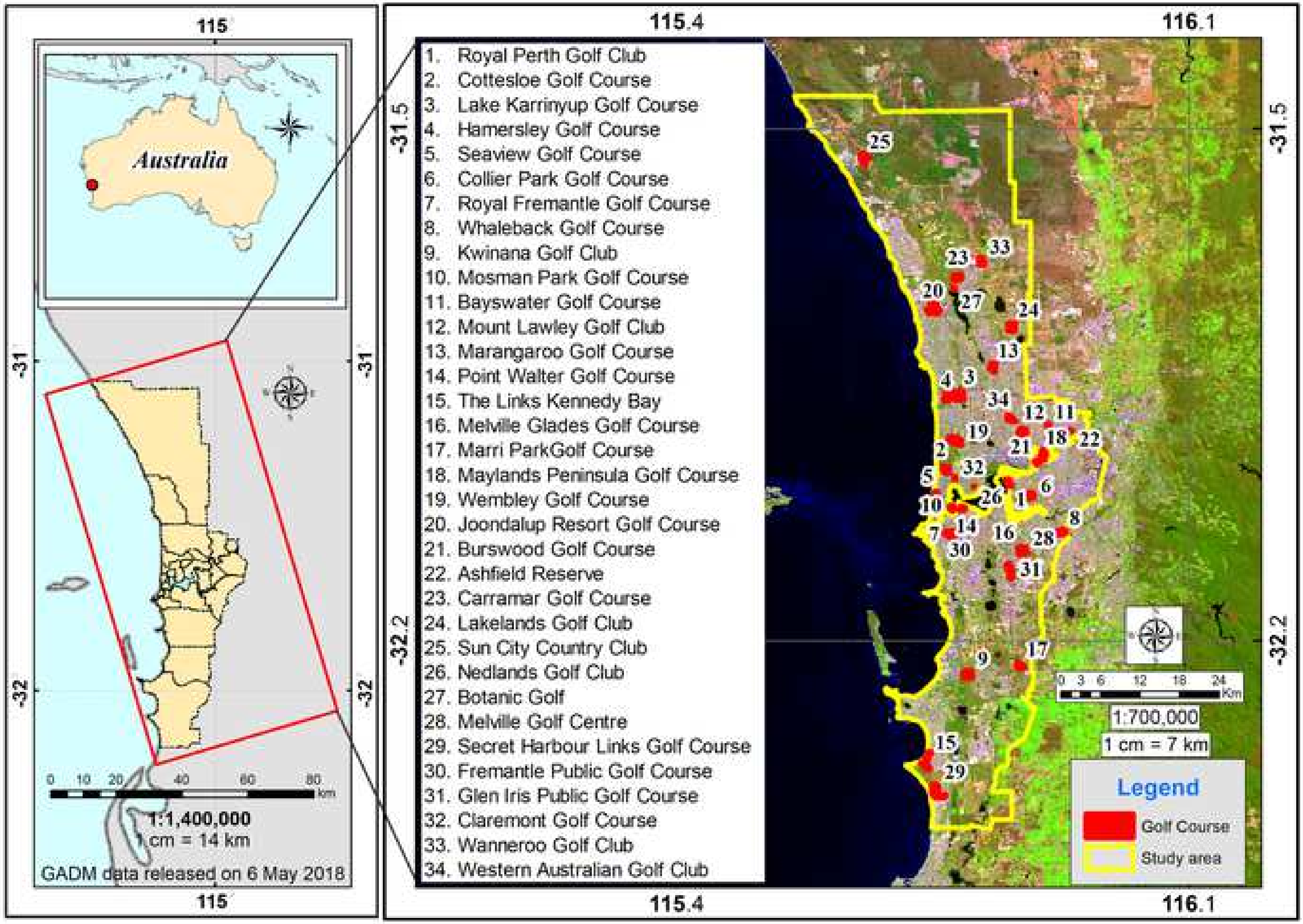

 Click here to access/download;Figure;Fig2.tif

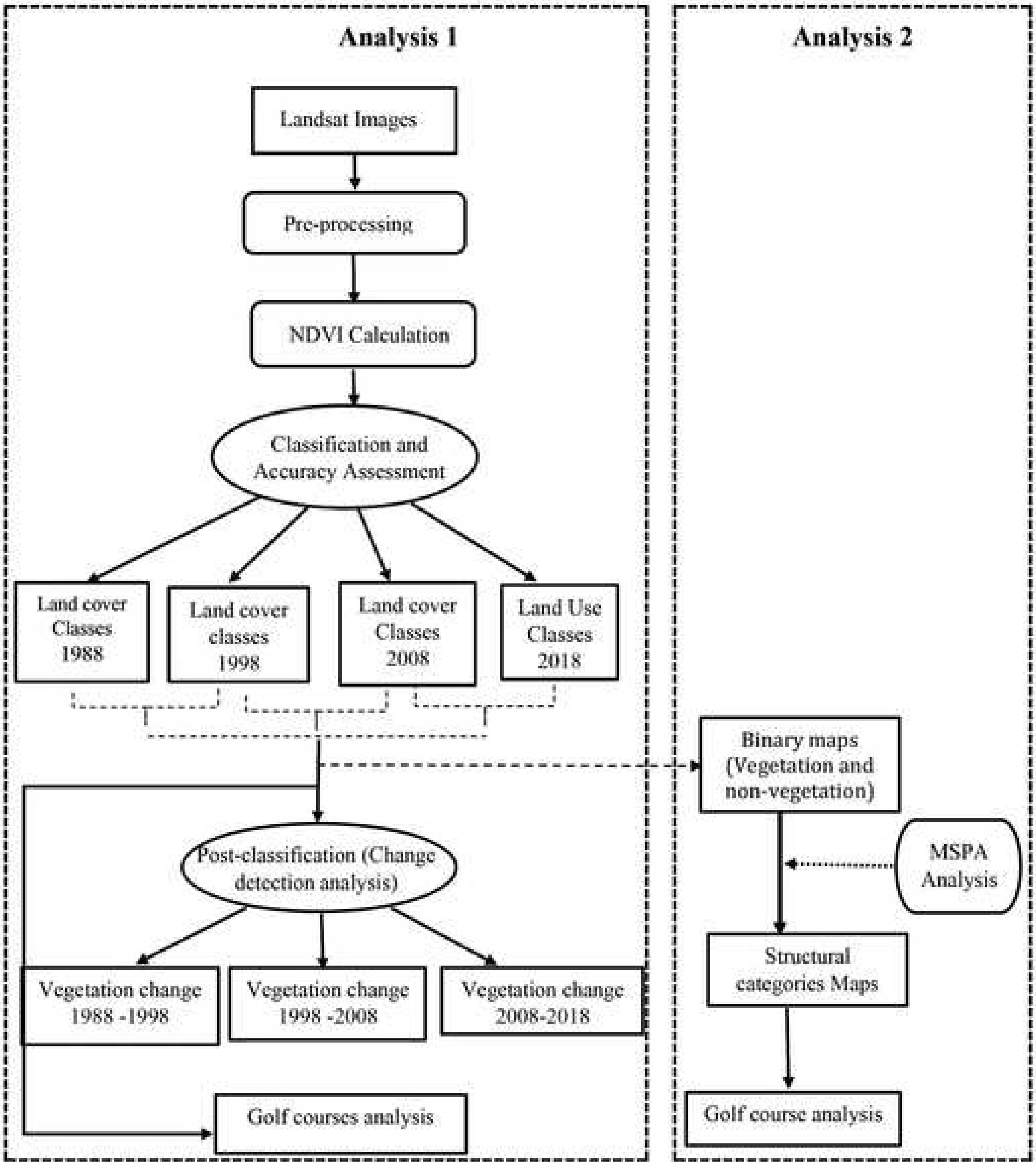

Figure 3

Click here to access/download;Figure;Fig3.tif

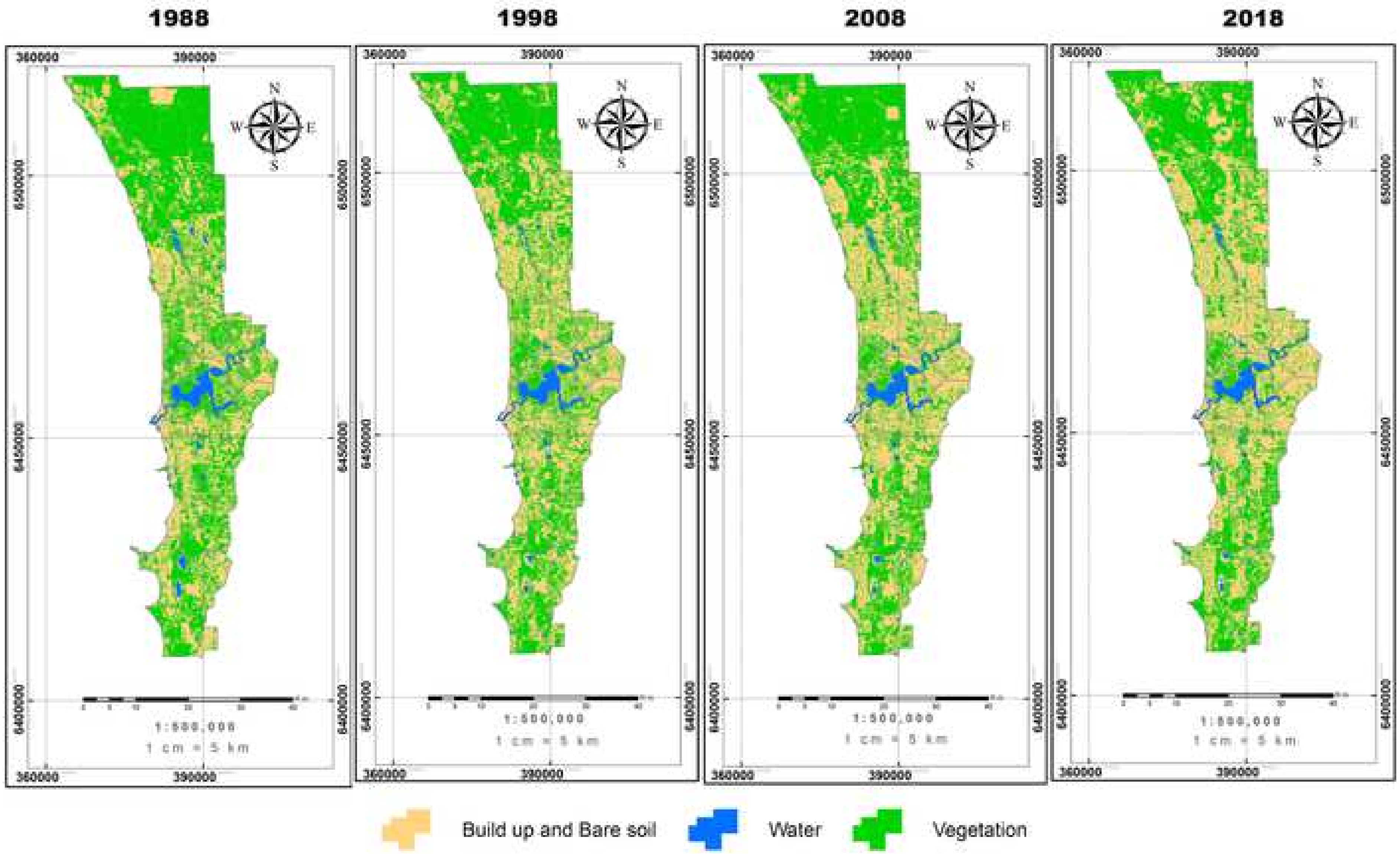

Figure 4

Click here to access/download;Figure;Fig4.tif

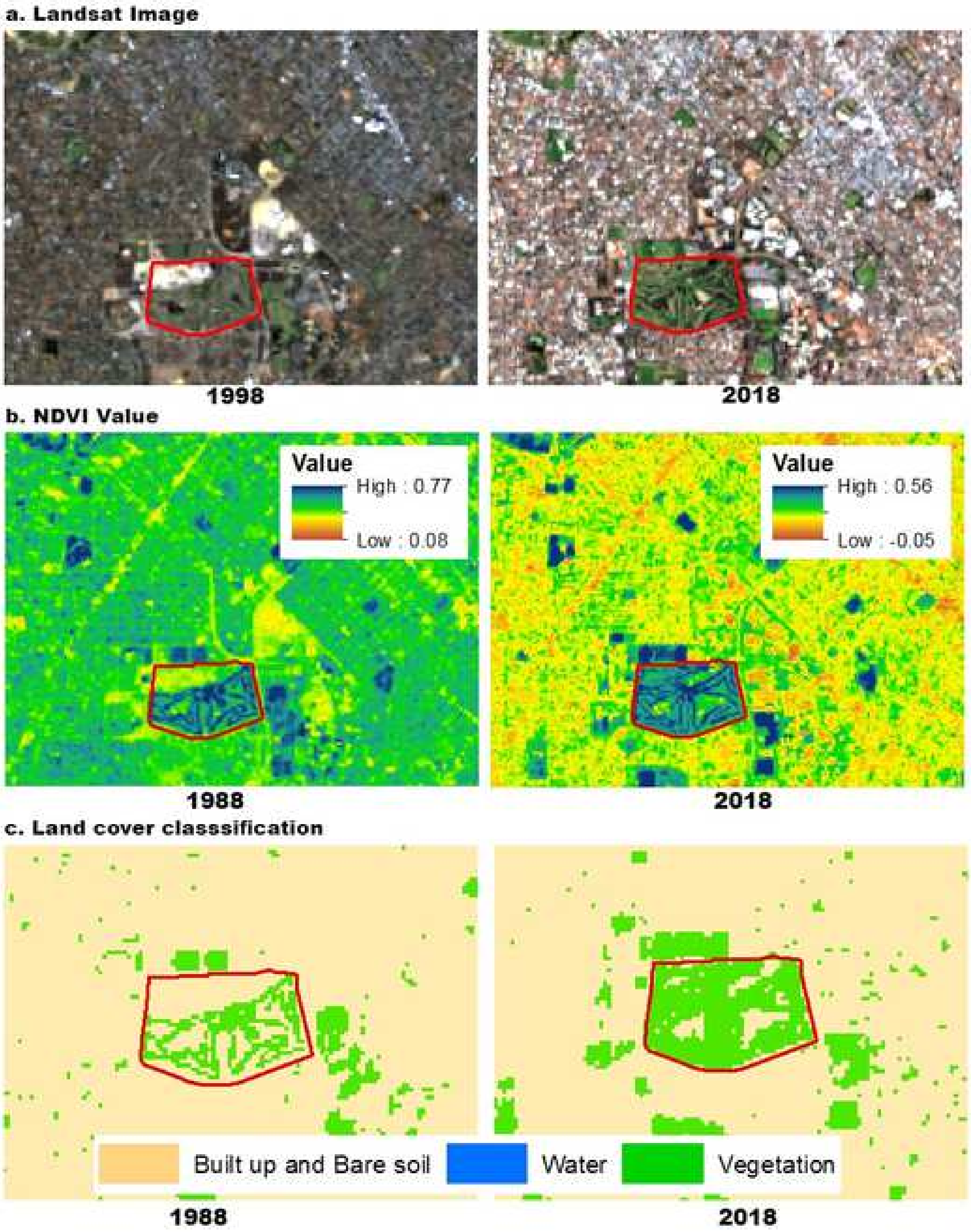

Figure 5

Click here to access/download;Figure;Fig5.tif

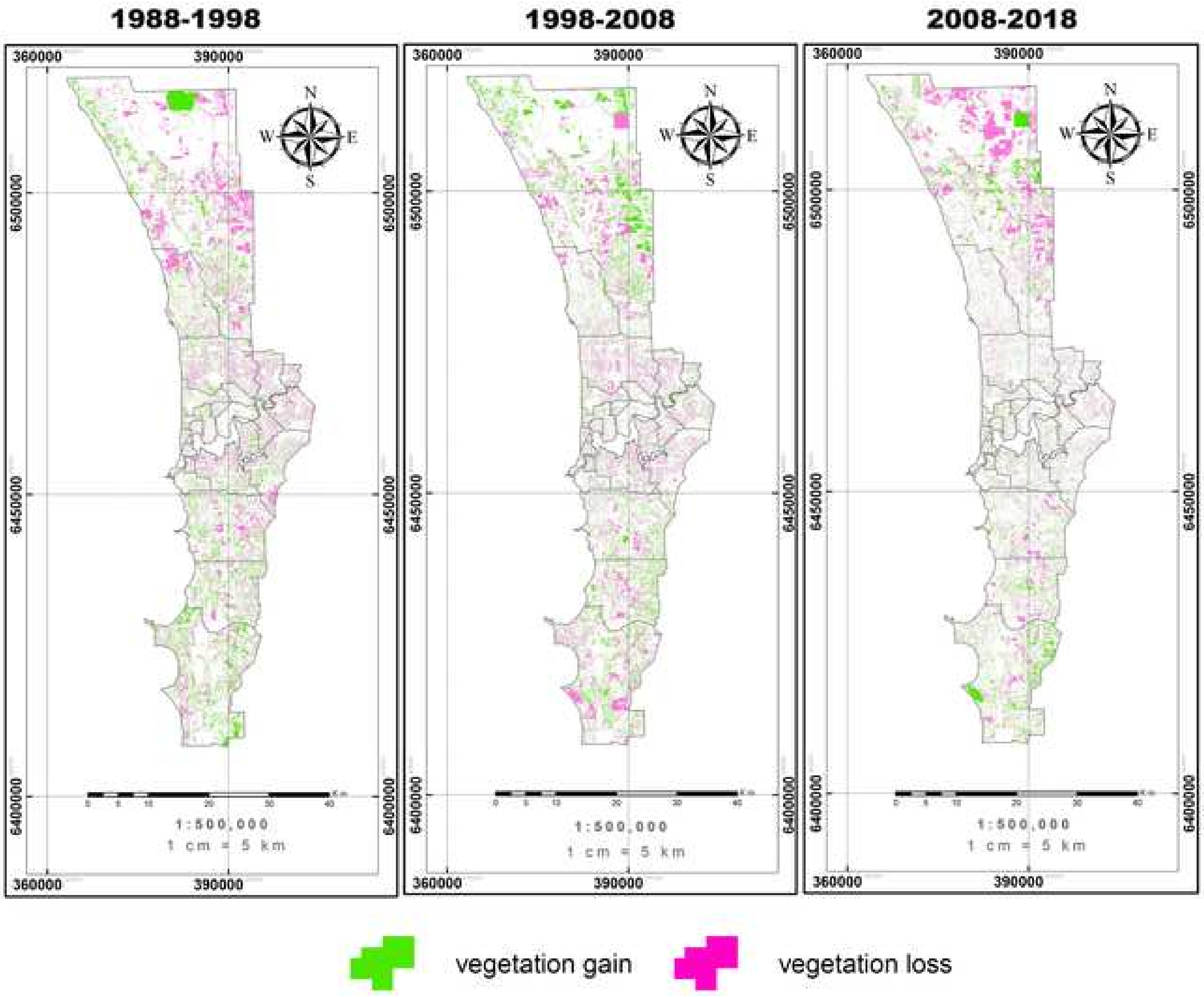

Figure 6

Click here to access/download;Figure;Fig6.tif

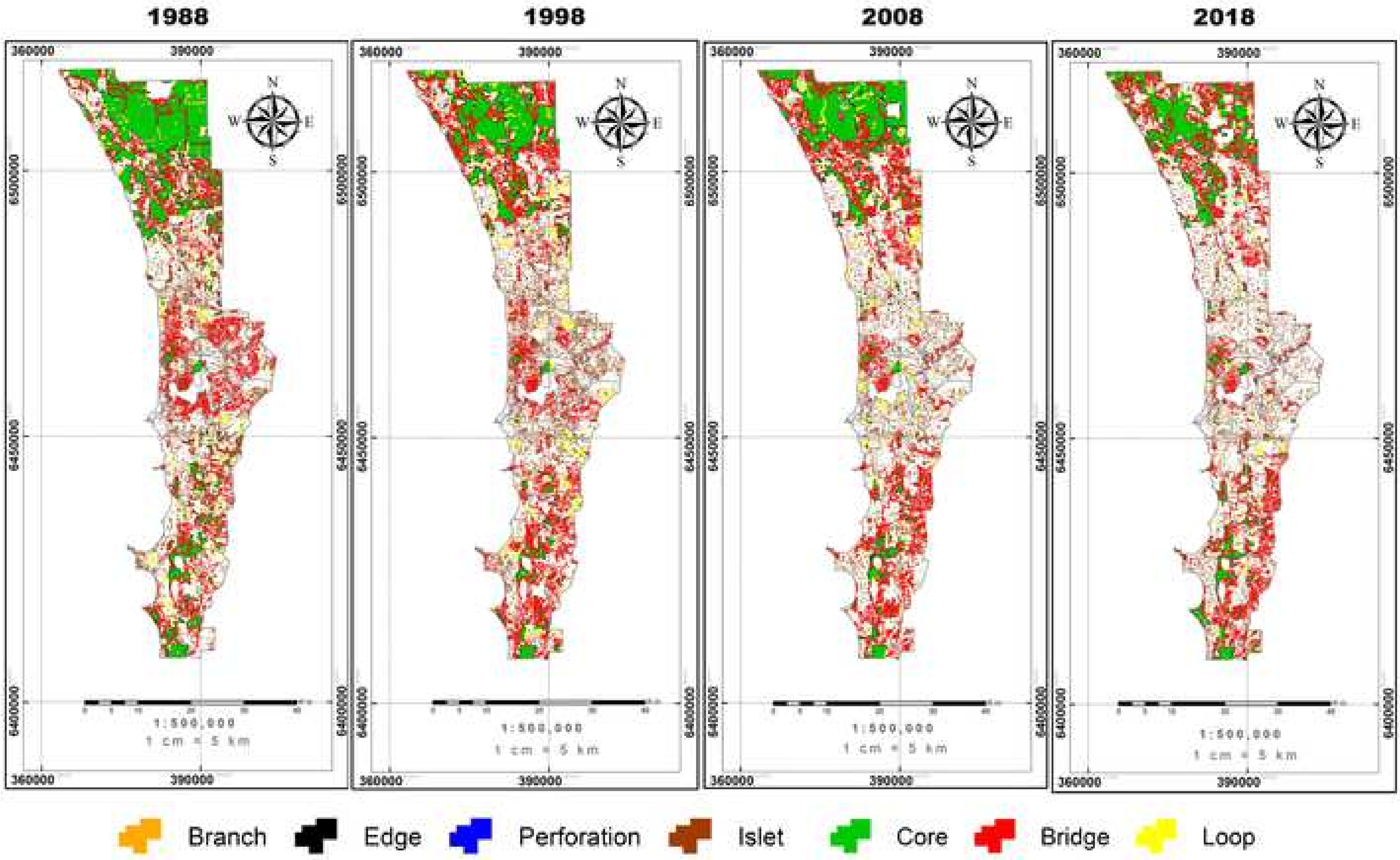

Vegetation trends associated with urban development: ~~T~~the role of golf courses~~Trends in vegetation dynamics associated with urban development: The role of golf courses~~

Thu Thi Nguyen[1,3]*, Paul Barber[2,3], Richard Harper[3], Tran Vu Khanh Linh[4], Bernard Dell[3]

[1] Vietnam National University of Forestry (VNUF), Xuan Mai, Chuong My, Ha Noi, Vietnam,
[2] ArborCarbon Pty Ltd, 1 City Farm Place, East Perth, WA 6004, Australia

[3] Agricultural and Forestry Sciences, Murdoch University, Murdoch, WA 6150, Australia~~,~~

[4] Faculty of Forestry, Nong Lam University - Ho Chi Minh City, Thu Duc District, Ho Chi Minh City, Vietnam~~.~~

*Corresponding author

Emails: Thu.Nguyen@murdoch.edu.au; thu.nguyen.2k14@gmail.com

Abstract

Globally, cities ~~have~~ are growing rapidly in size and density and this has caused profound impacts on urban forest ecosystems. Urbanization requiring ~~clearing~~ deforestation ~~of vegetation~~ reduces ecosystem services that benefit both city dwellers and biodiversity. Understanding spatial and temporal patterns of vegetation changes associated with urbanization is thus a vital ~~for~~ component of future sustainable urban development. We used Landsat time series data for ~~the~~ three decades from ~~period~~ 1988 to 2018 to characterize changes in vegetation cover and habitat connectivity in the Perth Metropolitan Area, in a rapidly urbanising Australian biodiversity hotspot, as a case study to understand the impacts of urbanization on urban forests. Moreover, as golf courses are ~~rapidly increasing~~ a major component in ~~many~~ urban areas, we assessed the role of golf courses in maintaining vegetation cover and creating habitat connectivity. ~~We~~ To do this we employed (1) land use classification with~~,~~ post-classification change detection, and (2) Morphological Spatial Pattern Analysis (MSPA). Over 17,000 ~~hectares~~ ha of vegetation were cleared and the area of vegetation

Style Definition: Normal: Font: Times, Line spacing: Double
Style Definition: Heading 1: Font: Bold, Font color: Text 1, Outline numbered + Level: 1 + Numbering Style: 1, 2, 3, … + Start at: 1 + Alignment: Left + Aligned at: 0" + Indent at: 0.3"
Style Definition: Heading 2: Font: Bold, Italic, Outline numbered + Level: 2 + Numbering Style: 1, 2, 3, … + Start at: 1 + Alignment: Left + Aligned at: 0" + Indent at: 0.4"
Style Definition: Heading 3: Font: 12 pt, Not Bold,
Formatted: Font: 14 pt, Bold
Formatted: Font: 14 pt
Formatted: Font: 14 pt, Bold
Formatted: Font: Times, 14 pt
Formatted: Font: 11 pt
Formatted: Font: Times, 11 pt, Not Bold
Formatted: Font: +Body (Calibri), 11 pt
Formatted: Font: Times, 11 pt, Not Bold
Formatted: Font: (Default) Times, 11 pt, Not Bold, Font color: Auto, Superscript
Formatted: Font: (Default) Times, 11 pt, Font color: Auto
Formatted: Font: (Default) Times, 11 pt, Not Bold, Font color: Auto, Superscript
Formatted: Font: (Default) Times, 11 pt, Font color: Auto
Formatted: Font: (Default) Times, 11 pt, Font color: Auto, Superscript
Formatted: Font: (Default) Times, 11 pt, Font color: Auto
Formatted: Font: (Default) Times, 11 pt, Not Bold, Font color: Auto, Superscript
Formatted: Font: (Default) Times, 11 pt, Font color: Auto
Formatted: Justified
Formatted: Font: Times, 11 pt
Formatted: Font: Times, 11 pt
Field Code Changed
Formatted: Font: Times, 11 pt
Formatted: Font: (Default) Times, 12 pt, Font color: Auto
Formatted: Normal
Formatted: Font: (Default) Times, 12 pt
Formatted ...

contributing to biodiversity connectivity was reduced significantly over the three decades. The spatial patterns of vegetation loss and gain were different in each of ~~last~~the three decades (~~–~~1988-2018), ~~the three decades~~ reflecting the implementation of urban planning. Furthermore, MSPA analysis showed that the reduction in vegetation cover led to habitat fragmentation with a significant decrease in the core and bridge classes and an increase in isolated patches in the urban landscape. Golf courses played a useful role in maintaining vegetation cover and contributing to connectivity in a regional biodiversity hotspot. Our findings suggest that for future urban expansion, urban planning needs to more carefully consider ~~land clearing and its~~the impacts of deforestation on connectivity in the landscape. Moreover, there is a need to take into consideration opportunities for off-reserve conservation in smaller habitat fragments such as in golf courses in sustainable urban management.

1 Introduction

Formatted: Font: (Default) Times, 11 pt, Font color: Auto, English (Australia)

Formatted: Font: (Default) Times, 11 pt, Font color: Auto, English (Australia)

Formatted: Font: (Default) Times, 11 pt, Font color: Auto, English (Australia)

Formatted: Font: (Default) Times, 11 pt

Formatted: Not Highlight

Formatted: Font: (Default) Times, 11 pt

Formatted: Not Highlight

Formatted: Font: (Default) Times, 11 pt

Formatted: Font: (Default) Times, 12 pt, Not Bold, Not Italic

~~—~~Introduction

Globally, cities have grown rapidly in number and size over recent decades [1, 2]. This trend is predicted to continue as urban areas are expected to absorb most ~~of the~~ global population growth [3]~~-~~. While the process of urbanization presents key implications for changes in physical landscapes and demographic characteristics, it can cause profound impacts on environmental components, especially on urban forest ecosystems [4, 5].

Vegetation in urban landscapes is critically important because ~~it can~~ provides goods and services, and full ecosystem functions that benefit city dwellers and the environment. On the one hand, a remarkable range of human well-being benefits are derived~~delivered~~ from urban green spaces including mitigating the urban heat island (UHI) effect which is a threat to human health [6, 7]; reducing stress [8], ~~risk of poor mental health [9] and mortality from cardiovascular disease [10]; and~~ improving healing times [11], increasing self-esteem and empowerment [12], and improving cognitive ability [13]. On the other hand, urban green spaces provide various ecosystem services such as strengthening resistance to some kinds of natural disasters~~,~~ for example, floods ~~natural disasters~~ [14], promoting biological processes such as pollination [15], and reducing surface erosion from stormwater runoff [16]. The amount of vegetation in cities strongly influences biodiversity, especially where vegetation is set aside during the process of urbanization [17]. However, if species dispersal and exchange among these patches is insufficient to allow gene flow and diversity, loss of regional biodiversity is inevitable [18]. Therefore, urban development that requires ~~clearing of vegetation~~deforestation, ~~due to~~ ~~causes reduction in vegetation cover,~~with habitat loss and fragmentation is ~~, and hence may be considered~~ a threat to biodiversity [17, 19].

Urban conservation strategies must therefore consider not only the size and quality of habitat reserves, but the connectivity in the intervening urban vegetation matrix [19]. While the need to protect large habitat reserves is obvious, opportunities for off-reserve conservation of smaller habitat fragments should not be overlooked [20]. Understanding spatial and temporal patterns of vegetation change associated with urbanization, as well as opportunities for the preservation of green spaces outside

Formatted: Font: (Default) Times, 12 pt, Not Bold

Formatted: Font: (Default) Times, 12 pt, Font color: Text 1

Formatted: Check spelling and grammar

Formatted: Left, Space Before: 0 pt, After: 0 pt, Line spacing: single

Field Code Changed

Formatted: Font: 11 pt

Formatted: Default Paragraph Font, Font: 11 pt

Field Code Changed

Formatted: Font: 11 pt

Formatted: Default Paragraph Font, Font: 11 pt

Formatted: Check spelling and grammar

Formatted: Font: (Default) Times, 11 pt, Font color: Auto, English (Australia)

Field Code Changed

Formatted: Font: 11 pt

Formatted: Default Paragraph Font, Font: 11 pt

Formatted: Check spelling and grammar

Formatted: Check spelling and grammar

Formatted: Check spelling and grammar

Formatted: Check spelling and grammar

Formatted: Not Expanded by / Condensed by , Pattern: Clear

Formatted: Font color: Auto, Pattern: Clear

Formatted: Font color: Auto, Pattern: Clear

natural reserves, is vital for future sustainable urban development especially in areas of global ecological importance.

As the number of golf courses is rapidly increasing in many urban areas worldwide [21], there have been many environmental arguments about this green space category in urban landscapes. ~~,~~Golf courses are sometime considered to be major polluters of the environment through pesticide and fertilizer use [22]. ~~However, golf courses in urban settings create large green-area habitats, which even surpass many nature reserves in size [23].~~ In fact, golf courses have been established for recreational purposes, which are a mix of bushland, fairways and infrastructure. Though they are not fully ecologically functional as with a natural parks, previous studies investigated the condition of vegetation inside the golf courses and indicated that the bushland in non-playing areas of golf courses are significantly important to biodiversity conservation and the provision of ecosystem services in cities [24], such as providing refuge habitats for urban-avoiding wildlife [23, 25, 26]. Therefore, potentially, together with the natural reserves, golf courses can play some roles as off-reserve sites for purposeful biodiversity conservation in urban landscapes. ~~Potentially, urban golf courses could become more purposeful areas for biodiversity conservation and the provision of ecosystem services in cities [24]. Previous studies have identified some of the biological values of golf courses, such as providing refuge habitats for urban-avoiding wildlife [23, 25, 26].~~ Nevertheless, little research has been undertaken to comprehensively assess the role of golf courses in maintaining vegetation patches as interconnected nodes in urban landscapes during a long period of urban development where ~~vegetation clearing~~deforestation was significant.

~~Vegetation clearing~~Deforestation for urban expansion can occur gradually over multiple years. Satellite-based remote sensing holds certain advantages in the characterization of these changes in urban landscapes because of the large spatial coverage, high time resolution, and wide availability of data [27]. For example, medium resolution L~~L~~andsat images ~~of medium-resolution~~ allow ~~for~~ mapping of large urban areas ~~at a large spatial scale~~ [28]. Many methods have been used to detect, monitor and quantify vegetation changes, but differences in vegetation index and land use classification are the most widely used methods for vegetation change over a long period of time [28].

Formatted: Font: (Default) Times New Roman

Formatted: Left, Space Before: 0 pt, After: 0 pt, Line spacing: single

Formatted: Font: (Default) Times New Roman

Formatted: Font: (Default) Times New Roman

Formatted: Font: Times New Roman

Formatted: Font: Times New Roman

Formatted: Font: (Default) Times New Roman

Formatted: Font: (Default) Times, 11 pt, Font color: Auto

Formatted: Font: (Default) Times, 11 pt, Font color: Auto

Formatted: Font: (Default) Times, 11 pt, Font color: Auto

Formatted: Font: (Default) Times, 11 pt, Font color: Auto

Formatted: Font: (Default) +Body (Calibri)

A great number of vegetation indices have been proposed, ranging from very simple to very complex band combinations [29]. The most widely-used vegetation index is the Normalized Difference Vegetation Index (NDVI), ~~,~~ [29]which is the most efficient and simple metric to identify vegetated areas and their condition [30]. ~~, which~~This separates green vegetation from other surfaces based on the ability of chlorophyll to absorb red light for photosynthesis and reflect the near-infrared (NIR) wavelengths [31]. ~~.~~ Furthermore, information on vegetation cover dynamics can be combined with Morphological Spatial Pattern Analysis (MSPA) to describe the spatial configuration of the ecosystem at the pixel level, making it possible to detect temporal changes in the structural connectivity of habitats in urban settings [32].

Therefore, to enhance the understanding of vegetation dynamics associated with urbanization and the role of golf courses in maintaining urban forests, we used Landsat imagery to map vegetation cover and to assess its spatial and temporal distribution over three decades from 1988 to 2018. ~~Then, m~~Maps of vegetation cover were used for MSPA analysis to detect changes in habitat connectivity. We chose the Perth Metropolitan area for the study as it lies within a rapidly urbanizing biodiversity hotspot in Australia. The three primary research objectives were to: (1) determine the spatial and temporal patterns of ~~vegetation clearing~~deforestation in urbanization; (2) evaluate the spatial and temporal patterns of green landscape connectivity; and (3) assess the ~~impact~~ role of golf courses ~~on~~ in preserving green spaces and biodiversity in an urban landscape. This analysis will provide a useful perspective on the land-use pressure facing vegetation remnants ~~in~~ in this ~~the~~ region ~~and their connectivity in the Southwest Australia Ecoregion (SWAE)~~ which is recognised as one of 35 international biodiversity hotspots~~, and is home to~~ with over 1500 plant species with a high degree of endemism [33], and provide a framework for planning urban expansions both in this region and globally. ~~.~~

2 Methods

2.1 Study area

The study area belongs to the Perth Metropolitan ~~area~~ region covering four sub-regions (North West, Middle Central, Inner Central and South West). Perth has a Mediterranean-type temperate climate

Formatted: Font: (Default) Times, 11 pt, No underline, Font color: Auto, Not Highlight

Formatted: Left, Space Before: 0 pt, After: 0 pt, Line spacing: single, Adjust space between Latin and Asian text, Adjust space between Asian text and numbers

Formatted: Font: (Default) Times, 11 pt, Not Highlight

Formatted: Font: 11 pt, English (Australia)

Formatted: Font: 11 pt, English (Australia)

Formatted: Default Paragraph Font, Font: 11 pt

Formatted: Font: Not Bold

Formatted: Line spacing: single

Formatted: Heading 2, Space Before: 0 pt, After: 0 pt, Line spacing: single

Formatted: Left, Line spacing: single

with a hot and dry summer, and a cold and rainy season ~~occuring~~occurring between May and October [34]. ~~West) (Fig 1).~~ U[34]nder future climate-change scenarios, this area is projected to experience a lower annual rainfall ~~in the future~~ [35].

~~Although Perth may be the driest regions on Earth, it is still home to some amazing biodiversity.~~ Perth belongs to the Australia's southwest corner, which is recognized as a global biodiversity "hotspot" with outstanding natural environments. Our study area ~~covers~~occurs on ~~most of~~ the Swan C~~c~~oastal P~~p~~lain~~. This plain~~ which is ~~connected with the valleys around Perth, the Esperance plains, and the jarrah-karri eucalyptus forests, making~~part of the southwest of Australia which in turn has the highest concentration of rare and endangered species on the entire continent (at least 1,500 endemic species). More than 6,000 species of native plants and 100 native mammals, birds, frogs and reptiles ~~found~~occur in this region, making it a biodiversity "hotspot" [33, 36].

Perth has retained a significant area of natural vegetation which ~~have~~has conservation significance thanks to the introduction of ~~some of~~ legislation~~,~~ and policies aimed primarily at protecting biophysical environmental values. ~~Some of those policies are~~This includes , the Western Australian *Environmental Protection Act* 1986 [37]; the Federal Government's *Environment Protection and Biodiversity Conservation Act* 1999 [38]; and more recently, Bush Forever [39, 40], a policy which took a whole-of-government approach to identify and protect biologically significant bushland and wetlands within the Perth metropolitan area. However, Perth ~~Perth is located in a globally recognised biodiversity hotspot~~ [41] ~~in a region of Mediterranean-type climate and~~ has experienced extensive urban development ~~at an unsustainable rate~~since the 1980s [42]. The expansive growth of the Perth metropolitan footprint has contributed to the loss of biodiversity, together with the ecosystem services provided by natural areas.[37-40].

Together with urbanization, the golf industry has expanded contributing to a growing proportion of Perth's urban green space~~. There are~~ with 34 golf courses in the study area (~~Fig~~Figure 1).

Formatted: English (Australia)
Formatted: English (Australia)
Formatted: English (Australia)
Formatted: English (Australia)
Formatted: English (Australia)
Formatted: English (Australia)
Formatted: English (Australia)
Formatted: English (Australia)
Formatted: English (Australia)
Formatted: English (Australia)
Formatted: English (Australia)
Formatted: English (Australia)
Formatted: English (Australia)
Formatted: English (Australia)
Formatted: English (Australia)
Formatted: English (Australia)
Formatted: English (Australia)
Formatted: Font: Italic, English (Australia)
Formatted: English (Australia)
Formatted: Font: Italic, English (Australia)
Formatted: English (Australia)
Formatted: Font: Italic, English (Australia)
Formatted: English (Australia)
Formatted: English (Australia)
Formatted: Font: (Default) Times, 11 pt, Highlight
Formatted: English (Australia)
Formatted: Font: +Body (Calibri), English (Australia)
Formatted: Font: Not Bold

Figure 1. Location of the s~~S~~**tudy area** (from USGS EROS (Earth Resources Observatory and Science (EROS) Center) in the public domain: http://eros.usgs.gov/#)

2.2 Approaches

We conducted two types of analyses in this study: (1) assessment of vegetation cover change in golf courses since 1988 relative to surrounding areas to provide a broad overall context of the urban vegetation changes that have taken place throughout the region and the role of golf courses; and (2) assessment of MSPA. In these analyses, we used four data sets (1988, 1998, 2008, 2018) covering thirty years of urban development. The steps taken in this study are summarized in a flow chart (~~Fig~~Figure 2).

2.2.1 Landsat data and pre-processing

Three Landsat 5 Thematic Mapper images (WRS path 113 row 82) were acquired from 1988 to 2008 and one Landsat OLI 8 (WRS path 113 row 82) was acquired in 2018 at four time steps, including ~~(~~year 1988 (11th December), year 1998 (7th December), year 2008 (18th December), year 2018 (14th December). The Landsat imagery were obtained from the US Geological Survey (USGS) of the Earth Resources Observation and Science Center (EROS). ~~Earth Resources Observation~~. Image dates were selected ~~from cloud-free scenes~~ acquired during December (summer, dry season) to reduce the seasonal difference effects ~~(Ttable 1)~~. All images selected are cloud-free scenes or little cloud cover (0.05% and 0.4%) scenes with the whole study area is cloud-free; Therefore, is no requirement for removing cloud. Georeferencing was performed at the USGS prior to downloading the data (L1T level of systematic geometric accuracy) and no further refinement was undertaken. Atmospheric and topographic corrections were performed on the Landsat data sets. The atmospheric correction was carried out to adjust the multitemporal dataset to a common radiometric scale [43].

~~**Table 1. Landsat scenes used in the study**~~

Formatted: Left, Line spacing: single, Tab stops: 1.25", Left

Formatted: Font: +Body (Calibri), Bold

Formatted: Left, Line spacing: single

Formatted: Left: 0.98", Right: 0.98", Header distance from edge: 0.49", Footer distance from edge: 0.49", Different first page header

Formatted: Left, Line spacing: single

Formatted: Font: (Default) Times, 12 pt

Formatted: Font: (Default) Times, 12 pt, Not Bold

Formatted: Heading 2, Space Before: 0 pt, After: 0 pt, Line spacing: single

Formatted: Left, Space Before: 0 pt, After: 0 pt, Line spacing: single, Adjust space between Latin and Asian text, Adjust space between Asian text and numbers

Formatted: Font: 12 pt, Not Bold, Font color: Auto

Formatted: Superscript

Formatted: Superscript

Formatted: Superscript

Formatted: Superscript

Formatted: Font: (Default) Times, 11 pt, English (Australia)

Formatted: Font color: Auto

Formatted: Font color: Auto

Formatted: Font: (Default) Times, Bold

Formatted: Left

Formatted: Font: Bold, Pattern: Clear

Formatted: Font: (Default) +Body (Calibri), 11 pt, Bold

Type of imagery	Year	Day/month	Cloud Cover	Resolution (m)
Landsat 5 TM	1988		0%	30m
	1998		0%	30m
	2008		0%	30m
Landsat 8 OLI	2018		0%	30m

The first process of atmospheric correction was conversion of the digital number (DN) remote sensing data values to at-sensor radiance based on the image header file. After that we employed the image-based models - dark object subtraction (DOS) to correct atmospheric scattering scene-by-scene. tThis method is a widely used and effective method in atmospheric correction [44-50]. Topographic correction was conducted to remove the topographic effects. We used a sun-canopy-sensor (SCS) correction based on the 30 m digital elevation model (DEM) because topographic shading is not only due to slope but also to shadowing of one tree crown over another and this is one of the most widely and effective used methods of topographic correction [51].

Formatted: Font: Bold

Formatted: Don't adjust space between Latin and Asian text, Don't adjust space between Asian text and numbers

Commented [RH1]: Add data in this column

Formatted Table

Formatted: Left, Space Before: 0 pt, After: 0 pt, Line spacing: single, Adjust space between Latin and Asian text, Adjust space between Asian text and numbers

Formatted: Pattern: Clear

Formatted: Font: 11 pt

Figure 2.

Flow

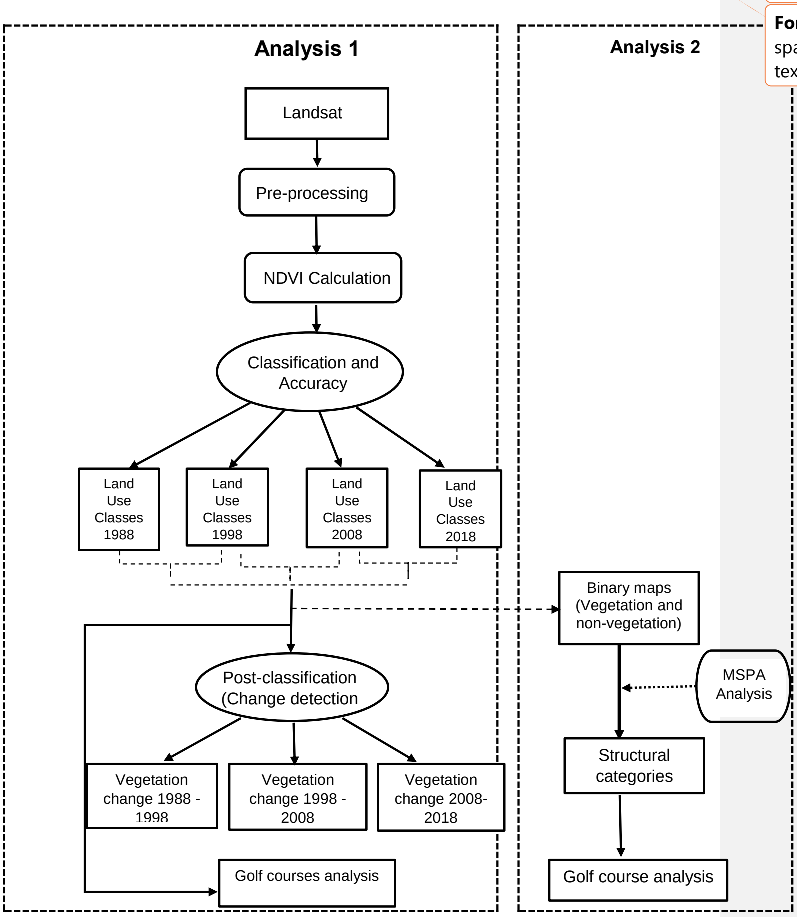

chart~~Pipelines~~ ~~Flow chart~~ summarizing the major steps taken during the investigation.

Formatted: Left

Formatted: Font: 11 pt, Bold

Formatted: Left, Space Before: 0 pt, After: 0 pt, Line spacing: single, Adjust space between Latin and Asian text, Adjust space between Asian text and numbers

Formatted: Font: (Default) Times, Bold, Font color: Auto, English (Australia), Pattern: Clear

Formatted: Font: Bold

Formatted: Font: +Body (Calibri), Bold

~~(NDVI: Normalized Difference Vegetation Index; MSPA: Morphological Spatial Pattern Analysis)~~

2.2.2 Classification

In a preliminary step, we used a decor relation stretch to enhance the image for more effective visualization. Prior to image classification, NDVI images were generated. A classification technique was then applied to the NDVI images of 1988, 1998, 2008 and 2018 using Arc-GIS 10.3 software. NDVI images were obtained by calculating the ratio between the red (R) and near infrared (NIR) values~~the visible (VIS) and near-infrared (NIR) bands~~ of the satellite image ~~by~~ using E~~e~~quation 1:

$$\mathrm{NDVI} = \frac{(\mathrm{NIR} - \cancel{\mathrm{VIS}}\mathrm{R})}{(\mathrm{NIR} + \cancel{\mathrm{VIS}}\mathrm{R})} \qquad (1)$$

In Landsat 4-7, NDVI = (Band 4 – Band 3) / (Band 4 + Band 3).

In Landsat 8, NDVI = (Band 5 – Band 4) / (Band 5 + Band 4).

Landsat TM data ~~of~~ from different dates were independently classified based on the NDVI values. ~~The w~~Water bodies have negative NDVI values, whereas, bare soil and built-up areas have an NDVI value of around zero. Chlorophyll in ~~g~~Green vegetation, on the other hand, has consumed RED to drive photosynthesis ~~stronger near-infrared reflectance~~ thereby providing moderate and high NDVI values close to +1 [52]. Based on this ~~information~~understanding, the four NDVI images were classified into three classes (V~~v~~egetation, Built up ~~and~~, bare soil, W~~w~~ater bodies) ~~by~~ using the NDVI threshold ranges technique in Arc-GIS 10.3 software.

The classification based on NDVI threshold was evaluated using accuracy assessment. An error matrix compared information from a classified image or land cover map to known reference (truth) sites for a number of sample points assessed in 2018. We obtained photographs of representative land use categories with GPS locations to assist ~~us~~ in our image interpretations. Also, we used Google Earth images, true and false colour combination images and knowledge-based information including expert knowledge, land use maps and reports. For historical images, Google Earth was used to substitute the traditional reference data collection on each of the sites [53]. Based on data of accuracy assessment, we reclassified the preliminary land use classification maps to improve the accuracy of classification.

Formatted: Font: Times, 12 pt

Formatted: Heading 3, Left, Space Before: 0 pt, After: 0 pt, Line spacing: single, Adjust space between Latin and Asian text, Adjust space between Asian text and numbers

Formatted: Font: 12 pt, Not Bold

Formatted: Font: (Default) Times, 11 pt, Font color: Auto

Formatted: Font: (Default) Times, 11 pt

Formatted: Font: 11 pt

Formatted: Font: 11 pt

Formatted: Font: 11 pt

Formatted: Font: 11 pt

Formatted: Font: Times, 11 pt

Formatted: Font: +Body (Calibri), 11 pt

Formatted: Font: (Default) Times, 11 pt, Font color: Auto

Formatted: Space After: 0 pt, Pattern: Clear

Formatted: Left, Space Before: 0 pt, After: 0 pt, Line spacing: single, Adjust space between Latin and Asian text, Adjust space between Asian text and numbers

Formatted: Font: (Default) Times, 11 pt

Formatted: Font: Times

Formatted: Font: Times

Formatted: Font: Times

Formatted: Font: (Default) Times, 11 pt, Font color: Auto, English (Australia)

Formatted: Font: (Default) Times, 11 pt

Formatted: Font: Times

Formatted: Font: (Default) Times, 11 pt

Formatted: Font: (Default) Times, 11 pt, Font color: Auto, English (Australia)

Formatted: Font: Times

Formatted: Font: Times

Formatted: Font: Times

Formatted: Font: Times

Formatted: Font: Times

Formatted: Font: Times, Font color: Auto

Formatted: Font: Times

2.2.3 Vegetation change detection

In order to detect the vegetation cover change, we created binary maps of vegetation and non-vegetation from the classified maps in the previous analysis, one for each adjacent pair of time steps, which depict where degradation occurred within a decade of urbanization. This post-classification analysis uses two images from different dates and classifies them independently. We then calculated changes in vegetation cover type using E~~e~~quation 2:

$$\text{Change area} = D_2 - D_1 \qquad (2)$$

where D1 and D2 are the area of the target vegetation cover at the beginning and the end of the study period, respectively. This analysis allows the calculation of vegetation loss and gain in each period.

2.2.4 Golf courses Analysis 1

We compared the vegetation cover, and the change ~~detection~~ (vegetation loss and gain) taking place within all golf courses in the study area, and in their surrounding regions. After creating the GIS boundaries of the~~se~~ golf courses, we extracted the vegetation cover and the vegetation change within these boundaries at four time steps in 1988, 1998, 2008 and 2018 to compare vegetation dynamics within the golf courses and the whole study area over time.

2.2.5 Morphological Spatial Pattern Analysis (MSPA) ~~MSPA analysis~~ for the structural connectivity of habitats

MSPA was employed to describe the structural connectivity of habitats in Perth for four time steps in 1988, 1998, 2008 and 2018. This method describes the spatial and temporal configuration of the ecosystem at the pixel level [32], which was based on the concept of "habitat availability" and "graphic theory" [54, 55] in which the landscape is considered as a collection of nodes, and links with a node is a place where connectivity exists and will depend on the width of itself. The output of the MSPA analysis includes the seven structural categories into which habitats are divided, including core, edge, perforation, bridge, loop, branch and islet [56] and [57] ~~which~~and ~~are~~is summarized in ~~(~~Table ~~1)~~12. [56] ~~and~~ [57]~~.~~

Formatted: Font: 12 pt, Not Bold
Formatted: Left, Space Before: 0 pt, After: 0 pt, Line spacing: single
Formatted: Font: 11 pt
Formatted: Left, Space Before: 0 pt, After: 0 pt, Line spacing: single
Formatted: Not Superscript/ Subscript
Formatted: Not Superscript/ Subscript
Formatted: Font: 12 pt, Not Bold
Formatted: Heading 3, Left, Space Before: 0 pt, After: 0 pt, Line spacing: single, Adjust space between Latin and Asian text, Adjust space between Asian text and numbers
Formatted: Font: 12 pt, Not Bold
Formatted: Font color: Auto
Formatted: Font: 11 pt, Not Bold
Formatted: Font: 11 pt, Not Bold
Formatted: Font: Bold
Formatted: Left, Space Before: 0 pt, After: 0 pt, Line spacing: single, Adjust space between Latin and Asian text, Adjust space between Asian text and numbers

Table 121.: Definition of Morphological Spatial Pattern Analysis (MSPA) MSPA classes (see [56] and [57]).

Class	Description	Ecological Meaning
Core	A collection of foreground pixels which are interconnected Foreground surrounded by foreground and greater than the user-specified edge width from background	Large-scale natural patches with high connectivity
Edge	Transition pixels between the foreground and background that form the outer edgeForeground that separates core from background	The transition zone between vegetation and non-vegetation areas. The transition zone between green space and non-green space.
Perforation	The transition pixels between foreground and background inside core areas that form the inner edge Foreground that separates core from interior areas of background	Unnatural patch inside the core area.
Bridge	A set of linear foreground pixels between two cores that connect two or more core areas.Linearly oriented	The striped ecological land that connects two cores, which is equivalent to the connecting corridor of the green space network.Striped ecological land between core areas with high connectivity, which

Formatted: Font: 11 pt
Formatted: Font: 11 pt
Formatted: Font: 11 pt
Formatted: Font: Bold
Formatted: Font: 11 pt
Formatted: Font: 11 pt
Formatted: Font: +Body (Calibri), 11 pt
Formatted: Font: 11 pt, Not Bold
Formatted ...
Formatted Table
Formatted: Font: +Body (Calibri), 11 pt, Not Bold
Formatted: Font: 11 pt, Not Bold
Formatted: Font: +Body (Calibri), 11 pt, Not Bold
Formatted: Font: 11 pt, Not Bold
Formatted: Font: 11 pt
Formatted: Font: 11 pt
Formatted ...
Formatted ...
Formatted: Font: 11 pt
Formatted: Font: (Default) +Body (Calibri), 11 pt
Formatted: Font: +Body (Calibri), 11 pt
Formatted: Font: 11 pt, Not Bold
Formatted ...
Formatted: Font: 11 pt
Formatted: Font: 11 pt
Formatted ...
Formatted: Font: +Body (Calibri), 11 pt
Formatted: Font: (Default) +Body (Calibri), 11 pt
Formatted: Font: 11 pt, Not Bold
Formatted ...
Formatted: Font: 11 pt
Formatted: Font: 11 pt
Formatted: Font: +Body (Calibri), 11 pt
Formatted: Font: +Body (Calibri), 11 pt
Formatted: Font: 11 pt, Not Bold
Formatted ...
Formatted: Font: 11 pt
Formatted: Font: 11 pt
Formatted ...

	~~foreground that connects two disjunct core areas~~	~~is equivalent to the connecting corridor of the green space network.~~
Loop	Linearly oriented foreground pixels ~~that~~ extended~~s~~ from core ~~and connects back to the same core area (e.g. a handle)~~that connects core area to itself.	Connecting corridor inside a large natural patch.
Branch	Linearly oriented foreground pixels extended from core that do not connect to any other core area	Striped ecological land with low connectivity.
Islet~~Branch~~	A collection of foreground pixels which is smaller than the core zone and do not connect to any other foreground cells~~Linearly oriented foreground that extends from core and terminates in background.~~	Small natural patches that are isolated and do not connect to each other.~~Striped ecological land with low connectivity.~~

In order to undertake the MSPA analysis, we defined the input data (foreground class). For this study, we used the classified maps for 1988, 1998, 2008, 2018 in A~~a~~nalysis 1 to create the binary maps which contained vegetation and non-vegetation classes. Hence, the high and full covered vegetation pixels were defined as the foreground pixels (green landscape) in the MSPA approach. The results of MSPA analysis for the four time steps allowed us to assess the changes in habitat connectivity ~~in the major urban area of Perth~~ over 30 years of urbanization.

2.2.6 Golf course analysis 2

To assess the role of golf courses in maintaining biodiversity connectivity over 30 years, we compared the habitat connectivity ~~taking place~~ within all golf courses in the study area and in their surrounding

Formatted: Font: +Body (Calibri), 11 pt

Formatted: Font: (Default) +Body (Calibri), 11 pt

Formatted: Font: 11 pt, Not Bold

Formatted: Left, Line spacing: 1.5 lines, Adjust space between Latin and Asian text, Adjust space between Asian text and numbers

Formatted: Font: 11 pt

Formatted: Font: 11 pt

Formatted: Line spacing: 1.5 lines, Adjust space between Latin and Asian text, Adjust space between Asian text and numbers

Formatted: Font: (Default) +Body (Calibri), 11 pt

Formatted: Font: +Body (Calibri), 11 pt

Formatted: Font: 11 pt, Not Bold

Formatted: Left, Line spacing: 1.5 lines, Adjust space between Latin and Asian text, Adjust space between Asian text and numbers

Formatted: Font: 11 pt

Formatted: Font: 11 pt

Formatted: Line spacing: 1.5 lines, Adjust space between Latin and Asian text, Adjust space between Asian text and numbers

Formatted: Font: (Default) +Body (Calibri), 11 pt

Formatted: Font: +Body (Calibri), 11 pt

Formatted: Font: 11 pt, Not Bold

Formatted: Left, Line spacing: 1.5 lines, Adjust space between Latin and Asian text, Adjust space between Asian text and numbers

Formatted: Font: 11 pt

Formatted: Font: 11 pt

Formatted: Line spacing: 1.5 lines, Adjust space between Latin and Asian text, Adjust space between Asian text and numbers

Formatted: Font: (Default) +Body (Calibri), 11 pt

Formatted: Font: +Body (Calibri), 11 pt

Formatted: Left, Space Before: 0 pt, After: 0 pt, Line spacing: single

Formatted: Font: Not Bold

Formatted: Font: 12 pt, Not Bold

Formatted: Heading 3, Left, Space Before: 0 pt, After: 0 pt, Line spacing: single, Adjust space between Latin and Asian text, Adjust space between Asian text and numbers

Formatted: Pattern: Clear

Formatted ...

green spaces~~regions~~. Using the GIS boundaries of the golf courses, we extracted the habitat connectivity within the golf course boundaries for four time steps (1988, 1998, 2008 and 2018).

3 Results

3.1 Land use classification in Perth

Data sets representing four time periods (1988, 1998, 2008 and 2018) are shown in Figure 3 and Table ~~2~~3, which provide~~s~~ an overview of the land ~~use~~ cover changes (vegetation, built up and bare land, water bodies) over recent decades. From the 1988 and 1998 data sets, it is evident that over half of the region was vegetated. However, the urban footprint of built up and bare land area had increased 10% from 1998 to ~~by~~ 2018. As a consequence, there was a significant decrease in vegetation cover, which comprised 56% of the land surface in 1988 and declined by 10.1% over the next 30 years (Table ~~2~~2~~3~~).

~~We obtained anThe analysis had an overall accuracy (OA) of classification of 87% and a kappa coefficient of 91% for the three classes.~~

Table ~~2~~3~~2~~. Land cover~~use~~ classification within the Perth Metropolitan Region.

~~Land use category~~		Land cover~~use~~ category					
		Whole area			Golf course		
		Vegetation	Built up and bare soil	Water bodies	Vegetation	Built up and bare soil	Water bodies
1988	Area (ha)	98,446	74,376	2,667	929	210	0.1

Formatted: Font: +Body (Calibri), Pattern: Clear
Formatted: Font: (Default) Times, 12 pt, Font color: Text 1
Formatted: Font: (Default) Times, 12 pt
Formatted: Heading 2, Space Before: 0 pt, After: 0 pt, Line spacing: single
Formatted: Font: (Default) Times, 12 pt, Not Bold
Formatted: Left, Space Before: 0 pt, After: 0 pt, Line spacing: single, Adjust space between Latin and Asian text, Adjust space between Asian text and numbers
Formatted
Formatted: Font: Not Bold
Formatted: Font: Not Bold
Formatted: Font: Bold
Formatted: Left, Space Before: 0 pt, After: 0 pt, Line spacing: single, Adjust space between Latin and Asian text, Adjust space between Asian text and numbers
Formatted
Formatted: Line spacing: Multiple 1.15 li
Formatted Table
Formatted: Font: 11 pt
Formatted: Left, Line spacing: Multiple 1.15 li
Formatted: Line spacing: Multiple 1.15 li
Formatted: Font: (Default) +Body (Calibri), 11 pt
Formatted
Formatted: Left, Line spacing: Multiple 1.15 li
Formatted: Font: (Default) +Body (Calibri), 11 pt
Formatted
Formatted
Formatted
Formatted
Formatted
Formatted: Font: 11 pt
Formatted: Left, Line spacing: Multiple 1.15 li
Formatted: Font: (Default) +Body (Calibri), 11 pt
Formatted
Formatted
Formatted
Formatted
Formatted
Formatted

~~Land use category~~		Land cover~~use~~ category					
		Whole area			Golf course		
		Vegetation	Built up and bare soil	Water bodies	Vegetation	Built up and bare soil	Water bodies
	Proportion (%)	56.1	42.4	1.5	81.5	18.5	0.0
1998	Area (ha)	91,754	81,464	2,271	1,042	97	0.7
	Proportion (%)	52.3	46.4	1.3	91.4	8.5	0.1
2008	Area (ha)	88,341	84,971	2,176	1,084	55	0.2
	Proportion (%)	50.3	48.4	1.2	95.1	4.9	0.0
2018	Area (ha)	80,755	92,243	2,491	1,093	46	0.7
	Proportion (%)	46.0	52.6	1.4	95.9	4	0.1

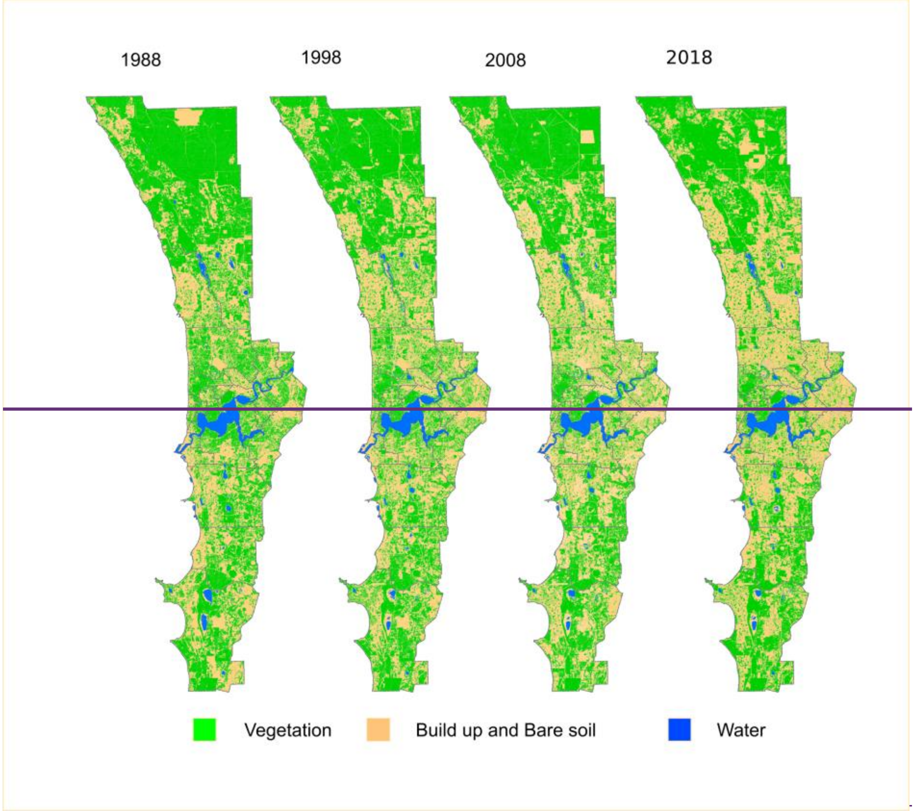

Figure 3. Map of land cover~~use~~ classification for Perth in four time steps between 1988 and 2018~~.~~

The analysis had the overall accuracy (OA) of classification from 87% and the kappa coefficient from 91% for the three classes (Table 3).

Table 3. Accuracy assessment of land cover maps generated

		Year 1988		
LULC Class	Vegetation	Build Up and Bare Soil	Water Bodies	Total
Vegetation	14	1	0	15
Build Up and Bare Soil	1	12	2	15
Water Bodies	0	0	15	15
Total	15	13	17	45
		Overal Accuracy	87%	
		Overal Kappa	0.91	
		**Year 1998**		
LULC Class	Vegetation	Build Up and Bare Soil	Water Bodies	Total
Vegetation	15	0	0	15
Build Up and Bare Soil	0	13	2	15
Water Bodies	1	0	14	15
Total	16	13	16	45
		Overal Accuracy	90%	
		Overal Kappa	0.93	
		**Year 2008**		
LULC Class	Vegetation	Build Up and Bare Soil	Water Bodies	Total
Vegetation	14	1	0	15
Build Up and Bare Soil	1	14	0	15
Water Bodies	0	0	15	15
Total	15	15	15	45
		Overal Accuracy	93%	
		Overal Kappa	0.96	
		**Year 2018**		
LULC Class	Vegetation	Build Up and Bare Soil	Water Bodies	Total
Vegetation	15	0	0	15
Build Up and Bare Soil	0	13	2	15
Water Bodies	1	1	13	15
Total	16	14	15	45
		Overal Accuracy	87%	
		Overal Kappa	0.91	

Using the GIS layer of land use categories, we also extracted the data for three classes within the golf courses in comparison with the whole area (Table 223). Our analysis shows that while there was a significant decrease in vegetation cover throughout the region, the total area of golf courses remained unchanged at around 1,019340 ha over the last 30 years.

Figure 4. Land cover classification showing a detailed view of the Collier Park Golf Course for a) Landsat data; b) NDVI values; c) Land cover classificationFigure 4. Land cover changes in the Collier Park Golf Course throughout the time

3.2 Spatial patterns of vegetation change

To characterize spatial patterns of vegetation dynamics, we detected the vegetation loss and gain for each of the three decades (FigFigure 54). In the period 1988 to 1998, vegetation clearingdeforestation occurred intensively in the central and north regions. From 1998 to 2008, vegetation loss expanded to the north and south of the city. However, in the last decade from 2008 to 2018, vegetation loss accelerated in the distal regions with urbanization. However, there was also some vegetation gain over the three decades (FigFigure 54, Table 344) predominantly in the northern part of the city.

Formatted: English (Australia)

Formatted: Font: (Default) Times New Roman, 12 pt, Bold

Formatted: Font: Bold

Formatted: Normal, Left, Space Before: 0 pt, After: 0 pt, Line spacing: single

Formatted: Font: (Default) Times New Roman, 12 pt, Bold

Formatted: Font: (Default) Times New Roman, 12 pt, Bold

Formatted: Normal, Left, Space Before: 0 pt, After: 0 pt, Line spacing: single

Formatted: Font: (Default) Times, 12 pt

Formatted: Heading 2, Space Before: 0 pt, After: 0 pt, Line spacing: single

Formatted: Font: (Default) Times, 12 pt, Not Bold

Formatted: English (Australia)

Formatted: Normal, Left, Space Before: 0 pt, After: 0 pt, Line spacing: single

Formatted: English (Australia)

Formatted: English (Australia)

Figure ~~5~~5~~4~~4. Change in vegetation cover over the period 1988 to 2018 for the Perth region ~~three decades.~~

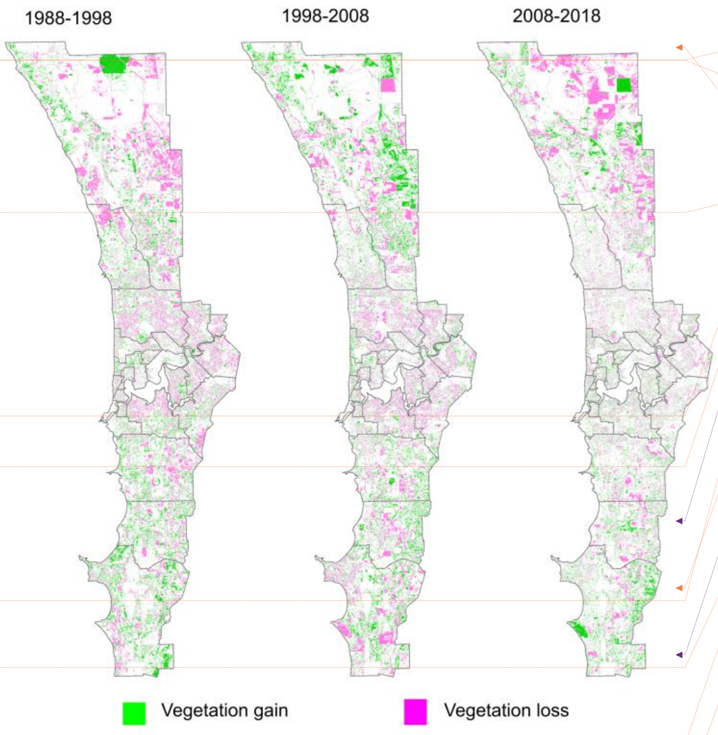

Table ~~4~~4~~3~~. Vegetation loss and gain (ha) between 1988 and 2018~~in three time periods over the period 1988 to 2018~~.

		Period		
~~Period~~		1988-1998	1998-2008	2008-2018
Whole Area	Vegetation loss	22,231	19,250	20,178
	Vegetation gain	15,538	15,838	12,591
	Net loss	6,692	3,412	7,587
Golf courses	Vegetation loss	68	27	40
	Vegetation gain	219	92	43
	Net gain	152	64	4

Calculation of changes within the golf courses and in the whole area (Table ~~3~~44) showed that the major urban area of Perth experienced a net loss in vegetation cover. Though vegetation compensation

Formatted: Normal, Left, Space Before: 0 pt, After: 0 pt, Line spacing: single
Formatted: Font: Bold, English (Australia)
Formatted: Font: Bold, English (Australia)
Formatted: Normal, Left, Space Before: 0 pt, After: 0 pt, Line spacing: single
Formatted: Font: Bold, English (Australia)
Formatted: Font: Bold, English (Australia)
Formatted: Font: Bold
Formatted: Normal, Left, Space Before: 0 pt, After: 0 pt, Line spacing: single
Formatted: Font: Bold
Formatted: Normal, Left, Space Before: 0 pt, After: 0 pt, Line spacing: single
Formatted: Normal, Left, Space Before: 0 pt, After: 0 pt, Line spacing: single
Formatted: Font: Bold
Formatted: Font: (Default) Times, 11 pt, Bold, Font color: Auto, English (Australia)
Formatted: Font: Bold
Formatted: Font: Bold, English (Australia)
Formatted Table
Formatted: Centered
Formatted: Font: (Default) Times, 11 pt, Font color: Auto
Formatted: Left, Line spacing: single
Formatted: Font: (Default) Times, 11 pt, Font color: Auto
Formatted: Left, Space Before: 0 pt, After: 0 pt, Line spacing: single
Formatted: Left, Line spacing: single
Formatted: Left, Line spacing: single
Formatted: Left, Line spacing: single
Formatted: Font: (Default) Times, 11 pt, Font color: Auto
Formatted: Left, Space Before: 0 pt, After: 0 pt, Line spacing: single
Formatted: Left, Line spacing: single
Formatted: Left, Line spacing: single
Formatted: Left, Line spacing: single
Formatted: Normal, Left, Space Before: 0 pt, After: 0 pt, Line spacing: single

occurred together with ~~vegetation clearing~~deforestation over 30 years of urbanization, the vegetation loss was always much larger than vegetation gain with the largest net vegetation loss occurring in the last decade. However, the golf courses showed a different trend where the net gain of vegetation cover happened over three decades and the largest gain was between 1988 and 1998.

3.3 *Analysis of connectivity components of green space networks*

Results of the MSPA analysis indicate that the reduction in vegetation cover over the last thirty years has led to a decline in connectivity (Table 5~~5~~). Among the two ~~types of landscape~~MSPA classes that are important for connectivity (core and bridge), the total area of core class decreased by about 10% over three decades while the bridge class was maintained at around 37,000 ha, but the proportion of this class per total vegetation cover area (VCA) increased due to the reduction of vegetation cover over time (Table 4~~5~~5).This analysis also shows the fluctuation in the areas of the rest of the MSPA classes including islets, loops, edges, perforations, and branches which do not contribute to connectivity in the landscape. The proportion of these classes increased through time from 24% in 1988 to 30% in 2008 and 2018.

Figure 6~~5~~ shows that the core area was distributed mostly in the northern part of the city and their areas decreased significantly in later years. In 1988, the bridge class covered a large area of the city's central region but this decreased over time. In recent years, most of the vegetation cover in the central region of the city belongs to the islet, loop, edge, perforation and branch classes, illustrating that isolation became more serious in the central region of the city over the three decades.

Figure 6~~5~~. Results of the Morphological Spatial Pattern Analysis (MSPA) for the Perth region~~MSPA~~ from 1988 to 2018~~analysis~~

The vegetation cover within golf courses also contributes to connectivity. The proportion of core area within golf courses fluctuated between 12% and 27%. Moreover, the largest proportion of vegetation in golf courses was classified as bridge but it experienced a downward trend from 52% to 41% in three decades. Of the remaining classes which do not contribute to connectivity, the edge and loop classes accounted for a higher proportion with each of them contributing 7% to 16% of total vegetation cover.

Formatted: Font: (Default) Times, 12 pt

Formatted: Heading 2, Space Before: 0 pt, After: 0 pt, Line spacing: single

Formatted: Font: (Default) Times, 12 pt, Not Bold

Formatted: Normal, Left, Space Before: 0 pt, After: 0 pt, Line spacing: single

Formatted: Font: (Default) Times

Formatted: Left, Space Before: 0 pt, After: 0 pt, Line spacing: single, Adjust space between Latin and Asian text, Adjust space between Asian text and numbers

Formatted: Font: (Default) Times

Formatted: Font: (Default) Times

Formatted: Font: (Default) Times

Formatted: Font: Bold

Formatted: Font: Bold

Formatted: Font: Bold

Formatted: Font: Not Bold, Font color: Auto

Formatted: Normal, Space Before: 0 pt, After: 0 pt, Line spacing: single

Formatted: Font: Not Bold, Font color: Auto

Formatted: Normal, Space Before: 0 pt, After: 0 pt, Line spacing: Multiple 1.08 li

Table 554. Results of Morphological Spatial Pattern Analysis (MSPA)MSPA analysis of connectivity of Perth's vegetation from 1988 to 2018.

Landscape type	Year	Perth Metropolitan Region		Within golf courses	
		Area (ha)	Proportion of total VCA (%)	Area (ha)	Proportion of total VCA (%)
Core	1988	32,012	32.6	325	23.2
	1998	20,607	22.5	171	12.7
	2008	22,251	25.3	230	16.7
	2018	18,101	22.5	383	27.3
Bridge	1988	37,127	37.9	738	52.6
	1998	39,412	43.0	814	60.2
	2008	37,902	43.1	732	53.1
	2018	37,503	46.7	588	42.0
Islet	1988	10,396	10.6	31	2.2
	1998	16,436	17.9	78	5.8
	2008	12,922	14.7	15	1.1
	2018	12,349	15.4	37	2.7
Perforation	1988	1,099	1.1	3	0.2

Landscape type	Year	Perth Metropolitan Region		Within golf courses	
		Area (ha)	Proportion of total VCA (%)	Area (ha)	Proportion of total VCA (%)
	1998	486	0.5	-	-
	2008	834	1.0	-	-
	2018	406	0.5	6	0.4
Edge	1988	7,430	7.6	162	11.5
	1998	4,814	5.3	95	7.0
	2008	4,986	5.7	147	10.7
	2018	5,218	6.5	230	16.4
Loop	1988	5,670	5.8	112	8.0
	1998	6,295	6.9	146	10.8
	2008	5,607	6.4	209	15.2
	2018	3,669	4.6	129	9.2
Branch	1988	4,361	4.5	32	2.3
	1998	3,619	4.0	47	3.5
	2008	3,491	4.0	43	3.2
	2018	3,117	3.9	29	2.1

4 Discussion

We used Landsat imagery to characterize the patterns of vegetation change, habitat connectivity and the role of golf courses in maintaining green spaces and connectivity in an urban landscape. We found that ~~vegetation clearing~~deforestation led to a reduction in habitat connectivity in the Perth Metropolitan Region. However, golf courses can play an important role in maintaining vegetation and supporting biodiversity connectivity in urban landscapes. ~~Prior work~~~~Privious~~Previous study~~-~~ies ~~has~~ have documented the increase in the urban footprint of Perth using multi-temporal urban expansion statistics derived from Satellite imagery [58]; ~~-~~However, ~~this study~~their work did not address the issues of vegetation dynamics, nor the biodiversity and structure of the vegetation and habitat

Formatted: Font: (Default) Times
Formatted: Left, Line spacing: single
Formatted Table
Formatted: Font: (Default) Times, 11 pt
Formatted: Left, Line spacing: single
Formatted: Font: (Default) Times, 11 pt
Formatted: Left, Line spacing: single
Formatted: Font: (Default) Times, 11 pt
Formatted: Left, Line spacing: single
Formatted: Font: (Default) Times
Formatted: Line spacing: single
Formatted: Font: (Default) Times, 11 pt
Formatted: Left, Line spacing: single
Formatted: Font: (Default) Times, 11 pt
Formatted: Left, Line spacing: single
Formatted: Font: (Default) Times, 11 pt
Formatted: Left, Line spacing: single
Formatted: Font: (Default) Times, 11 pt
Formatted: Left, Line spacing: single
Formatted: Font: (Default) Times
Formatted: Line spacing: single
Formatted: Font: (Default) Times, 11 pt
Formatted: Left, Line spacing: single
Formatted: Font: (Default) Times, 11 pt
Formatted: Left, Line spacing: single
Formatted: Font: (Default) Times, 11 pt
Formatted: Left, Line spacing: single
Formatted: Font: (Default) Times
Formatted: Font: (Default) Times, 11 pt
Formatted: Line spacing: single
Formatted: Left, Line spacing: single
Formatted: Left, Line spacing: single
Formatted: Left, Line spacing: single
Formatted: Left, Line spacing: single
Formatted: Line spacing: single
Formatted ...
Formatted ...
Formatted ...

connectivity in the Perth Metropolitan Region. Therefore, our study ~~have worked to fill~~addresses this gap.

4.1 ~~Vegetation clearing~~Deforestation and urbanization

The vegetation dynamics in the major urban areas of Perth reflect the pattern of urbanization over time. The reduction in green space found in this study can be explained as a close relation to the process of development in this city. Over the last three decades, urban development in Perth has taken place at an unsustainable rate [42]. In the early 1990s to 2006, Perth's population grew by around 1.8%, but the figure has nearly doubled since then [59]. Also, this study indicated that, from 1988 to 2018, Perth's urban footprint increased from 74,376 ~~thousand~~ to 92 ~~thousand~~,243 ha (Table ~~2~~2~~3~~) and is consistent with previous research in urban growth in this region [58]. ~~In addition to expansion, the city has become denser due to the construction of new residential dwellings in urbanised areas.~~ In the last 20 years, on average, 740 ha/yr of urban and urban deferred zoned land was consumed by subdivision, and 830 ha/yr was consumed by construction in the Perth metropolitan area and nearby Peel region [60]. In addition to this expansion, the city has become denser with the construction of new residential dwellings in urbanised areas. Though vegetation gain occurred in some places as a result of natural increase in canopy cover as urban vegetation grows through the conservation efforts, vegetation offset from development projects (e.g. mining), the plantation programs of the government taking place in bare soils in some suburbs, and efforts to increase green spaces from private land owners, the vegetation loss associated with urbanization has been more significant.

The difference in spatial patterns of vegetation loss are also related to the urban plans of this city. Our results show that between 1988 and 1998, there was significant vegetation loss in the central region of the city which can be linked to The Corridor Plan [61]. Historically, Perth's development pattern from 1970s to 1990s was based on linear corridors stretching out from the city's core, with large non-urban wedges between each of these corridors [61]. However, from 1998 to 2008 and from 2008 to 2018, ~~vegetation clearing~~deforestation was more significant in the outer subregions ~~of the~~ north-west and south-west of the city due to the adoption of ~~METROPLAN~~ Metroplan [62]. Perth recently has been divided into subregional areas, rather than corridors for planning purposes. The two coastal subregions

Formatted: Font: Not Italic

Formatted: Font: (Default) Times, 12 pt

Formatted: Font: (Default) Times, 12 pt, Not Bold

Formatted: Font: Not Italic

Formatted: Font: Not Italic

(the North-West and South-West) included in our study have consistently achieved higher rates of population growth under Metroplan [63].

Originally the region was covered by woodlands dominated by eucalypts and banksias and coastal heath interspersed with chains of wetlands. Perth is home to a rich biodiversity, with more than 1,700 species of flowering plants and iconic species of threatened fauna in the region [64]. Therefore, such ~~vegetation clearing~~deforestation for urbanization has resulted in the devastating loss of significant natural habitats in this biodiversity hotspot city, leading to the designation ~~of~~ as an endangered ecological community by the Australian Government [41].

4.2 Connectivity of green space networks in urban landscapes

The MSPA analysis indicates that the loss ~~of area~~ of vegetation as a consequence of urban expansion has led to a marked reduction in connectivity of green space networks in the Perth urban landscape. ~~In the MSPA analysis,~~ As only cores (the stepping stones between forest habitat patches) and bridges (the structural corridors to link core areas) can contribute to the connectivity between the habitat areas in the landscape [65]. t~~T~~he reduction of these ~~areas~~ areas in Perth ~~our stud~~associated with urbanization throughout the time ~~y~~ indicates the high impact of ~~urbanization~~ urban development on habitat connectivity~~, through the loss of core areas, which act as stepping stones between forest habitat patches, and the loss of bridges that act as structural corridors to link two or more core areas~~ [65]. This analysis also shows the increase in proportion of islets, which are totally isolated patches, and other classes (perforations, loops, branches, edges) that cannot reach a new core habitat area for originating the potential movement [65]. Clearly, expansion of Perth city has fragmented the remaining blocks of natural habitat and increased isolation of natural habitats. This may reduce population and gene flow among patches and may disrupt the connection between subpopulations and a large regional population [66] and thus threaten the long-term viability of relict populations.

~~The f~~Fragmentation ~~process~~ was obvious in the central region of the city and in recent decades it has become more serious in the outer parts of the city. This is critical as Perth is within a globally recognised biodiversity hotspot, which is home to rich biodiversity found nowhere else in the world. The connectivity in the major urban area of Perth is not only critical for the linkage of habitats within

Formatted: Font: (Default) Times, 12 pt

Formatted: Font: (Default) Times, 12 pt, Not Bold

Formatted: Not Expanded by / Condensed by

Formatted: Not Expanded by / Condensed by

Formatted: Font color: Auto, Not Expanded by / Condensed by

the Swan Coastal Plain but also for the connection of these coastal habitats to a large regional biosphere in south-western Australia.

The results also illustrated that a ‘Core’ area and a network of ‘Bridge’ types exist in the central and outer subregions of Perth. This is the consequence of early conservation efforts of the government which created protected areas such as Kings Park, Bold Park and other significant areas. Very few cities in the world have such large areas of natural bushland in the centre of a big city [67]. However, future urban growth will continue to put pressure on the biodiversity. If current policies (Perth and Peel@3.5million) are fully implemented, existing stocks of urban and urban deferred land would be consumed by about 2075 [68]. The challenge for urban planning to preserve urban forests and biodiversity is thus increasing, and it is clear that planning for future expansion should also include large protected areas. .

Our MSPA output with spatial distribution of seven classes provides fundamental information for future urban planning. There is a need to maintain important green spaces which are classified as cores and bridges in the city, especially in the central region where most of the natural vegetation exists as islands. Also, the MSPA branch classes can be used to identify candidate ecological restoration areas. The branch class can be thought of [69] as a foundation of a potential corridor that could, if revegetated, connect two spatially disjunct core areas to improve connectivity in the larger region.

Although other analyses, such as functional connectivity, should be taken into account in landscape connectivity assessment [32], the structural connectivity analysis in this study will be useful for determining the priority protection level and critical areas of the connecting corridor, informing conservation strategies at a variety of scales, especially when the biodiversity values of this region are suffering from various threats including ~~land clearing~~deforestation, feral animals, weed incursions, more frequent fires through arson and tree disease [64].

4.3 The role of golf courses in maintaining urban forest

Our study indicates that golf courses account for a significant proportion of the urban area of Perth. This category of land use has been vital in maintaining green space in urban areas over the past thirty

Formatted: Left, Space Before: 0 pt, After: 0 pt, Line spacing: single

Formatted: Font: (Default) Times, 12 pt

Formatted: Font: (Default) Times, 12 pt, Not Bold

Formatted: Heading 2, Left, Space Before: 0 pt, After: 0 pt, Line spacing: single, Adjust space between Latin and Asian text, Adjust space between Asian text and numbers

years. In contrast to the overall decline in urban green space, golf courses have preserved green spaces within urban settings and even created a net gain of vegetation cover over time. The highest net gain was seen in the period between 1988 to 1998 when some golf courses were established resulting in the planting of trees.

In the green 'matrix' of Perth, golf courses with their significant area of vegetation cover have contributed considerably to the connectivity in the urban landscape. A significant proportion of their green space was classified as core or bridge categories. The proportion of vegetation within golf courses classified as bridges was higher than in the whole study area. Golf courses with large areas of native vegetation provide "links" to other large natural patches of urban vegetation.

Although there are concerns with the environmentally negative impacts of golf courses as a source of pollution through pesticide and fertiliser usage [22], habitat modification [25] and high water usage [70], previous studies provide evidence about the biological values of golf courses, such as providing refugial habitat for urban-avoiding wildlife [24, 71-79]. Our study indicates that golf courses in urban settings have been maintaining large green-area habitats and played an important role in biodiversity connectivity in the city.

Formatted: Font color: Auto

4.4 *Monitoring urban forest dynamics*

Formatted: Font: (Default) Times, 12 pt

Formatted: Font: (Default) Times, 12 pt, Not Bold

In this study, we utilized medium-resolution satellite remote sensing data to identify land use classes, characterise vegetation dynamics and connectivity. The data maps the spatial and temporal patterns of land use types characterizing a consistent, detailed vegetation dynamic of the city [58, 80]. Clearly, the biophysical elements of urban landscapes are well-reflected through physical features (NDVI) derived from remote sensing data with an accuracy of up to 89%.

Despite these kinds of data, it is hard to describe the detailed information of ecosystem such as species composition and forest structure. However, they show their advantage in mapping land cover dynamics across large areas of big cities over time when high resolution imagery is not available. For monitoring urban forests in big cities, the large scale and long temporal datasets are more advantageous compared with datasets that focus only on discerning a specific land use type in a

relatively small area [27, 81, 82]. This is because it allows spatially detailed identification of changes associated with development over time. Therefore, ~~this~~ the approach described in this paper ~~method~~ provides baseline information for sustainable urban planning and development. In addition, the MSPA analysis can further evaluate the dynamic of vegetation cover by the describing the spatial configuration of ecosystems at the pixel level, detecting changes of habitat connectivity over time [32].

5 Conclusions

With rapid urban expansion, the most meaningful question to address is how to balance urban development and urban forest preservation. Urbanization requiring ~~vegetation clearing~~deforestation is inevitable in many cities worldwide. Our study found a significant loss of vegetation cover in a biodiversity hotspot over three decades of urbanization, which led to a reduction in habitat connectivity in the urban landscape. A lesson learned from the experience of urbanization in Perth is that any future urban growth following the patterns observed over the past three decades will continue to put pressure on maintaining urban forest ecosystems and biodiversity conservation. As cities continue to grow in response to socio-economic development, considering all opportunities for urban biodiversity conservation is important. Urban conservation strategies must therefore consider not only the protected areas, but also the off-reserve sites.

Our study indicates that golf courses in urban settings have been maintaining green-area habitats and have played an important role in biodiversity connectivity in the city. Potentially, urban golf courses could become more purposefully managed for biodiversity conservation and the improvement of critical ecosystem services in urban areas. In ~~the~~ rapidly urbanizing biodiversity hotspots like Perth, where fragmentation is one the biggest threats to biodiversity, the way that golf courses contribute to increase the connectivity in the intervening urban matrix should not be underestimated. Therefore, it is important for government authorities and golf courses owners to pay more attention in maintaining ecosystem health in urban golf courses.

Formatted: Font: (Default) Times, 12 pt, Font color: Text 1

Formatted: Left, Space Before: 0 pt, After: 0 pt, Line spacing: single, Adjust space between Latin and Asian text, Adjust space between Asian text and numbers

Formatted: Space After: 0 pt, Pattern: Clear

6 Acknowledgments

The authors thank Dr. Harry Eslick for his valuable advice in helping to design the research and Mr. Pham Dang Manh Hong Luan for his advice in data analysis. The research was supported by the Vietnamese Ministry of Education and Training and Murdoch University through a VIED-MU joint PhD scholarship to the first author.

7 References

1. Tabea TaE, Knop. A landscape ecology approach identifies important drivers of urban biodiversity. Global Change Biol. 2015;21 (4):1652-67.
2. Bagan H, Yamagata Y. Land-cover change analysis in 50 global cities by using a combination of Landsat data and analysis of grid cells. Environmental Research Letters. 2014;9(6):064015. doi: 10.1088/1748-9326/9/6/064015.
3. UNDESA UNDoEaSA. World urbanization prospects: The 2011 revision. United Nations Department of Economic and Social Affairs/Population Division, New York: 2012.
4. McGrane SJ. Impacts of urbanisation on hydrological and water quality dynamics, and urban water management: a review. Hydrological Sciences Journal. 2016;61(13):2295-311. doi: 10.1080/02626667.2015.1128084.
5. Gaston KJ, Ávila-Jiménez ML, Edmondson JL. REVIEW: Managing urban ecosystems for goods and services. Journal of Applied Ecology. 2013;50(4):830-40. doi: 10.1111/1365-2664.12087.
6. Declet-Barreto J, Brazel AJ, Martin CA, Chow WTL, Harlan SL. Creating the park cool island in an inner-city neighborhood: heat mitigation strategy for Phoenix, AZ. Urban Ecosystems. 2013;16(3):617-35. doi: 10.1007/s11252-012-0278-8.
7. Scott AA, Misiani H, Okoth J, Jordan A, Gohlke J, Ouma G, et al. Temperature and heat in informal settlements in Nairobi. PLOS ONE. 2017;12(11):e0187300. doi: 10.1371/journal.pone.0187300.
8. Van Den Berg AE, Custers MHG. Garndening promotes neuroendocrine and affective restoration from stress. Journal of Health Psychology. 2010;16(1):1359-053.
9. Mitchell R. Is physical activity in natural environments better for mental health than physical activity in other environments? Social Science & Medicine. 2013;91(Supplement C):130-4. doi: https://doi.org/10.1016/j.socscimed.2012.04.012.
10. Donovan GH, Butry DT, Michael YL, Prestemon JP, Liebhold AM, Gatziolis D, et al. The relationship between trees and human health: Evidence from the spread of the emerald ash borer. American Journal of Preventive Medicine. 2013;44(2):139-45. doi: https://doi.org/10.1016/j.amepre.2012.09.066.
11. Ulrich RS. View through a window may influence recovery from surgery. Science. 1984;224(4647):420.
12. Cecily Jane M. Promoting children's mental, emotional and social health through contact with nature: a model. Health Education. 2009;109(6):522-43. doi: 10.1108/09654280911001185.
13. Lee KE, Williams KJH, Sargent LD, Williams NSG, Johnson KA. 40-second green roof views sustain attention: The role of micro-breaks in attention restoration. Journal of Environmental Psychology. 2015;42(Supplement C):182-9. doi: https://doi.org/10.1016/j.jenvp.2015.04.003.
14. Kim G. Assessing Urban Forest Structure, Ecosystem Services, and Economic Benefits on Vacant Land. Sustainability. 2016;8(7):679. PubMed PMID: doi:10.3390/su8070679.
15. Zitkovic M. Managing green spaces for urban biodiversity. Boulevard Louis Schmidt 64 1040 Brussels, Belgium: Countdown 2010 Secretariat, IUCN Regional Office for Europe, 2008.

Formatted: Font: (Default) Times, 12 pt, Font color: Text 1

Formatted: Font: (Default) Times, 12 pt, Not Bold, Font color: Text 1

Formatted: Left, Line spacing: single, Adjust space between Latin and Asian text, Adjust space between Asian text and numbers

Formatted: Font: (Default) Times, 12 pt

Formatted: Heading 1, Space Before: 0 pt, After: 0 pt, Line spacing: single, Adjust space between Latin and Asian text, Adjust space between Asian text and numbers

Formatted: Font: (Default) Times, 12 pt, English (Australia), Check spelling and grammar

Formatted: Font: (Default) Times, 11 pt, English (Australia), Check spelling and grammar

16. Seitz J, Escobedo F. Urban forests in Florida: Trees control stormwater runoff and improve water quality. University of Florida, Institute of Food and Agricultural Sciences. IFAS Extension Publication FOR184.2014.
17. Bailey D, Schmidt-Entling MH, Eberhart P, Herrmann JD, Hofer G, Kormann U, et al. Effects of habitat amount and isolation on biodiversity in fragmented traditional orchards. Journal of Applied Ecology. 2010;47(5):1003-13. doi: 10.1111/j.1365-2664.2010.01858.x.
18. Yu D, Liu Y, Xun B, Shao H. Measuring Landscape Connectivity in a Urban Area for Biological Conservation. CLEAN – Soil, Air, Water. 2015;43(4):605-13. doi: 10.1002/clen.201200448.
19. Bierwagen B. Connectivity in urbanizing landscapes: The importance of habitat configuration, urban area size, and dispersal2007. 29-42 p.
20. Lindenmayer D, Franklin J. Conserving forest biodiversity: a comprehensive multiscaled approach2002.
21. Napton DE, Laingen CR. Expansion of Golf Courses in the United States. Geographical Review. 2008;98(1):24-41.
22. Guzmán C, & Fernández, D.,Javier Mesa. Environmental impaccts by golf courses and strategies to minimize them: state of the art. International Journal of Arts & Sciences. 2014;7(3):403-17.
23. Colding J, Folke C. The Role of Golf Courses in Biodiversity Conservation and Ecosystem Management. Ecosystems. 2009;12(2):191-206. doi: 10.1007/s10021-008-9217-1.
24. Hodgkison SC, Hero JM, Warnken J. The conservation value of suburban golf courses in a rapidly urbanising region of Australia. Landscape and Urban Planning. 2007;79(3):323-37. doi: https://doi.org/10.1016/j.landurbplan.2006.03.009.
25. Terman MR. Natural links: naturalistic golf courses as wildlife habitat. Landscape and Urban Planning. 1997;38(3):183-97. doi: https://doi.org/10.1016/S0169-2046(97)00033-9.
26. Puglis HJ, Boone MD. Effects of Terrestrial Buffer Zones on Amphibians on Golf Courses. PLOS ONE. 2012;7(6):e39590. doi: 10.1371/journal.pone.0039590.
27. Theobald DM. Development and Applications of a Comprehensive Land Use Classification and Map for the US. PLOS ONE. 2014;9(4):e94628. doi: 10.1371/journal.pone.0094628.
28. Hu T, Yang J, Li X, Gong P. Mapping Urban Land Use by Using Landsat Images and Open Social Data. Remote Sensing. 2016;8(2):151. PubMed PMID: doi:10.3390/rs8020151.
29. Li X, Liu X, Gong P. Integrating ensemble-urban cellular automata model with an uncertainty map to improve the performance of a single model. International Journal of Geographical Information Science. 2015;29(5):762-85. doi: 10.1080/13658816.2014.997237.
30. Tucker CJ. Red and photographic infrared linear combinations for monitoring vegetation. Remote Sensing of Environment. 1979;8(2):127-50. doi: https://doi.org/10.1016/0034-4257(79)90013-0.
31. Myneni RB, Hall FG, Sellers PJ, Marshak AL. The interpretation of spectral vegetation indexes. IEEE Transactions on Geoscience and Remote Sensing. 1995;33(2):481-6. doi: 10.1109/TGRS.1995.8746029.
32. Vogt P, Riitters K, Estreguil C, Kozak J, G. Wade T, Wickham J. Mapping Spatial Patterns with Morphological Image Processing2007. 171-7 p.
33. Myers N, Mittermeier RA, Mittermeier CG, da Fonseca GAB, Kent J. Biodiversity hotspots for conservation priorities. Nature. 2000;403(6772):853-8. doi: 10.1038/35002501.
34. Kottek MG, Jürgen; Beck, Christoph; Rudolf, Bruno; Rubel, Franz. World Map of the K \ o ppen-Geiger climate classification updated. Meteorologische Zeitschrift. 2006;15(3):259-63. doi: 10.1127/0941-2948/2006/0130.
35. Hope PK, Drosdowsky W, Nicholls N. Shifts in the synoptic systems influencing southwest Western Australia. Climate Dynamics. 2006;26(7):751-64. doi: 10.1007/s00382-006-0115-y.
36. Mittermeier R, Gil P, Hoffmann M, Pilgrim J, Brooks T, Mittermeier C, et al. Hotspots Revisited. Earth's Biologically Richest and Most Endangered Terrestrial Ecoregions2004.
37. Environmental Protection Act 1986, (1986).

38. Environment Protection and Biodiversity Conservation Act 1999 (EPBC Act), (1999).
39. Australia GoW. Bush Forever Volume 1: Policies, principles and processes. In: Protection MfPaDoE, editor. Perth, WA.2000a.
40. Australia GoW. Bush Forever Volume 2: Directory of Bush Forever sites. In: Protection MfPaDoE, editor. Perth, WA.2000b.
41. Environment Dot. Shrublands and Woodlands of the eastern Swan Coastal Plain in Community and Species Profile and Threats Database. In: Environment Dot, editor. Canberra.2016.
42. Dhakal SP. Glimpses of sustainability in Perth, Western Australia: Capturing and communicating the adaptive capacity of an activist group. Consilience: The Journal of Sustainable Development. 2014;11(1):167–82.
43. Song C, Woodcock CE, Seto KC, Lenney MP, Macomber SA. Classification and Change Detection Using Landsat TM Data: When and How to Correct Atmospheric Effects? Remote Sensing of Environment. 2001;75(2):230-44. doi: https://doi.org/10.1016/S0034-4257(00)00169-3.
44. Cui L, Li G, Ren H, He L, Liao H, Ouyang N, et al. Assessment of atmospheric correction methods for historical Landsat TM images in the coastal zone: A case study in Jiangsu, China. European Journal of Remote Sensing. 2014;47(1):701-16. doi: 10.5721/EuJRS20144740.
45. Lu D, Mausel P, Brondizio E, Moran E. Assessment of atmospheric correction methods for Landsat TM data applicable to Amazon basin LBA research. International Journal of Remote Sensing. 2002;23(13):2651-71. doi: 10.1080/01431160110109642.
46. Gilmore S, Saleem A, Dewan A. Effectiveness of DOS (Dark-Object Subtraction) method and water index techniques to map wetlands in a rapidly urbanising megacity with Landsat 8 data. CEUR Workshop Proceedings. 2015;1323:100-8.
47. Nazeer M, Nichol J. Selection of atmospheric correction method and estimation of Chlorophyll-A (Chl-a) in coastal waters of Hong Kong. 3rd International Workshop on Earth Observation and Remote Sensing Applications, EORSA 2014 - Proceedings. 2014:374-8. doi: 10.1109/EORSA.2014.6927916.
48. Yusuf FR, Santoso KB, Ningam MUL, Kamal M, Wicaksono P. Evaluation of atmospheric correction models and Landsat surface reflectance product in Daerah Istimewa Yogyakarta, Indonesia. IOP Conference Series: Earth and Environmental Science. 2018;169:012004. doi: 10.1088/1755-1315/169/1/012004.
49. Dewi E, Trisakti B. COMPARING ATMOSPHERIC CORRECTION METHODS FOR LANDSAT OLI DATA. International Journal of Remote Sensing and Earth Sciences (IJReSES). 2017;13:105. doi: 10.30536/j.ijreses.2016.v13.a2472.
50. Mohajane M, Essahlaoui A, Oudija F, Hafyani ME, Hmaidi AE, Ouali AE, et al. Land Use/Land Cover (LULC) Using Landsat Data Series (MSS, TM, ETM+ and OLI) in Azrou Forest, in the Central Middle Atlas of Morocco. Environments. 2018;5(12):131. PubMed PMID: doi:10.3390/environments5120131.
51. Gu D, Gillespie A. Topographic Normalization of Landsat TM Images of Forest Based on Subpixel Sun–Canopy–Sensor Geometry. Remote Sensing of Environment. 1998;64(2):166-75. doi: https://doi.org/10.1016/S0034-4257(97)00177-6.
52. Herring JWaD. Measuring Vegetation (NDVI & EVI) 2000. Available from: https://earthobservatory.nasa.gov/features/MeasuringVegetation.
53. Cha S-Y, Park C-H. The utilization of google earth images as reference data for the multitemporal land cover classification with MODIS data of North Korea.2007.
54. Pascual-Hortal L, Saura S. Comparison and development of new graph-based landscape connectivity indices: towards the priorization of habitat patches and corridors for conservation. Landscape Ecology. 2006;21(7):959-67. doi: 10.1007/s10980-006-0013-z.
55. Saornil J, Gutiérrez J, Hernando A, Abril A, Sánchez B, Aparicio M. Structural connectivity as an indicator of species richness and landscape diversity in Castilla y León (Spain)2019. 286-97 p.
56. Wickham JD, Riitters KH, Wade TG, Vogt P. A national assessment of green infrastructure and change for the conterminous United States using morphological image processing. 2010.

57. Shi X, Qin M. Research on the Optimization of Regional Green Infrastructure Network. Sustainability. 2018;10(12):4649. PubMed PMID: doi:10.3390/su10124649.
58. MacLachlan A, Biggs E, Roberts G, Boruff B. Urban Growth Dynamics in Perth, Western Australia: Using Applied Remote Sensing for Sustainable Future Planning. Land. 2017;6(1):9. PubMed PMID: doi:10.3390/land6010009.
59. WorldPopulationReview. Perth Population 2019 2019 [21 February 2019]. Available from: http://worldpopulationreview.com/world-cities/perth-population/.
60. Commission WAP. Urban Growth Monitor Perth Methopolitan, Peel and Greater Bunbury Regions. In: WAPC WAPC, editor. Gordon Stephenson House 140 William Street Perth WA 6000: the Western Australian Planning Commission; 2019.
61. Authority MRP. The Corridor Plan for Perth. In: Authority MRP, editor. Perth, WA1970.
62. Development DoPaU. Metroplan: A planning strategy for the Perth Metropolitan Region. In: Development DoPaU, editor. Perth, WA.1990.
63. Bureau of Infrastructure TaREB. Population growth, jobs growth and commuting flows in Perth, . In: Transport DoIa, editor. Canberra ACT 2010.
64. Department of Biodiversity CaA. A methodology for the evaluation of wetlands on the Swan Coastal Plain, draft prepared by the Wetlands Section of the Department of Biodiversity. In: Regulation CaAatUWBotDoWaE, editor. Perth, WA2017.
65. Saura S, Vogt P, Velázquez J, Hernando A, Tejera R. Key structural forest connectors can be identified by combining landscape spatial pattern and network analyses. Forest Ecology and Management. 2011;262(2):150-60. doi: https://doi.org/10.1016/j.foreco.2011.03.017.
66. Morjan CL, Rieseberg LH. How species evolve collectively: implications of gene flow and selection for the spread of advantageous alleles. Molecular ecology. 2004;13(6):1341-56. doi: 10.1111/j.1365-294X.2004.02164.x. PubMed PMID: 15140081.
67. Green T. Perth's Urban Forest: A WA2.0 Project 2018.
68. Commission WAP. Perth and Peel @ 3.5 Million. In: Department of Planning GoWA, editor. Perth,WA, Australia2015.
69. Wickham J, Riitters K, Vogt P, Costanza J, Neale A. An inventory of continental U.S. terrestrial candidate ecological restoration areas based on landscape context2017.
70. Neylan J. Improving The Environmental Management of New South Wales Golf courses. In: NSW DoEaCC, editor. 59–61 Goulburn Street, Sydney, PO Box A290.
71. Deslauriers MR, Asgary A, Nazarnia N, Jaeger JAG. Implementing the connectivity of natural areas in cities as an indicator in the City Biodiversity Index (CBI). Ecological Indicators. 2017. doi: https://doi.org/10.1016/j.ecolind.2017.02.028.
72. Dobbs EK, Potter DA. Naturalized habitat on golf courses: source or sink for natural enemies and conservation biological control? Urban Ecosystems. 2016;19(2):899-914. doi: 10.1007/s11252-015-0521-1.
73. Saarikivi J, Tähtinen S, Malmberg S, Kotze DJ. Converting land into golf courses – effects on ground beetles (Coleoptera, Carabidae). Insect Conservation and Diversity. 2015;8(3):247-51. doi: 10.1111/icad.12103.
74. Chester ET, Robson BJ. Anthropogenic refuges for freshwater biodiversity: Their ecological characteristics and management. Biological Conservation. 2013;166(Supplement C):64-75. doi: https://doi.org/10.1016/j.biocon.2013.06.016.
75. Saarikivi J, Idström L, Venn S, Niemelä J, Kotze DJ. Carabid beetle assemblages associated with urban golf courses in the greater Helsinki area. European Journal of Entomology. 2010;107(4):553-61. PubMed PMID: 814377100.
76. Hudson M-AR, Bird DM. Recommendations for design and management of golf courses and green spaces based on surveys of breeding bird communities in Montreal. Landscape and Urban Planning. 2009;92(3):335-46. doi: https://doi.org/10.1016/j.landurbplan.2009.05.017.
77. Colding J, Lundberg J, Lundberg S, Andersson E. Golf courses and wetland fauna. Ecological Applications. 2009;19(6):1481-91. doi: 10.1890/07-2092.1.

78. Burgin S, Wotherspoon D. The potential for golf courses to support restoration of biodiversity for BioBanking offsets. Urban Ecosystems. 2009;12(2):145-55. doi: 10.1007/s11252-008-0076-5.

79. Tanner RA, Gange AC. Effects of golf courses on local biodiversity. Landscape and Urban Planning. 2005;71(2):137-46. doi: https://doi.org/10.1016/j.landurbplan.2004.02.004.

80. Angiuli E, Trianni G. Urban Mapping in Landsat Images Based on Normalized Difference Spectral Vector. IEEE Geoscience and Remote Sensing Letters. 2014;11(3):661-5. doi: 10.1109/LGRS.2013.2274327.

81. Wu S-S, Qiu X, Usery EL, Wang L. Using Geometrical, Textural, and Contextual Information of Land Parcels for Classification of Detailed Urban Land Use. Annals of the Association of American Geographers. 2009;99(1):76-98. doi: 10.1080/00045600802459028.

82. Toole JL, Ulm M, González MC, Bauer D, editors. Inferring land use from mobile phone activity. Proceedings of the ACM SIGKDD international workshop on urban computing; 2012: ACM.

Formatted: Space Before: 0 pt, After: 0 pt, Line spacing: single

Response to reviewers

1. Requirement from the Journal

<table>
<tr><th>Comment</th><th>Response</th></tr>
<tr><td>Please ensure that your manuscript meets PLOS ONE's style requirements, including those for file naming. The PLOS ONE style templates can be found at</td><td></td></tr>
<tr><td>We note that Figure 1 in your submission contain map/satellite images which may be copyrighted. All PLOS content is published under the Creative Commons Attribution License (CC BY 4.0), which means that the manuscript, images, and Supporting Information files will be freely available online, and any third party is permitted to access, download, copy, distribute, and use these materials in any way, even commercially, with proper attribution. For these reasons, we cannot publish previously copyrighted maps or satellite images created using proprietary data, such as Google software (Google Maps, Street View, and Earth).
We require you to either (1) present written permission from the copyright holder to publish these figures specifically under the CC BY 4.0 license, or (2) remove the figures from your submission:
(1). You may seek permission from the original copyright holder of Figure 1 to publish the content specifically under the CC BY 4.0 license.
(2). If you are unable to obtain permission from the original copyright holder to publish these figures under the CC BY 4.0 license or if the copyright holder’s requirements are incompatible with the CC BY 4.0 license, please either i) remove the figure or ii) supply a replacement figure that complies with the CC BY 4.0 license. Please check copyright information on all replacement figures and update the figure caption with source information. If applicable, please specify in the figure caption text when a figure is similar but not identical to the original image and is therefore for illustrative purposes only.
The following resources for replacing copyrighted map figures may be helpful:
USGS National Map Viewer (public domain): http://viewer.nationalmap.gov/viewer/
The Gateway to Astronaut Photography of Earth (public domain): http://eol.jsc.nasa.gov/sseop/clickmap/
Maps at the CIA (public domain): https://www.cia.gov/library/publications/the-world-</td><td>We have replaced the background of figure 1 with a map (Landsat 8) from USGS EROS (Earth Resources Observatory and Science (EROS) Center) in the public domain: http://eros.usgs.gov/#</td></tr>
</table>

factbook/index.html and https://www.cia.gov/library/publications/cia-maps-publications/index.html<br>NASA Earth Observatory (public domain): http://earthobservatory.nasa.gov/<br>Landsat: http://landsat.visibleearth.nasa.gov/<br>USGS EROS (Earth Resources Observatory and Science (EROS) Center) (public domain): http://eros.usgs.gov/#<br>Natural Earth (public domain): http://www.naturalearthdata.com/	
We note that one or more of the authors are employed by a commercial company: ArborCarbon Pty Ltd<br><br>1. Please provide an amended Funding Statement declaring this commercial affiliation, as well as a statement regarding the Role of Funders in your study. If the funding organization did not play a role in the study design, data collection and analysis, decision to publish, or preparation of the manuscript and only provided financial support in the form of authors' salaries and/or research materials, please review your statements relating to the author contributions, and ensure you have specifically and accurately indicated the role(s) that these authors had in your study. You can update author roles in the Author Contributions section of the online submission form.<br><br>Please also include the following statement within your amended Funding Statement.<br><br>"The funder provided support in the form of salaries for authors [insert relevant initials], but did not have any additional role in the study design, data collection and analysis, decision to publish, or preparation of the manuscript. The specific roles of these authors are articulated in the 'author contributions' section."<br><br>If your commercial affiliation did play a role in your study, please state and explain this role within your updated Funding Statement.	**Funding Statement**<br><br>Ms Thu Nguyen received scholarships from the Vietnam International Education Department (VIED) and Murdoch University to pursue her PhD degree at Murdoch University where this study was carried out. VIED did not play a role in the study design, data collection and analysis, decision to publish, or preparation of the manuscript.<br><br>Murdoch University provided support to Professors Bernard Dell and Richard Harper in the form of salaries and provided support to Ms. Thu Nguyen in the form of facilities, but did not play a role in the study design, data collection and analysis, decision to publish, or preparation of the manuscript.

	ArborCarbon provided financial support for A/Professor Paul Barber, but did not have any additional role in the study design, data collection and analysis, decision to publish, or preparation of the manuscript. The specific roles of these authors are articulated in the 'author contributions' section. ~~This project was funded by Murdoch University. The senior author Ms Thu Nguyen receives a joint scholarship from the Vietnam International Education Department and Murdoch University. Murdoch University provides support to Professors Bernard Dell and Richard Harper in the form of salaries. ArborCarbon does not provide any financial support for the project.~~
2. Please also provide an updated Competing Interests Statement declaring this commercial affiliation along with any other relevant declarations relating to employment, consultancy, patents, products in development, or marketed products, etc.	**Competing Interests Statement**<br>A/Prof. Paul Barber is employed by the commercial entity

Within your Competing Interests Statement, please confirm that this commercial affiliation does not alter your adherence to all PLOS ONE policies on sharing data and materials by including the following statement: "This does not alter our adherence to PLOS ONE policies on sharing data and materials.” (as detailed online in our guide for authors http://journals.plos.org/plosone/s/competing-interests) . If this adherence statement is not accurate and there are restrictions on sharing of data and/or materials, please state these. Please note that we cannot proceed with consideration of your article until this information has been declared.<br><br>Please include both an updated Funding Statement and Competing Interests Statement in your cover letter. We will change the online submission form on your behalf.<br><br>Please know it is PLOS ONE policy for corresponding authors to declare, on behalf of all authors, all potential competing interests for the purposes of transparency. PLOS defines a competing interest as anything that interferes with, or could reasonably be perceived as interfering with, the full and objective presentation, peer review, editorial decision-making, or publication of research or non-research articles submitted to one of the journals. Competing interests can be financial or non-financial, professional, or personal. Competing interests can arise in relationship to an organization or another person. Please follow this link to our website for more details on competing interests: http://journals.plos.org/plosone/s/competing-interests	**Competing Interests Statement:**<br>ArborCarbon who provides in-kind support only through the employment of the co-author A/Prof. Paul Barber and access to facilities, equipment and software. This commercial affiliation does not alter our adherence to all PLOS ONE policies in sharing data and materials.<br>Profs Dell, Profs Harper, and other authors have no competing interests.

2. Reviewer #1

Comment	Response
Page 1, row 26 (abstract): consider being specific (last three decades, 1988-2018)	Agreed and edited:<br>“last three decades, 1988-2018”
Page 1, row 29 and 30 (abstract): I wonder if due to their specific land use golf course are somewhat non conductive to habitat suitability?	We have clarified this in the text. In Australia and other parts of the world, many golf courses include significant non-play areas, which comprise plantations or natural vegetation. Those are shown to be suitable for habitat of wildlife and biodiversity in previous studies and government policies:<br>- Hodgkison, S. C., Hero, J. M., & Warnken, J. (2007). The conservation value of suburban golf courses in a rapidly urbanising region of Australia. *Landscape and Urban Planning, 79*(3), 323-337. doi:https://doi.org/10.1016/j.landurbplan.2006.03.009

	- Hudson, M.-A. R., & Bird, D. M. (2009). Recommendations for design and management of golf courses and green spaces based on surveys of breeding bird communities in Montreal. *Landscape and Urban Planning, 92*(3), 335-346.<br>- Tanner, R. A., & Gange, A. C. (2005). Effects of golf courses on local biodiversity. *Landscape and Urban Planning*, *71*(2), 137-146. doi:https://doi.org/10.1016/j.landurbplan.2004.02.004
I am not too sure about your last statement in the Abstract that suggest "we need to consider before clearing for urban development". An already developed area becomes a fair game in my opinion and you may need to qualify that statement. Will check how you substantiated this?	We have edited this section of the Abstract to:<br><br>"Our findings suggest that for future urban expansion, urban planning needs to more carefully consider factors that affect connectivity in the landscape such as preserving functional forest remnants and encouraging reforestation."<br><br>This takes into account the circumstances where urban areas expand into forested areas, or where reforestation occurs on previously deforested land. Our paper clearly shows that remnant forests dispersed across urban areas are essential for biodiversity conservation.
Page 2, row 44 (Introduction): suggesting of replace "delivered" by "derived:	Agreed and edited
Page 2, row 47 (Introduction):<br>While there are few publications about these topics, most of them tend to be rather tenuous at best.<br>The general idea of large urban environments being non conductive to healthy lifestyle is well founded but maybe beyond the amount of green?	This is now deleted
Page 2, row 49 (Introduction):<br>"natural disasters"<br>Floods for example?<br><br>But they me be conductive to other risks and hazards too?	Have modified:<br>"some kinds of natural disaster for example food"
Page 2, row 55 (Introduction):<br>"Fragmentation": This is almost always inevitable	Have edited:<br>Therefore, urban development that requires clearing of vegetation, due to reduction in vegetation cover, habitat loss and fragmentation is a threat to biodiversity

Page 3, row 84: REF 29 is not correct? Look for Tucker, Huete, Myneni, Prince, for an NDVI reference	The Reference: 29 is deleted and the reference Tucker 1979 and Myneni et al 1996 are now added for NDVI analysis.
Page 3, row 88: "Then''	Deleted
Page 4, row 104 "Study area": You need to expand this section a bit more to create a anchor for the motivations and objectives?<br>Not sure what else to say, but things like what is so special besides the nmbr of golf courses? how it is biodiverse? what do you hope to achieve? are there any plans to change things? etc.	- In the Introduction, the background of the study area has been involved the information about why we choose this study area. Because it lies within a rapidly urbanizing biodiversity hotspot in Australia and has important role in regional biodiversity connectivity, which is linked to the aims, motivation, objectives of this study.<br>- Additional text added to the study area section:<br><br>"The study area belongs to the Perth Metropolitan region covering four sub-regions (North West, Middle Central, Inner Central and South West). Perth has a Mediterranean-type temperate climate with a hot and dry summer, and a cold and rainy season occurring between May and October [32]. Under future climate-change scenarios, this area is projected to experience a lower annual rainfall [33].<br>Perth belongs to the Australia's southwest corner, which is recognized as a global biodiversity "hotspot" with outstanding natural environments. Our study area occurs on the Swan Coastal Plain which is part of the southwest of Australia which in turn has the highest concentration of rare and endangered species on the entire continent (at least 1,500 endemic species). More than 6,000 species of native plants and 100 native mammals, birds, frogs and reptiles occur in this region, making it a biodiversity "hotspot" [31, 34].<br>Perth has retained a significant area of natural vegetation which has conservation significance thanks to the introduction of legislation and policies aimed primarily at protecting biophysical environmental values. This includes the Western Australian Environmental Protection Act 1986 [35]; the Federal Government's Environment Protection and Biodiversity Conservation Act 1999 [36]; and more recently, Bush Forever [37, 38], a policy which took a whole-of-government approach to identify and protect biologically significant bushland and wetlands within the Perth metropolitan area. However, Perth has experienced extensive urban development since the 1980s [39]. The expansive growth of the Perth metropolitan footprint has contributed to the loss of biodiversity, together with the ecosystem services provided by natural areas. Together with urbanization, the golf industry has expanded contributing to a growing proportion of Perth's urban green space with 34 golf courses in the study area (Figure 1). "

Page 4, row 108: what does unsustainable mean here?	" unsustainable rate" is now deleted and the sentence has been edited.
Page 4, row 123: Please correct, it is the Earth Resources Observation and Science Center (EROS).	It is now corrected
Page 4 and 5: **Landsat data and pre-processing**<br>There are issues with the classification based on images from the month of December only (Summer). There was little in the way of what exact dates, what data, how did you remove clouds, etc... This is critical since it will impact the change detection which is based on NDVI, which is very sensitive to noise in the data and the time the data came from? You will need to elaborate on your exact methods and data analysis here.	More details of the input data sets for the classification and analysis are added to the text . We chose all the free cloud images or at least cloud-free at the study area, so there is no requirement for removing cloud.
Page 5, rows 127-133: USGS now distributes fully research ready data (fully atm. corrected using standard and sophisticated methods that surpasses DOS)<br>There was hardly any useful information on how the Atm. Correction was carried. You need to clarify this further so readers can replicate and or understand better.	The best atmospheric correction varies on each object (Yusuf et al. 2018). We used dark object subtraction (DOS) to correct atmospheric scattering as this method is still approved appropriate and reliable one, which is consistent with previous studies:<br>Mohajane, M., Essahlaoui, A., Oudija, F., Hafyani, M. E., Hmaidi, A. E., Ouali, A. E., . . . Teodoro, A. C. (2018). Land Use/Land Cover (LULC) Using Landsat Data Series (MSS, TM, ETM+ and OLI) in Azrou Forest, in the Central Middle Atlas of Morocco. *Environments*, *5*(12), 131.<br><br>Dewi, E., & Trisakti, B. (2017). COMPARING ATMOSPHERIC CORRECTION METHODS FOR LANDSAT OLI DATA. *International Journal of Remote Sensing and Earth Sciences (IJReSES), 13*, 105. doi:10.30536/j.ijreses.2016.v13.a2472<br>Cui, L., Li, G., Ren, H., He, L., Liao, H., Ouyang, N., & Zhang, Y. (2014). Assessment of atmospheric correction methods for historical Landsat TM images in the coastal zone: A case study in Jiangsu, China. *European Journal of Remote Sensing, 47*(1), 701-716. doi:10.5721/EuJRS20144740<br><br>Gilmore, S., Saleem, A., & Dewan, A. (2015). Effectiveness of DOS (Dark-Object Subtraction) method and water index techniques to map

	wetlands in a rapidly urbanising megacity with Landsat 8 data. *CEUR Workshop Proceedings, 1323*, 100-108.<br>Nazeer, M., & Nichol, J. (2014). Selection of atmospheric correction method and estimation of Chlorophyll-A (Chl-a) in coastal waters of Hong Kong. *3rd International Workshop on Earth Observation and Remote Sensing Applications, EORSA 2014 - Proceedings*, 374-378. doi:10.1109/EORSA.2014.6927916<br><br>Yusuf, F. R., Santoso, K. B., Ningam, M. U. L., Kamal, M., & Wicaksono, P. (2018). Evaluation of atmospheric correction models and Landsat surface reflectance product in Daerah Istimewa Yogyakarta, Indonesia. *IOP Conference Series: Earth and Environmental Science, 169*, 012004. doi:10.1088/1755-1315/169/1/012004
Page 5, row 138: Reword the caption. This is a processing pipelines or flowchart.	We use the term flowchart as we suggest it is more appropriate
Page 5, row 140: Why here?	It is now deleted
Page 5, row 149: For Landsat we usually list the actual band numbers to avoid any confusion?	The equation is edited
Page 6, row 153: The first reason for high NDVI is that RED is consumed by chlorophyll to drive photosynthesis.	This is now edited
Page 6, row 156: “three classes”, that are?	Three classes are added
Page 8, row 212: Grammar error	It is now corrected
Page 8, row 230: where did this nmbr come from?	The number is now corrected
I suggest you improve the captions of all figures - Elaborate on caption and make them read better?	We have edited the captions
Page 10, row 243, suggestion f or the title of table 3.	It is now edited
The concept of vegetation cover gain in urbanized areas is a bit of a misnomer, as how would that happen? is it urban abandonment? or other mechanism? Please clarify.	We have added text to describe this in the first paragraph of Sect 4.1.
For the sake of clarity and completeness I highly suggest you define these terms (Core, Bridge, etc.) and what they capture	Agreed and all of those terms are redefined in Table 1 in the Methods section.

and mean?<br><br>The reference is great but forces the reader to read another paper, where a simple short definition may suffice.	
I also find the reference to " Golf courses" as a fully functional and reliable habitats a bit confusing considering their high traffic and human impacts? Please clarify.	We have added to the Introduction: Golf courses are established for recreational purposes. The whole area of golf courses are a mix of bushland, fairways and infrastructure. Though they are not fully ecologically functional as with a natural parks.<br>Several studies investigated the condition of vegetation inside the golf courses and indicated that those natural areas are significantly important to biodiversity conservation. Therefore, together with the natural reserves, golf courses can play some roles as off-reserve sites for biodiversity conservation in urban landscapes.
Page 10, 256: Is it necessary to abbreviate here?	Yes it is, we did abbreviate "Vegetation cover area (VCA)" for short title of Table 5.
Page 12, row 293: These terms must be defined for clarity.	I have added the definition of those terms in the new Table 1
Page 13, row 315: "74 to 92 ha": Thousand?	Agreed and corrected
Page 14, row 342: "Cores and bridges" this needs to be defined for the sake of a general audience.	Agreed and all of those terms are redefined in the table … in the Method section.
Page 15, row 368: "2075": That is assuming an ever increasing numbers?	Agreed.
Page 16, Row 392-397: I wonder due to their specific and heavy use could golf course really be paralleled to natural veg. cover?	As mentioned above, previous studies about the golf courses in Australia and other counties have shown that the non-playing areas in many golf courses are the planted vegetation or natural bushes, which are suitable as habitats in urban landscapes.
Generally the manuscript is well written and easy to read and understand, but it lacked critical technical information and clarift in some key areas (Data analysis, Habitat analysis). I suggest you address these shortcoming.	Agreed, We have edited and bolstered the data analysis and habitat analysis components.

3. Reviewer #2

**Comment**	**Response**
This study do not intend to explain the 'dynamic'- nets effect of many factors	Agreed.

such as climate, abiotic environment, biotic interaction, disturbance history etc. In vegetation cover over Perth metropolitan, but seek to describe 'only' the spatio-temporal land use change pattern associated with urbanization in the area. Hence, the title should be reframe to capture only this aspect.	The title is edited: "Vegetation trends associated with urban development: The role of golf courses."

4. Reviewer #3

**Comment**	**Response**
Kindly improve the map resolution of all figures	It is improved
The accuracy assessment of the classification is not exit the manuscript, it is needed to be added.	It is added in the table 3
Kindly add the lat/long, scale, and north arrow on the border of layout.	It is added

5. Reviewer #4

**Comment**	**Response**
Golf courses have potential in habitat but to think they are superior in habitat protection than natural areas is the problem I have.<br>I also think you kept moving forward and back on the focus of the study. Please refer to my comments attached.	Agreed. See response to Reviewer 1, above.
Page 1, line 58: "urban matrix": I guess you mean urban vegetation matrix	Agreed and edited
Line 206 "in their surrounding regions.": Have to be clear on the specific surrounding regions. Natural or builtup	Changed from "surrounding regions " to "surrounding green spaces"
Line 210, 212-213, "Land use": There is differences between land use and land cover. What you have is cover rather than use	Agreed and edited
Line 214-215, "However, the urban	Agreed and edited: increased 10% from 1988 to 2018

footprint of built up and bare land area had increased 10% by 2018.<br>215”: Increased from 1988 or 1998	
Line 218-219, “Accuracy assessment”: It should come first before the results so we know the results we are dealing with	Agreed and edited
Line 226, Figure 3: After seeing Figure 3. Am a bit sceptic about the results for the Golf Course? Reflecting back on the methodology used to derive Golf Course in line 180-183. Do you think Landsat image of 30*30m or 15*15 for Landsat 8 did a good job of classifying a small zones like the park. There is generalisation when the study area is big which is acceptable but when zoomed in to a local scale the generalisation will be too high. You can try mapping it with the google earth but the problem is the golf courses are too many. So try mapping a few to make your point	We have added a new figure 4, which shows the details of underlying Landsat data and classification at the fine scale for one of the golf courses.
Line 253 “Two types of landscape that …….”: types of landscape matrix or index?	Agreed and edited to “ two MSPA classes”
Line 306 – 307 “not address the issues of vegetation dynamics, nor the biodiversity and structure of the vegetation and habitat connectivity”: If the work did not then what is the golf course play a part in biodiversity connectivity meant for.<br>Is biodiversity connectivity and habitat connectivity highly mutually exclusive?	Agreed and edited: the writing was not clear here and may have caused the confusion:.<br>A previous study has documented the increase in the urban footprint of Perth using multi-temporal urban expansion statistics derived from satellite imagery (MacLachlan, Biggs, Roberts, & Boruff, 2017); However, their work did not address the issues of vegetation dynamics, nor the biodiversity and structure of the vegetation and habitat connectivity in the Perth Metropolitan Region. Therefore, our study has worked to fill this gap. We have now pointed this out in the first paragraph of the Discussion.
Line 343-345, “The reduction<br>of these areas in our study indicates the high impact of urbanization on habitat<br>344<br>connectivity<br>345”:<br>How do you reconcile line 344 to 345 and 306-307?	Agreed and edited to remove this issue:<br>“As only cores (the stepping stones between forest habitat patches) and bridges (the structural corridors to link core areas) can contribute to the connectivity between the habitat areas in the landscape [63], the reduction of these areas in Perth associated with urbanization throughout the time indicates the high impact of urban development on habitat connectivity..
