# Supplementary material for: Vegetation trends associated with urban development: The role of golf courses
Source: PLoS One. 2020 Feb 5;15(2):e0228090. doi: 10.1371/journal.pone.0228090 (PMC7001943; doi:10.1371/journal.pone.0228090)
Supplement: Attachment — Submitted filename: Comments.docx [file pone.0228090.s002.docx]

| Line | Statement | Comment |
| --- | --- | --- |
| 58 | Urban matrix | I guess you mean urban vegetation matrix |
| 67-68 | However, golf courses in urban settings create large green-area habitats,  which even surpass many nature reserves in size | This sentence I think is highly incorrect |
| 206 | in their surrounding regions. | Have to be clear on the specific surrounding regions. Natural or builtup |
| 210, 2012-213 | Land use | There is differences between land use and land cover. What you have is cover rather than use |
| 214-215 | However, the urban  footprint of built up and bare land area had increased 10% by 2018.  215 | Increased from 1988 or 1998 |
| 218-219 | Accuracy assessment | It should come first before the results so we know the results we are dealing with |
| 226 | Figure 3 | After seeing Figure 3. Am a bit sceptic about the results for the Golf Course? Reflecting back on the methodology used to derive Golf Course in line 180-183. Do you think Landsat image of 30*30m or 15*15 for Landsat 8 did a good job of classifying a small zones like the park. There is generalisation when the study area is big which is acceptable but when zoomed in to a local scale the generalisation will be too high. You can try mapping it with the google earth but the problem is the golf courses are too many. So try mapping a few to make your point |
| 253 | Two types of landscape that ……. | types of landscape matrix or index |
| 306-307 | However, this study did not address the issues of vegetation dynamics, nor the biodiversity and structure of the vegetation and habitat connectivity | If the work did not then what is the golf course play a part in biodiversity connectivity meant for.  Is biodiversity connectivity and habitat connectivity highly mutually exclusive |
| 343-345 | The reduction  of these areas in our study indicates the high impact of urbanization on habitat  344  connectivity  345 | How do you reconcile line 344 to 345 and 306-307 |
