# Supplementary material for: Vegetation trends associated with urban development: The role of golf courses
Source: PLoS One. 2020 Feb 5;15(2):e0228090. doi: 10.1371/journal.pone.0228090 (PMC7001943; doi:10.1371/journal.pone.0228090)
Supplement: Attachment — Submitted filename: Response to reviewers.docx [file pone.0228090.s003.docx]

**Response to reviewers**

**1. Requirement from the Journal**

| **Comment** | **Response** |
| --- | --- |
| Please ensure that your manuscript meets PLOS ONE's style requirements, including those for file naming. The PLOS ONE style templates can be found at |  |
| - We note that Figure 1 in your submission contain map/satellite images which may be copyrighted. All PLOS content is published under the Creative Commons Attribution License (CC BY 4.0), which means that the manuscript, images, and Supporting Information files will be freely available online, and any third party is permitted to access, download, copy, distribute, and use these materials in any way, even commercially, with proper attribution. For these reasons, we cannot publish previously copyrighted maps or satellite images created using proprietary data, such as Google software (Google Maps, Street View, and Earth). - We require you to either (1) present written permission from the copyright holder to publish these figures specifically under the CC BY 4.0 license, or (2) remove the figures from your submission:   (1).    You may seek permission from the original copyright holder of Figure 1 to publish the content specifically under the CC BY 4.0 license.  (2).    If you are unable to obtain permission from the original copyright holder to publish these figures under the CC BY 4.0 license or if the copyright holder’s requirements are incompatible with the CC BY 4.0 license, please either i) remove the figure or ii) supply a replacement figure that complies with the CC BY 4.0 license. Please check copyright information on all replacement figures and update the figure caption with source information. If applicable, please specify in the figure caption text when a figure is similar but not identical to the original image and is therefore for illustrative purposes only.  The following resources for replacing copyrighted map figures may be helpful:  USGS National Map Viewer (public domain): <http://viewer.nationalmap.gov/viewer/>  The Gateway to Astronaut Photography of Earth (public domain): <http://eol.jsc.nasa.gov/sseop/clickmap/>  Maps at the CIA (public domain): <https://www.cia.gov/library/publications/the-world-factbook/index.html> and <https://www.cia.gov/library/publications/cia-maps-publications/index.html>  NASA Earth Observatory (public domain): <http://earthobservatory.nasa.gov/>  Landsat: <http://landsat.visibleearth.nasa.gov/>  USGS EROS (Earth Resources Observatory and Science (EROS) Center) (public domain): [http://eros.usgs.gov/#](http://eros.usgs.gov/)  Natural Earth (public domain): <http://www.naturalearthdata.com/> | We have replaced the background of figure 1 with a map (Landsat 8) from USGS EROS (Earth Resources Observatory and Science (EROS) Center) in the public domain: [http://eros.usgs.gov/#](http://eros.usgs.gov/) |
| We note that one or more of the authors are employed by a commercial company: ArborCarbon Pty Ltd   1. Please provide an amended Funding Statement declaring this commercial affiliation, as well as a statement regarding the Role of Funders in your study. If the funding organization did not play a role in the study design, data collection and analysis, decision to publish, or preparation of the manuscript and only provided financial support in the form of authors' salaries and/or research materials, please review your statements relating to the author contributions, and ensure you have specifically and accurately indicated the role(s) that these authors had in your study. You can update author roles in the Author Contributions section of the online submission form.   Please also include the following statement within your amended Funding Statement.  “The funder provided support in the form of salaries for authors [insert relevant initials], but did not have any additional role in the study design, data collection and analysis, decision to publish, or preparation of the manuscript. The specific roles of these authors are articulated in the ‘author contributions’ section.”  If your commercial affiliation did play a role in your study, please state and explain this role within your updated Funding Statement. | **Funding Statement**  Ms Thu Nguyen received scholarships from the Vietnam International Education Department (VIED) and Murdoch University to pursue her PhD degree at Murdoch University where this study was carried out. VIED did not play a role in the study design, data collection and analysis, decision to publish, or preparation of the manuscript.  Murdoch University provided support to Professors Bernard Dell and Richard Harper in the form of salaries and provided support to Ms. Thu Nguyen in the form of facilities, but did not play a role in the study design, data collection and analysis, decision to publish, or preparation of the manuscript.  ArborCarbon provided financial support for A/Professor Paul Barber, but did not have any additional role in the study design, data collection and analysis, decision to publish, or preparation of the manuscript. The specific roles of these authors are articulated in the ‘author contributions’ section. |
| 2. Please also provide an updated Competing Interests Statement declaring this commercial affiliation along with any other relevant declarations relating to employment, consultancy, patents, products in development, or marketed products, etc.  Within your Competing Interests Statement, please confirm that this commercial affiliation does not alter your adherence to all PLOS ONE policies on sharing data and materials by including the following statement: "This does not alter our adherence to  PLOS ONE policies on sharing data and materials.” (as detailed online in our guide for authors <http://journals.plos.org/plosone/s/competing-interests>) . If this adherence statement is not accurate and  there are restrictions on sharing of data and/or materials, please state these. Please note that we cannot proceed with consideration of your article until this information has been declared.  Please include both an updated Funding Statement and Competing Interests Statement in your cover letter. We will change the online submission form on your behalf.  Please know it is PLOS ONE policy for corresponding authors to declare, on behalf of all authors, all potential competing interests for the purposes of transparency. PLOS defines a competing interest as anything that interferes with, or could reasonably be perceived as interfering with, the full and objective presentation, peer review, editorial decision-making, or publication of research or non-research articles submitted to one of the journals. Competing interests can be financial or non-financial, professional, or personal. Competing interests can arise in relationship to an organization or another person. Please follow this link to our website for more details on competing interests: <http://journals.plos.org/plosone/s/competing-interests> | **Competing Interests Statement**  A/Prof. Paul Barber is employed by the commercial entity  **Competing Interests Statement:**  ArborCarbon who provides in-kind support only through the employment of the co-author A/Prof. Paul Barber and access to facilities, equipment and software.  This commercial affiliation does not alter our adherence to all PLOS ONE policies in sharing data and materials.  Profs Dell, Profs Harper, and other authors have no competing interests. |

**2. Reviewer #1**

| **Comment** | **Response** |
| --- | --- |
| Page 1, row 26 (abstract): consider being specific (last three decades, 1988-2018) | Agreed and edited:  “last three decades, 1988-2018” |
| Page 1, row 29 and 30 (abstract): I wonder if due to their specific land use golf course are somewhat non conductive to habitat suitability? | We have clarified this in the text. In Australia and other parts of the world, many golf courses include significant non-play areas, which comprise plantations or natural vegetation. Those are shown to be suitable for habitat of wildlife and biodiversity in previous studies and government policies:   - Hodgkison, S. C., Hero, J. M., & Warnken, J. (2007). The conservation value of suburban golf courses in a rapidly urbanising region of Australia. *Landscape and Urban Planning, 79*(3), 323-337. doi:<https://doi.org/10.1016/j.landurbplan.2006.03.009> - Hudson, M.-A. R., & Bird, D. M. (2009). Recommendations for design and management of golf courses and green spaces based on surveys of breeding bird communities in Montreal. *Landscape and Urban Planning, 92*(3), 335-346. - Tanner, R. A., & Gange, A. C. (2005). Effects of golf courses on local biodiversity. *Landscape and Urban Planning, 71*(2), 137-146. doi:<https://doi.org/10.1016/j.landurbplan.2004.02.004> |
| I am not too sure about your last statement in the Abstract that suggest "we need to consider before clearing for urban development". An already developed area becomes a fair game in my opinion and you may need to qualify that statement. Will check how you substantiated this? | We have edited this section of the Abstract to:  “Our findings suggest that for future urban expansion, urban planning needs to more carefully consider factors that affect connectivity in the landscape such as preserving functional forest remnants and encouraging reforestation.”  This takes into account the circumstances where urban areas expand into forested areas, or where reforestation occurs on previously deforested land. Our paper clearly shows that remnant forests dispersed across urban areas are essential for biodiversity conservation. |
| Page 2, row 44 (Introduction): suggesting of replace “delivered” by “derived: | Agreed and edited |
| Page 2, row 47 (Introduction):  While there are few publications about these topics, most of them tend to be rather tenuous at best.  The general idea of large urban environments being non conductive to healthy lifestyle is well founded but maybe beyond the amount of green? | This is now deleted |
| Page 2, row 49 (Introduction):  “natural disasters”  Floods for example?  But they me be conductive to other risks and hazards too? | Have modified:  “some kinds of natural disaster for example food” |
| Page 2, row 55 (Introduction): “Fragmentation”: This is almost always inevitable | Have edited:  Therefore, urban development that requires clearing of vegetation, due to reduction in vegetation cover, habitat loss and fragmentation is a threat to biodiversity |
| Page 3, row 84: REF 29 is not correct? Look for Tucker, Huete, Myneni, Prince, for an NDVI reference | The Reference: 29 is deleted and the reference Tucker 1979 and Myneni et al 1996 are now added for NDVI analysis. |
| Page 3, row 88: “Then’’ | Deleted |
| Page 4, row 104 “Study area”: You need to expand this section a bit more to create a anchor for the motivations and objectives?  Not sure what else to say, but things like what is so special besides the nmbr of golf courses? how it is biodiverse? what do you hope to achieve? are there any plans to change things? etc. | - In the Introduction, the background of the study area has been involved the information about why we choose this study area. Because it lies within a rapidly urbanizing biodiversity hotspot in Australia and has important role in regional biodiversity connectivity, which is linked to the aims, motivation, objectives of this study.  - Additional text added to the study area section:  “The study area belongs to the Perth Metropolitan region covering four sub-regions (North West, Middle Central, Inner Central and South West). Perth has a Mediterranean-type temperate climate with a hot and dry summer, and a cold and rainy season occurring between May and October [32]. Under future climate-change scenarios, this area is projected to experience a lower annual rainfall [33].  Perth belongs to the Australia's southwest corner, which is recognized as a global biodiversity "hotspot" with outstanding natural environments. Our study area occurs on the Swan Coastal Plain which is part of the southwest of Australia which in turn has the highest concentration of rare and endangered species on the entire continent (at least 1,500 endemic species). More than 6,000 species of native plants and 100 native mammals, birds, frogs and reptiles occur in this region, making it a [biodiversity "hotspot"](https://wwf.panda.org/wwf_offices/australia/??uProjectID=AU0053) [31, 34].  Perth has retained a significant area of natural vegetation which has conservation significance thanks to the introduction of legislation and policies aimed primarily at protecting biophysical environmental values. This includes the Western Australian Environmental Protection Act 1986 [35]; the Federal Government’s Environment Protection and Biodiversity Conservation Act 1999 [36]; and more recently, Bush Forever [37, 38], a policy which took a whole-of-government approach to identify and protect biologically significant bushland and wetlands within the Perth metropolitan area. However, Perth has experienced extensive urban development since the 1980s [39]. The expansive growth of the Perth metropolitan footprint has contributed to the loss of biodiversity, together with the ecosystem services provided by natural areas. Together with urbanization, the golf industry has expanded contributing to a growing proportion of Perth’s urban green space with 34 golf courses in the study area (Figure 1). “ |
| Page 4, row 108: what does unsustainable mean here? | “ unsustainable rate” is now deleted and the sentence has been edited. |
| Page 4, row 123: Please correct, it is the Earth Resources Observation and Science Center (EROS). | It is now corrected |
| Page 4 and 5: **Landsat data and pre-processing**  There are issues with the classification based on images from the month of December only (Summer). There was little in the way of what exact dates, what data, how did you remove clouds, etc... This is critical since it will impact the change detection which is based on NDVI, which is very sensitive to noise in the data and the time the data came from? You will need to elaborate on your exact methods and data analysis here. | More details of the input data sets for the classification and analysis are added to the text . We chose all the free cloud images or at least cloud-free at the study area, so there is no requirement for removing cloud. |
| Page 5, rows 127-133: USGS now distributes fully research ready data (fully atm. corrected using standard and sophisticated methods that surpasses DOS)  There was hardly any useful information on how the Atm. Correction was carried. You need to clarify this further so readers can replicate and or understand better. | The best atmospheric correction varies on each object (Yusuf et al. 2018). We used dark object subtraction (DOS) to correct atmospheric scattering as this method is still approved appropriate and reliable one, which is consistent with previous studies:  Mohajane, M., Essahlaoui, A., Oudija, F., Hafyani, M. E., Hmaidi, A. E., Ouali, A. E., . . . Teodoro, A. C. (2018). Land Use/Land Cover (LULC) Using Landsat Data Series (MSS, TM, ETM+ and OLI) in Azrou Forest, in the Central Middle Atlas of Morocco. *Environments, 5*(12), 131.  Dewi, E., & Trisakti, B. (2017). COMPARING ATMOSPHERIC CORRECTION METHODS FOR LANDSAT OLI DATA. *International Journal of Remote Sensing and Earth Sciences (IJReSES), 13*, 105. doi:10.30536/j.ijreses.2016.v13.a2472  Cui, L., Li, G., Ren, H., He, L., Liao, H., Ouyang, N., & Zhang, Y. (2014). Assessment of atmospheric correction methods for historical Landsat TM images in the coastal zone: A case study in Jiangsu, China. *European Journal of Remote Sensing, 47*(1), 701-716. doi:10.5721/EuJRS20144740  Gilmore, S., Saleem, A., & Dewan, A. (2015). Effectiveness of DOS (Dark-Object Subtraction) method and water index techniques to map wetlands in a rapidly urbanising megacity with Landsat 8 data. *CEUR Workshop Proceedings, 1323*, 100-108.  Nazeer, M., & Nichol, J. (2014). Selection of atmospheric correction method and estimation of Chlorophyll-A (Chl-a) in coastal waters of Hong Kong. *3rd International Workshop on Earth Observation and Remote Sensing Applications, EORSA 2014 - Proceedings*, 374-378. doi:10.1109/EORSA.2014.6927916  Yusuf, F. R., Santoso, K. B., Ningam, M. U. L., Kamal, M., & Wicaksono, P. (2018). Evaluation of atmospheric correction models and Landsat surface reflectance product in Daerah Istimewa Yogyakarta, Indonesia. *IOP Conference Series: Earth and Environmental Science, 169*, 012004. doi:10.1088/1755-1315/169/1/012004 |
| Page 5, row 138: Reword the caption. This is a processing pipelines or flowchart. | We use the term flowchart as we suggest it is more appropriate |
| Page 5, row 140: Why here? | It is now deleted |
| Page 5, row 149: For Landsat we usually list the actual band numbers to avoid any confusion? | The equation is edited |
| Page 6, row 153: The first reason for high NDVI is that RED is consumed by chlorophyll to drive photosynthesis. | This is now edited |
| Page 6, row 156: “three classes”, that are? | Three classes are added |
| Page 8, row 212: Grammar error | It is now corrected |
| Page 8, row 230: where did this nmbr come from? | The number is now corrected |
| I suggest you improve the captions of all figures - Elaborate on caption and make them read better? | We have edited the captions |
| Page 10, row 243, suggestion f or the title of table 3. | It is now edited |
| The concept of vegetation cover gain in urbanized areas is a bit of a misnomer, as how would that happen? is it urban abandonment? or other mechanism? Please clarify. | We have added text to describe this in the first paragraph of Sect 4.1. |
| For the sake of clarity and completeness I highly suggest you define these terms (Core, Bridge, etc.) and what they capture and mean?  The reference is great but forces the reader to read another paper, where a simple short definition may suffice. | Agreed and all of those terms are redefined in Table 1 in the Methods section. |
| I also find the reference to " Golf courses" as a fully functional and reliable habitats a bit confusing considering their high traffic and human impacts? Please clarify. | We have added to the Introduction: Golf courses are established for recreational purposes. The whole area of golf courses are a mix of bushland, fairways and infrastructure. Though they are not fully ecologically functional as with a natural parks.  Several studies investigated the condition of vegetation inside the golf courses and indicated that those natural areas are significantly important to biodiversity conservation. Therefore, together with the natural reserves, golf courses can play some roles as off-reserve sites for biodiversity conservation in urban landscapes. |
| Page 10, 256: Is it necessary to abbreviate here? | Yes it is, we did abbreviate “Vegetation cover area (VCA)” for short title of Table 5. |
| Page 12, row 293: These terms must be defined for clarity. | I have added the definition of those terms in the new Table 1 |
| Page 13, row 315: “74 to 92 ha”: Thousand? | Agreed and corrected |
| Page 14, row 342: “Cores and bridges” this needs to be defined for the sake of a general audience. | Agreed and all of those terms are redefined in the table … in the Method section. |
| Page 15, row 368: “2075”: That is assuming an ever increasing numbers? | Agreed. |
| Page 16, Row 392-397: I wonder due to their specific and heavy use could golf course really be paralleled to natural veg. cover? | As mentioned above, previous studies about the golf courses in Australia and other counties have shown that the non-playing areas in many golf courses are the planted vegetation or natural bushes, which are suitable as habitats in urban landscapes. |
| Generally the manuscript is well written and easy to read and understand, but it lacked critical technical information and clarift in some key areas (Data analysis, Habitat analysis). I suggest you address these shortcoming. | Agreed, We have edited and bolstered the data analysis and habitat analysis components. |

**3. Reviewer #2**

| **Comment** | **Response** |
| --- | --- |
| This study do not intend to explain the 'dynamic'- nets effect of many factors such as climate, abiotic environment, biotic interaction, disturbance history etc. In vegetation cover over Perth metropolitan, but seek to describe 'only' the spatio-temporal land use change pattern associated with urbanization in the area. Hence, the title should be reframe to capture only this aspect. | Agreed.  The title is edited: “Vegetation trends associated with urban development: The role of golf courses.” |

**4. Reviewer #3**

| **Comment** | **Response** |
| --- | --- |
| Kindly improve the map resolution of all figures | It is improved |
| The accuracy assessment of the classification is not exit the manuscript, it is needed to be added. | It is added in the table 3 |
| Kindly add the lat/long, scale, and north arrow on the border of layout. | It is added |

**5. Reviewer #4**

| **Comment** | **Response** |
| --- | --- |
| Golf courses have potential in habitat but to think they are superior in habitat protection than natural areas is the problem I have. I also think you kept moving forward and back on the focus of the study. Please refer to my comments attached. | Agreed. See response to Reviewer 1, above. |
| Page 1, line 58: “urban matrix”: I guess you mean urban vegetation matrix | Agreed and edited |
| Line 206 “in their surrounding regions.”: Have to be clear on the specific surrounding regions. Natural or builtup | Changed from “surrounding regions ” to “surrounding green spaces” |
| Line 210, 212-213, “Land use”: There is differences between land use and land cover. What you have is cover rather than use | Agreed and edited |
| Line 214-215, “However, the urban  footprint of built up and bare land area had increased 10% by 2018.  215”: Increased from 1988 or 1998 | Agreed and edited: increased 10% from 1988 to 2018 |
| Line 218-219, “Accuracy assessment”: It should come first before the results so we know the results we are dealing with | Agreed and edited |
| Line 226, Figure 3: After seeing Figure 3. Am a bit sceptic about the results for the Golf Course? Reflecting back on the methodology used to derive Golf Course in line 180-183. Do you think Landsat image of 30*30m or 15*15 for Landsat 8 did a good job of classifying a small zones like the park. There is generalisation when the study area is big which is acceptable but when zoomed in to a local scale the generalisation will be too high. You can try mapping it with the google earth but the problem is the golf courses are too many. So try mapping a few to make your point | We have added a new figure 4, which shows the details of underlying Landsat data and classification at the fine scale for one of the golf courses. |
| Line 253 “Two types of landscape that …….”: types of landscape matrix or index? | Agreed and edited to “ two MSPA classes” |
| Line 306 – 307 “not address the issues of vegetation dynamics, nor the biodiversity and structure of the vegetation and habitat connectivity”: If the work did not then what is the golf course play a part in biodiversity connectivity meant for.  Is biodiversity connectivity and habitat connectivity highly mutually exclusive? | Agreed and edited: the writing was not clear here and may have caused the confusion:  A previous study has documented the increase in the urban footprint of Perth using multi-temporal urban expansion statistics derived from satellite imagery (MacLachlan, Biggs, Roberts, & Boruff, 2017); However, their work did not address the issues of vegetation dynamics, nor the biodiversity and structure of the vegetation and habitat connectivity in the Perth Metropolitan Region. Therefore, our study has worked to fill this gap. We have now pointed this out in the first paragraph of the Discussion. |
| Line 343-345, “The reduction  of these areas in our study indicates the high impact of urbanization on habitat  344  connectivity  345”:  How do you reconcile line 344 to 345 and 306-307? | Agreed and edited to remove this issue:  “As only cores (the stepping stones between forest habitat patches) and bridges (the structural corridors to link core areas) can contribute to the connectivity between the habitat areas in the landscape [63], the reduction of these areas in Perth associated with urbanization throughout the time indicates the high impact of urban development on habitat connectivity.. |
