# Supplementary material for: Vegetation trends associated with urban development: The role of golf courses
Source: PLoS One. 2020 Feb 5;15(2):e0228090. doi: 10.1371/journal.pone.0228090 (PMC7001943; doi:10.1371/journal.pone.0228090)
Supplement: Attachment — Submitted filename: Response to reviewers.docx [file pone.0228090.s005.docx]

**Response to reviewer 1 and the Editor**

| 1. **Editor’s comment** | **Response** |
| --- | --- |
| Please ensure that you refer to Figure 4 in your text as, if accepted, production will need this reference to link the reader to the figure. | Agreed, the figure has been quoted in the text**.** |
| 2. **Reviewer’s comment** | **Response** |
| Few do require serious changes especially the quality of the plots, figures. | The quality of the figures in the PDF file are poor while the underlying tif files are ok.  Discussed with editor, who responded: “The compilation PDF is provided for review purposes only and the images in the PDF are low-resolution. If the figures in the PDF look acceptable, they will be fine for the review process. High-resolution versions of each image in the PDF can be accessed by clicking the blue link at the top of the page where the figure appears.” |
| some require rewording to make it clear that for example golf courses green, while is green and will be pi+6cked up by NDVI, is not the same quality green as natural and from a habitat perspective. | In the paper we have recognised the issue of Landsat’s moderate resolution (30 m x 30 m) in land cover classification. However, Landsat is the only platform that provides the opportunity to retrospectively assess vegetation trends over the last three decades.  We recognise this limitation and make this clear in the discussion section lines 412 – 425 page 19-20 of the manuscript. |
| Line 78 – 79: Not sure if this sentence adds much? | The sentence has been deleted |
| Line 85 | ‘’The most’’ is replaced by ‘’an’’ |
| Lines 86-87: Suggestion for editing the sentence “This separates green vegetation from other surfaces based on the ability of chlorophyll to absorb red light for photosynthesis and reflect the near-infrared 87 (NIR) wavelengths”. | The sentence has been edited:  “This separates green vegetation from other surfaces based on the differential absorption of red light by chlorophyll and reflection of NIR by green vegetation.” |
| Lines 126-127: comments for the title of figure 1 with source of the map. | Additional information has been added to the captions of all Figures |
| Lines 147-154: FYI : EROS/LP-DAAC does provide consistently atmospherically corrected Landsat data too? | Yes it does. However, due to the availability of Landsat images at the time steps set for our study, we finally had to use the Landsat L1T level product. Again, we have made this clear in the paper. |
| Line 157: typing error | Corrected |
| Line 167: comments for choosing words | “has consumed” is now replaced by “absorbs” |
| Line 185: Depending on the exact time of year the images are being compared, this method is fret with noise resulting from rain driven greening.  While greening is greening, it is wise to be aware of this and clarify it. | We have recognised the issue of noise related to time series analysis. We selected images from the same month in summer, which has a 7 month dry season in Perth, to reduce the noise. |
| Line 220: “analysis for the four time steps allowed us to assess the changes in habitat connectivity 219 over 30 years of  220 urbanization.” -> This is usually fine, but using synoptic dates only could be problematic as it does not capture what happen in between the dates, and that could hide other factors.  In other words the linear/smooth change assumption could be wrong here. | We know that there has been 30 years of urbanization throughout Perth and we do understand there may be other factors impacting green space between time points that are not picked up in our analysis. The main focus is the change that has occurred over the 30 years. To emphasise that our assessment is confined to single time points, we have added the word ‘step’ prior to the word ‘changes.’ |
| Line 244: The images quality is quite poor.  This is the case for most images in the manuscript. | See above and editor’s response.  I have asked the editor of the journal why the quality of figures in PDF file are pretty poor while the underlying tif files are ok. They responded: “The compilation PDF is provided for review purposes only and the images in the PDF are low-resolution. If the figures in the PDF look acceptable, they will be fine for the review process. High-resolution versions of each image in the PDF can be accessed by clicking the blue link at the top of the page where the figure appears.” |
| Line 257: I highly suggest the tables be restructured to show change in each class for all years on the same tables/Columns. This will make is easier to read and understand | We have not made changes as we prefer to use the format in Table 3 because the accuracy assessment was undertaken separately for each map that was generated for each year studied. |
| Line 294-295: Once again, it is critical that captions carry a clear and strong message. So I suggest you add more text to captions to make them convey a clear message.  Figure 6. Results of the Morphological Spatial Pattern Analysis (MSPA) for the Perth region  295 from 1988 to 2018 | Agreed. All the captions have been edited |
| Line 323: What do you mean by unsustainable rate | “Sustainable rate”: is now replaced by “fast rate” |
| Line 391: I would like for the authors to clarify that the golf courses have replaced green with another kind of green. While it does preserve green and provide some habitat, I am not too sure if it is the same quality from a habitat perspective? | Our analysis does not differentiate between the type/kind of green space as the spatial resolution of the imagery is unable to differentiate between trees, shrubs, turf, groundcovers etc.Therefore, we are not too sure either and as such we have been careful not to include the term ‘habitat’ in our discussion, but instead said that the golf courses potentially provide habitat in urban landscape based on our analysis and previous studies. It is outside of the scope of our research to provide such clarification about the quality of habitat. It is evident, however, that golf courses have performed an important role in the preservation of green space during surrounding urban development which may include some important habitats in the biodiversity hotpot city. |
